# Multi-omics reveals CXCR4 drives immune escape in colorectal cancer via metabolic reprogramming and immune microenvironment remodeling

**DOI:** 10.1038/s41419-026-08795-x

**Published:** 2026-05-04

**Authors:** Chengle Zhu, Yuting Liu, Shaopeng Xu, Ruonan Li, Jiwen Shi, Guohui Tang, Ruorong Ran, Bo Pang, Zixiang Chi, Yongxing Ding, Wenrui Wang, Qingling Yang, Changjie Chen

**Affiliations:** 1 Anhui Provincial Key Laboratory of Tumor Evolution and Intelligent Diagnosis and Treatment, Bengbu Medical University, Bengbu, China; 2 School of Laboratory Medicine, Bengbu Medical University, Bengbu, China; 3 The Third the People’s Hospital of Bengbu, Bengbu, China; 4 Department of Life Sciences, Bengbu Medical University, Bengbu, China

**Keywords:** Cancer microenvironment, Cancer metabolism, Immunosuppression

## Abstract

Colorectal cancer (CRC) is one of the most common malignant tumors with the highest incidence and mortality rates worldwide. Immune checkpoint blockade (ICB) therapy has revolutionized the landscape of cancer treatment; however, most patients with CRC gain limited benefits from it. The immunosuppressive microenvironment of CRC is an important cause of tumor progression, metastasis, and immunotherapy resistance. This study aimed to reveal the key role of chemokine receptor 4 (CXCR4) in the immunosuppressive microenvironment and glutamine metabolism reprogramming using integrated single-cell transcriptomics and metabolomics analyses. The in vivo and in vitro experiments verified that CXCR4 mediated metabolic reprogramming in CRC cells by regulating the PI3K-Akt-SMAD4 pathway. Further co-culture experiments revealed that CXCR4 promoted the polarization of tumor-associated macrophages (TAMs) to M2 type through glutamine metabolic reprogramming and induced the exhaustion of CD8^+^ T cells, thereby intensifying immune escape. The knockdown of CXCR4 significantly increased the infiltration of CD8^+^ T cells and M1 TAMs, reduced the infiltration of M2 TAMs, effectively reshaped the immunosuppressive microenvironment of CRC-bearing mice, and significantly enhanced the immunotherapeutic effect against programmed cell death protein 1 (PD-1). This study discovered a novel mechanism by which CXCR4 drove CRC immune escape through the dual-axis regulation of the “glutamine metabolism-immune microenvironment.” Targeting CXCR4 not only inhibits tumor metabolic adaptability but also reverses TAMs polarization and T cell exhaustion, thereby effectively sensitizing PD-1 inhibitors. This study provides an important theoretical basis and a highly promising new combined treatment strategy for overcoming ICB resistance in patients with CRC.

## Introduction

CRC is one of the most common malignant tumors worldwide [1]. The global cancer statistics indicate that the incidence of CRC ranks third, and its mortality rate ranks second [2]. The treatment of CRC mainly relies on traditional methods such as surgery, radiotherapy, and chemotherapy [3]. However, its mortality rate remains high due to distant metastasis, tumor heterogeneity, and drug resistance, becoming the main bottleneck of current treatment modalities [4]. The tumor microenvironment (TME) plays a crucial role in tumor progression and treatment resistance [5]. TME shows significant spatiotemporal dynamic heterogeneity with tumor progression. The spatial structure is highly nonuniform and continuously evolves over time. This complexity poses a huge challenge to TME research [6]. Single-cell RNA sequencing (scRNA-seq) technology has revealed the heterogeneity of immune cells and their complex interactions in the TME, thus providing novel insights into the treatment of CRC [7].

CXCR4 is a G protein-coupled receptor (GPCR) that selectively binds to chemokine stromal cell-derived factor 1 (SDF-1), also known as C-X-C motif chemokine ligand 12 (CXCL12) [8]. It is overexpressed in more than 23 different types of human cancers [9]. Cancer-associated fibroblasts (CAFs) and other stromal cells release large amounts of CXCL12 through autocrine or paracrine mechanisms [10]. which in turn recruits immunosuppressive cells such as regulatory T cells (Tregs), TAMs, and myeloid-derived suppressor cells (MDSCs), thereby forming an immunosuppressive microenvironment [11]. Metabolic reprogramming is a hallmark of cancer cells. It drives immune cells to produce tolerant metabolic adaptations by inducing nutrient deficiency, hypoxia, and acidosis in the TME, ultimately suppressing antitumor immune responses and promoting immune escape [12]. Glutamine is the most abundant amino acid in the body, playing a crucial role in metabolic reprogramming [13]. Glutamine addiction exists in many tumors [14]. The depletion of glutamine in TME can lead to significant changes in the phenotype of immune cells, thereby promoting tumor immune escape [15]. The metabolomics analysis in this study revealed significant changes in glutamine metabolism in the CXCR4 knockdown model. This indicated the involvement of CXCR4 in metabolic reprogramming in the TME, thereby influencing the biological functions of CRC cells. However, the specific role of CXCR4 in regulating metabolic reprogramming in the TME and the molecular mechanism by which it promotes the malignant progression of CRC cells through glutamine metabolism need clarification.

Immune checkpoint inhibitors (ICIs) have profoundly transformed the treatment landscape of cancer. Among these, PD-1/programmed death ligand 1 (PD-L1) inhibitors are used for treating various malignant tumors [16]. The PD-1 ICIs approved by the US Food and Drug Administration (FDA) can benefit patients with metastatic colorectal cancer (mCRC) having mismatch repair deficiency (dMMR) and microsatellite instability-high (MSI-H). However, it is almost ineffective for patients with the more common mismatch repair function intact (pMMR) and microsatellite stable (MSS) mCRC [17]. An effective immune response cannot be induced due to the weak immunogenicity of tumor cells and the lack of immune cell infiltration in patients with pMMR/MSS mCRC, resulting in the ineffectiveness of ICB treatment [18]. Exploring combined treatment strategies that enhance immunogenicity and reverse the immunosuppressive microenvironment is the key to overcoming ICB resistance in patients with pMMR/MSS mCRC.

This study integrated single-cell transcriptome and metabolomics analyses to clarify that CXCR4 drove macrophage M2 polarization and CD8⁺ T cell exhaustion in the immune microenvironment of CRC through the PI3K-Akt/SMAD4/glutamine metabolic axis. The targeted inhibition of CXCR4 can promote the infiltration of M1 macrophages and CD8⁺ T cells and block the reprogramming of glutamine metabolism, thereby reversing the tumor immunosuppressive microenvironment and providing a novel combined treatment strategy for clinical practice.

## Materials and methods

### Cell culture

Human CRC cells (HCT116, HT29, LOVO, SW480, and SW620), normal human intestinal epithelial cells (FHC), mouse CRC cells (CT26), and human monocytic leukemia cells (THP-1) were all purchased from the Shanghai Cell Bank of the Chinese Academy of Sciences. These cells were cultured in RPMI 1640 or Dulbecco’s modified Eagle’s medium containing 10% fetal bovine serum (FBS) at 37 °C and in the presence of 5% CO₂.

### Cell transfection and lentiviral transduction

si-SMAD4/negative control (NC) was transfected into CRC cells using Lipofectamine 2000 reagent (Invitrogen, USA). CXCR4 knockdown and NC lentiviruses were transduced into cells using HiTransG A/P infection reagent (GeneChem, China). siRNA was purchased from GenePharma Technology (Shanghai, China), whereas the lentivirus was purchased from GeneChem Biotechnology (Shanghai, China).

### RNA extraction and quantitative real-time polymerase chain reaction

The CRC cells were digested and collected using trypsin, and total RNA was extracted from them using TRIzol (Invitrogen, CA, USA). Total RNA was reverse transcribed into cDNA using reverse transcription kits (Thermo Scientific, MA, USA). cDNA was amplified by quantitative real-time polymerase chain reaction (qRT-PCR) using the qRT-PCR kit (GeneCopoeia, Guangzhou, China). The sequences of primers used in this study are detailed in Table 1. The relative mRNA expression in each group was calculated using the relative quantitative algorithm 2^−ΔΔCt^.

**Table 1:** Sequences of primers used in this study.

Gene	Forward primer	Reverse primer
GAPDH	CAGCCTCAAGATCATCAGCA	TGTGGTCATGAGTCCTTCCA
CD206	AGCCAGGGTGTGTTGCCATG	GCTTCGGTGGGTGGGTTACTC
ARG-1	GGACCTGCCCTTTGCTGACATC	TCTTCTTGACTTCTGCCACCTTGC
INOS	CAGGCGTCCACCGTCTTCATTG	ATCCCAGCCATCCACGATAATGTTG
CD86	ACGGAGTCAATGAAGATTTCCT	GATTCGGCTTCTTGTGACATAC
CD11b	CAGTGTGACATCCCGTTCTT	CACGATCAGGAGGTGGTTATG

### Western blot analysis

The cell precipitates were collected from different treatments. Radioimmunoprecipitation assay (RIPA) and phenylmethylsulfonyl fluoride (PMSF) were added to the cell precipitates in a ratio of 99:1, and the cells were lysed on ice for 30 min to extract total protein. A bicinchoninic acid assay kit (Beyotime, Shanghai, China) was used for protein quantification. Each group of 30 μg samples was subjected to 10% or 12.5% sodium dodecyl sulfate-polyacrylamide gel electrophoresis (Epizyme, Shanghai, China). After electrophoresis, the protein was transferred to a polyvinylidene fluoride membrane in an ice bath (200 mA, 120 min). Subsequently, it was sealed with the blocking solution (Beyotime, Shanghai, China) at room temperature for 2 h. After blocking, the membranes were incubated overnight at 4 °C with the following primary antibodies: PD-L1 (Proteintech, 66248-1-Ig, 1:5000), p-Akt (Cell Signaling Technology, 4060, 1:5000), Akt (Cell Signaling Technology, 4691, 1:5000), SMAD4 (Proteintech, 10231-1-AP, 1:5000), Vimentin (ABclonal, A19607, 1:5000), Snail (ABclonal, A5243, 1:1000), GLS1 (Proteintech, 12855-1-AP, 1:5000), GLUD1 (Proteintech, 14299-1-AP, 1:5000), TGF-β (Proteintech, 81746-2-RR, 1:5000), CXCL12 (Proteintech, 17402-1-AP, 1:5000), CXCR4 (Abcam, Ab124824, 1:1000). Then, the membranes were incubated at 37 °C for 2 h with the following secondary antibodies:goat anti-rabbit IgG (Biosharp, BL003A, 1:5000) or goat anti-mouse IgG (Biosharp, BL001A, 1:5000). Finally, protein detection using chemiluminescence reaction was conducted. The full-length, uncropped raw images for all Western Blot results presented in this paper are provided in the Supplementary Material (Supplementary Western Blot).

### Immunofluorescence assay

THP-1 cells were pretreated with 100 ng/mL phorbol 12-myristate 13-acetate (PMA, Beyotime, Shanghai, China) and 20 ng/mL IL-4 (Bio-Techne, USA) and then co-cultured with HCT116 cells under different treatments for 24 h. The cells were washed with phosphate-buffered saline (PBS), fixed with 4% paraformaldehyde at room temperature for 30 min, permeabilized with 0.25% Triton X-100 for 5 min, blocked with 5% bovine serum albumin for 1 h, and then incubated with primary antibodies against CD206 (Proteintech, 18704-1-AP, 1:100) or CD86 (Proteintech, 83523-4-RR, 1:100) overnight at 4 °C. After washing, they were incubated with a FITC-labeled secondary antibody (Beyotime, A0562, 1:200) at room temperature for 1 h, counterstained with DAPI (Beyotime, C1002, 1:1000) at room temperature for 5 min, and mounted with an anti-fade mounting medium. The protein expression was visualized by fluorescence microscopy.

### Enzyme-Linked Immunosorbent Assay

The co-culture supernatants of macrophages treated differently were collected in 1.5-mL EP tubes and centrifuged at 1000 × *g* for 20 min at 4 °C to remove impurities and cell debris. The supernatant was collected and the TNF-α, GzmB, IFN-γ, and IL-10 levels were detected using corresponding enzyme-linked immunosorbent assay (ELISA) kits (Elabscience, China).

### Isolation of human peripheral blood mononuclear cells

The anticoagulant whole blood was diluted with PBS in a ratio of 1:1 and carefully layered over an equal volume of Ficoll separation solution. The mixture was centrifuged at 800 × *g* for 20 min, and the white film layer located in the middle layer was carefully aspirated. The cells were resuspended in an adequate amount of PBS, centrifuged at 250 × *g* for 10 min, and then washed. After discarding the supernatant, the cell precipitate was resuspended in 2 mL of red blood cell lysis buffer and lysed at room temperature for 10 min. Then, an equal volume of PBS was added to terminate the lysis, and the mixture was centrifuged at 250 × *g* for 10 min. The supernatant was discarded, and the cell precipitate was finally resuspended in 1 mL of RPMI 1640 complete medium for subsequent experiments.

### Preparation of single-cell suspension

Mouse tumor tissues were obtained aseptically on ice and minced in pre-cooled PBS to obtain tissue blocks of approximately 1 mm³. These blocks were transferred into centrifuge tubes containing pre-cooled digestive enzymatic hydrolysates (2 mg/mL collagenase IV, 0.2 mg/mL hyaluronidase, and 40 µg/mL DNase I, all purchased from Solarbio, China). The tissues were incubated in a water bath at 37 °C for 30 min, gently inverting and mixing every 10 min. Two to three times the volume of pre-cooled complete medium containing 10% FBS was added immediately after digestion to terminate. The suspension was gently pipetted on ice and filtered successively through 70- and 40-μm sterile filters (Biosharp, China). Then, the filtrate was centrifuged at 350 × g at 4 °C for 5 min, and the supernatant was discarded. The precipitate was washed once or twice with pre-cooled PBS or buffer and then resuspended in an appropriate amount of PBS for later use.

### Flow cytometry

Single-cell suspensions, obtained either from co-culture or dissociated tumor tissues, were adjusted to an appropriate concentration. Cells were first incubated with an Fc receptor blocking solution (BioLegend, USA) to prevent nonspecific binding. Subsequently, they were stained with a cocktail of fluorescently conjugated antibodies—including anti-human CD3 (BioLegend, 300412), CD8 (BioLegend, 301008), PD-1 (BioLegend, 329903), and CD206 (BioLegend, 321105), as well as anti-mouse CD3 (BioLegend, 100219), CD8 (BioLegend, 100711), CD11b (BioLegend, 101228), CD86 (BioLegend, 159204), and CD206 (BioLegend, 141708)—for 30 min on ice and protected from light. Following incubation, cells were washed once with PBS, resuspended in PBS, and analyzed by flow cytometry.

### Detection of glutamine, glutamate, α-ketoglutarate, and ATP contents

HCT116 CRC cells were seeded in six-well plates at a density of 1 × 10⁶ cells per well. After cell attachment, the cells were treated differently and cultured for 48 h. The culture supernatant was collected, centrifuged to remove cell debris, and stored at -80 °C for subsequent analysis. In parallel, cells were harvested and counted for normalization. The levels of glutamine, glutamate, α-ketoglutarate (α-KG), and ATP were quantified using the respective commercial assay kits according to the manufacturer’s instructions. Absorbance or fluorescence was measured with a multimode microplate reader. The concentrations of each metabolite were calculated based on standard curves and normalized to the corresponding cell number.

### scRNA-seq data analysis

A mixture with a final volume of 80 μL was prepared on ice from single-cell/nuclear suspensions that had passed the quality control using 0.4% trypan blue or AO/PI staining microscopic examination (viability, agglomeration rate, fragmentation rate, and nuclear integrity). It was injected into the SeekOne DD chip together with barcoding beads and carrier oil to generate water-in-oil droplets using a SeekOne DD kit. After droplet-based reverse transcription PCR, the droplets were subjected to demulsification, cDNA amplification and purification, fragmentation, end repair, adapter ligation, and library purification in sequence. The quality control requirements for the library stipulate that the main peak should be 350–750 bp and the concentration ≥1 ng/μL. Qualified libraries were sequenced on the Illumina platform (Hangzhou Cosmos Wisdom Biotech Co., Ltd). After parsing barcodes and unique molecular identifiers (UMIs) from the sequencing data, cDNA alignment and gene quantification were performed using SeekSoulTools to generate the gene expression matrix. Based on this matrix, cell quality control and filtering were performed using Scanpy as follows: (I) Cells with a mitochondrial gene transcript proportion exceeding 20% were removed to exclude dead or damaged cells. (II) Cells with total UMI counts below 500 were filtered out to remove low-quality droplets. (III) Cells with fewer than 500 or more than 7000 detected genes were excluded; the lower threshold eliminates cellular debris, while the upper threshold removes potential multiplets. (IV) Genes detected in at least 10 cells were retained to reduce low-frequency technical noise. Following total-count normalization, principal component analysis (PCA) was performed on highly variable genes. The top 15 principal components were used for UMAP dimensionality reduction and Louvain algorithm-based cell clustering. Cell clusters were annotated according to the expression of canonical lineage marker genes. Key markers included: T cells (Cd3d, Cd3e, Cd28), CD8⁺ T cells (Cd8a, Cd8b), CD4⁺ T cells (Cd4, Il7r, Ctla4), Th17 cells (Rorc, Il17a, Il17f, Ccr6), macrophages (Fcgr3, Adgre1, Itgam, Cd68, Cd163, Csf1r), dendritic cells (Itgax, Flt3, Batf, Thbd, Clec9a), neutrophils (Csf3r, Cxcr2, S100a8, S100a9), natural killer (NK) cells (Nkg7, Ccl5, Ncr1, Klrc1, Klrk1), epithelial cells (Epcam, Krt8, Cdh1), and fibroblasts (Col1a1, Pdgfra, Fap). Subsequently, based on the annotated clusters, differentially expressed genes (DEGs) were identified for each cluster using the Wilcoxon rank-sum test via the FindMarkers and FindAllMarkers functions in Seurat. DEGs were screened using thresholds of absolute log₂(fold change) >0.25 and adjusted *P*-value (FDR) ≤ 0.05. Gene Ontology (GO) functional annotation and Kyoto Encyclopedia of Genes and Genomes (KEGG) pathway enrichment analyses for the DEGs of each cluster were performed using the clusterProfiler package, with pathways considered significant at FDR < 0.05. Furthermore, CellChat was used to analyze the intercellular communication network, and Monocle2 was applied to construct pseudotemporal trajectories of cell differentiation.

### Metabolomics and data analysis

After grinding and disrupting the tissue in liquid nitrogen, 5 mg of the pulverized tissue was accurately weighed. Then, 400 μL of an 80% methanol solution containing an internal standard was added, and the tissue was homogenized by grinding. The homogenate was supplemented with an additional 600 μL of the same solution and vortex-mixed thoroughly. The mixture was centrifuged at 14,000 × *g* at 4 °C for 10 min, and the supernatant was transferred. Equal amounts of the supernatants were taken from each sample and mixed to form a pooled quality control (PQC) sample. After vacuum freeze-drying, the PQC was re-dissolved in 100 μL of 10% methanol-water solution. The solution was vortexed for 30 s, ultrasonicated for 1 min, and centrifuged at 14,000 × *g* at 4 °C for 10 min. The final supernatant was used for mass spectrometry analysis. Based on the Human Metabolome Database (HMDB) and the KEGG databases, functional annotations, classification, and pathway mapping were performed for all identified metabolites. The annotation results were visualized using the ggplot2 package. Based on the metabolite abundance data after logarithmic transformation, the univariate statistical analysis (*t*-test or Wilcoxon rank-sum test, *P* < 0.05) and the multivariate statistical model OPLS-DA [variable importance in projection (VIP) > 1] were used to screen for differentially expressed metabolites simultaneously satisfying the criteria *P* < 0.05 and VIP > 1. Further, the corto package was used to conduct metabolite set enrichment analysis (MSEA) on the differential metabolites. The metabolite-pathway regulatory network was constructed using the ggraph package to visualize the key metabolic pathways and their interactions.

### In vivo experiments

To establish a CRC mouse model, 2 × 10⁶ CT26 wild-type or stable CXCR4-knockdown CT26 (sh-CXCR4) cells were resuspended in 100 µL of PBS and subcutaneously inoculated into the right dorsal flank of 4–6-week-old male BALB/c mice (5 mice per group). Seven to ten days post-inoculation, when tumor volumes reached approximately 100 mm³, tumor-bearing mice were randomly assigned into four groups: Control, anti-PD-1 monotherapy (α-PD-1), CXCR4 knockdown (sh-CXCR4), and CXCR4 knockdown combined with α-PD-1 (sh-CXCR4 + α-PD-1). Mice in the α-PD-1 treatment group received intraperitoneal injections of 250 µg InVivoMAb anti-mouse PD-1 antibody (clone RMP1-14, BioXcell) every 3 days (in 100 µL PBS), for a total of 4 doses. During treatment, tumor length (L) and width (W) were measured every 3 days using calipers, and tumor volume was calculated using the formula: *V* (mm³) = (*L* × W²)/2. Meanwhile, mouse body weight was recorded daily to monitor toxicity, and general activity, food intake, and fur condition were observed. The humane endpoints predefined for this study included: >20% loss of initial body weight, severe mobility impairment, or tumor ulceration. No mice reached these endpoints during the experiment. Twenty-four hours after the final administration, all mice were euthanized, and tumor tissues were completely excised. The harvested tumor samples were either immediately processed for flow cytometry analysis or fixed/frozen for subsequent immunohistochemical and immunofluorescence analyses.

### Statistical analysis

Experimental data were analyzed using GraphPad Prism 8.0 and ImageJ software. Statistical significance was assessed using the Student’s t-test or analysis of variance (ANOVA). A *P* value less than 0.05 was considered statistically significant. All data are presented as mean ± standard deviation (*P < 0.05, **P < 0.01, ***P < 0.001, ****P < 0.0001).

## Results

### Construction of a CXCR4 knockdown mouse model

**Fig. 1:**
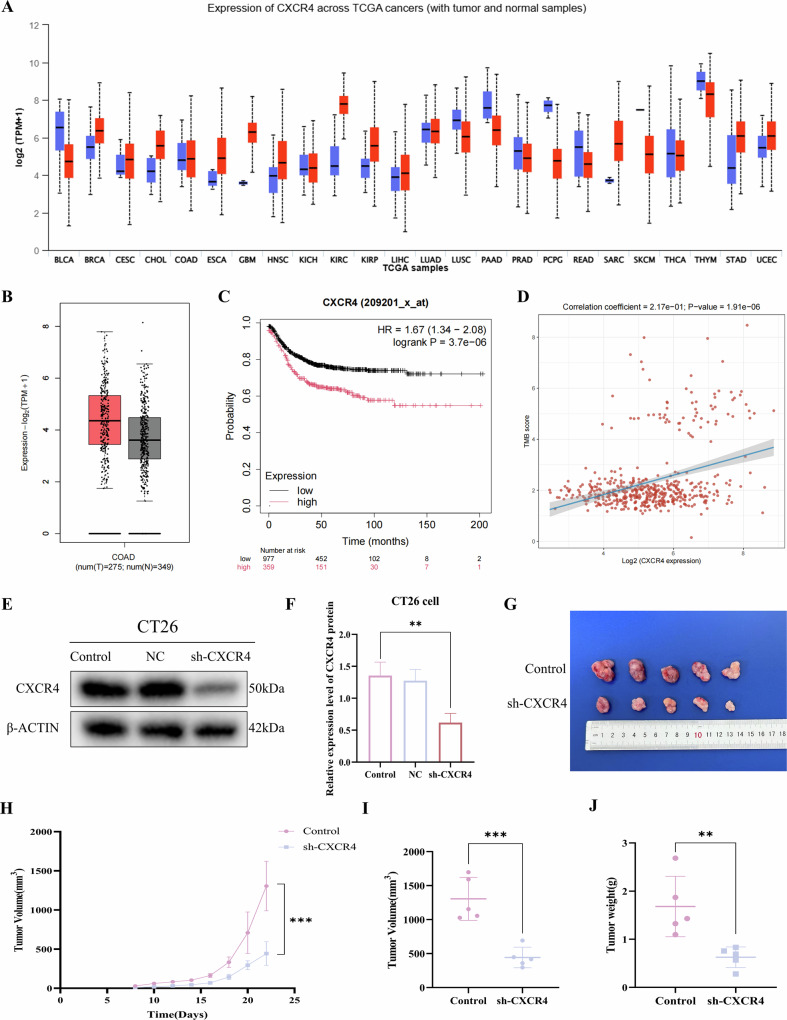
Construction of a CXCR4 knockdown mouse model. **A** UALCAN database was used to analyze CXCR4 expression levels in different tumors in the TCGA cohort. **B** Comparison of CXCR4 expression levels in CRC. **C** Survival curves of low and high CXCR4 expression in patients with CRC. **D** Relationship between CXCR4 expression and TMB score. **E**, **F** Western blot was used to detect the knockdown efficiency of interfering CXCR4 lentivirus in CT26 cells. **G** Images of tumor tissues from mice. **H** Growth curves of tumor volume over time in different groups. **I** Tumor volumes of mice in each group after dissection. **J** Tumor weights of mice in each group after dissection. *P < 0.05, **P < 0.01, ***P < 0.001, ****P < 0.0001.

Analysis of The Cancer Genome Atlas (TCGA) data using the UALCAN database revealed that CXCR4 expression in 24 tumor tissues was significantly higher than that in normal tissues (Fig. 1A) and also significantly higher in CRC tissues than in normal tissues (Fig. 1B). The relationship between CXCR4 expression and the prognosis of patients with CRC was evaluated using the Kaplan–Meier plotter. As shown in Fig. 1C, high expression of CXCR4 indicated a poor prognosis for patients with CRC. Further analysis revealed that the tumor mutational burden (TMB) in patients with CRC was significantly positively correlated with CXCR4 expression (Fig. 1D), suggesting that targeting CXCR4 may enhance the immunotherapy response.

We successfully constructed a mouse CRC CT26 cell line with stable knockdown of CXCR4 to clarify the role of CXCR4 in the malignant progression of CRC. Western blot analysis verified a significant reduction in CXCR4 protein expression (Fig. 1E, F). A mouse tumor-bearing model was established by subcutaneously injecting CRC cells, and the tumor volume and body weight were monitored. As shown in Fig. 1G–J, the tumor growth of mice was significantly inhibited in the sh-CXCR4 group compared with the control group, and both the tumor volume and weight of mice were significantly reduced. The tumor tissues were placed in tissue preservation solution or rapidly frozen in liquid nitrogen and stored at −80 °C for single-cell transcriptomics and metabolomics analyses.

### Regulation of the tumor immunosuppressive microenvironment by CXCR4 through interactions with TAMs and T cells

**Fig. 2:**
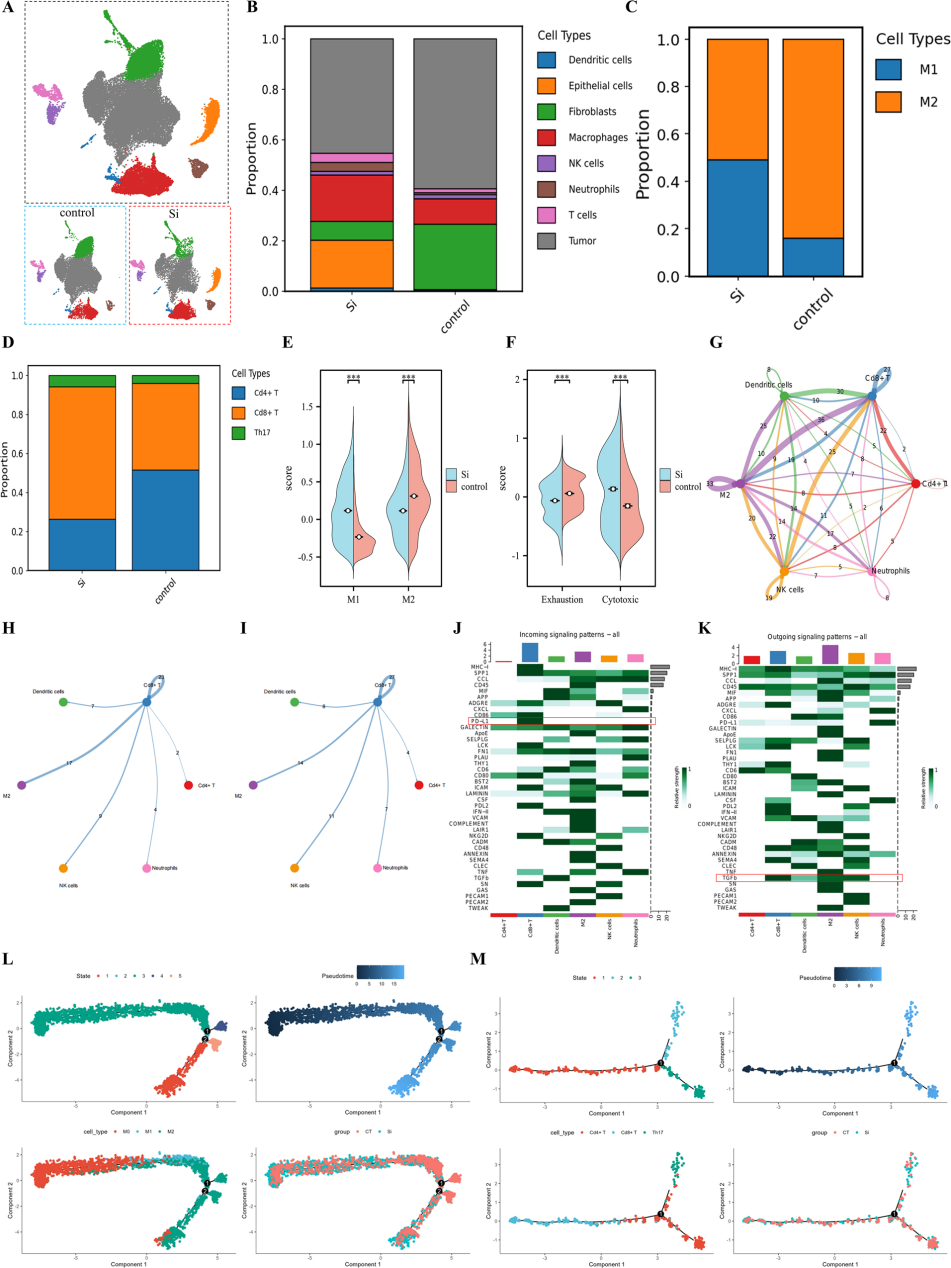
Single-cell transcriptomic analysis of mouse tumor tissues. **A** UMAP plots of 27,375 cells in the tumors of the two groups of mice. **B** Proportion of the main cell types in each group. **C** Proportion of macrophage subsets in each group. **D** Proportion of T cell subsets in each group. **E** Intergroup comparison of key marker expression levels in M1 and M2 macrophages. **F** Intergroup comparison of CD8⁺ T cell exhaustion and effector function marker expression levels. **G** Intercellular communication between immune cells. **H**, **I** Differences in the intensity of interaction between CD8^+^ T cells and other immune cells in each group. **J** Heat map of the incoming signal mode. **K** Heat map of the transmitted signal mode. **L** Relative proportions of macrophages in different trajectory branches of each group. **M** Relative proportions of T cells in different trajectory branches of each group. *P < 0.05, **P < 0.01, ***P < 0.001, ****P < 0.0001.

To investigate the mechanism underlying CXCR4-promoted tumor progression, tumor tissues were collected from the control and sh-CXCR4 (Si) groups (three mice per group) for scRNA-seq analysis. Samples from each group were pooled for library preparation. After quality control, 13,121 and 18,423 high-quality cells were retained from the control and Si groups, respectively. Dimensionality reduction, clustering, and visualization identified 13 distinct cell clusters, which were annotated as 8 major cell types (Fig. 2A). As shown in Fig. 2B, the TME composition of mice in the Si group was significantly different from that in the control group, manifested as an increase in the proportion of dendritic cells, macrophages, neutrophils, and T cells and a decrease in the proportion of fibroblasts. We conducted cluster analysis to further analyze the functional states of macrophages and T cells and obtained different functional subgroups. The results showed a significant increase in the proportions of M1-type TAMs and CD8^+^ T cells and a significant decrease in the proportions of M2-type TAMs and CD4^+^ T cells in the Si group (Fig. 2C, D). As shown in Fig. 2E, F, transcriptomic analysis revealed that CXCR4 knockdown drove macrophages toward a pro-inflammatory phenotype (upregulation of M1 genes such as NOS2 and IL1B) while suppressing their immunosuppressive program (downregulation of M2 genes including ARG1 and MRC1). Concurrently, it promoted the transition of CD8⁺ T cells from an exhausted state (downregulation of PDCD1 and CTLA4) to an effector state (upregulation of IFNG and GZMB). The CellChat analysis showed that CXCR4 knockdown significantly weakened the communication between M2-type TAMs and CD8^+^ T cells, while enhancing the interaction signals between CD8^+^ T cells and natural killer cells, neutrophils, CD4^+^ T cells, and dendritic cells (Fig. 2G–I). As shown in Fig. 2J, K, CD8^+^ T cells significantly received the inhibitory afferent signal of PD-L1. Meanwhile, M2-type TAMs and CD8^+^ T cells significantly dominated the signal transduction of the transforming growth factor-β (TGF-β) pathway. The pseudotime trajectory analysis showed that the differentiation trajectory of M0 macrophages to M2-type TAMs was significantly blocked in the Si group compared with the control group (Fig. 2L). In contrast, the differentiation pathway of T cells to CD8⁺ T cells was significantly enhanced (Fig. 2M). In summary, CXCR4 enhanced the TGF-β signal-mediated immunosuppressive function of M2-type TAMs and induced CD8⁺ T cell exhaustion, thereby driving the transformation of the tumor immune microenvironment into an immunosuppressive state.

### Promoting effect of CXCR4 on CRC progression through glutamine metabolic reprogramming

**Fig. 3:**
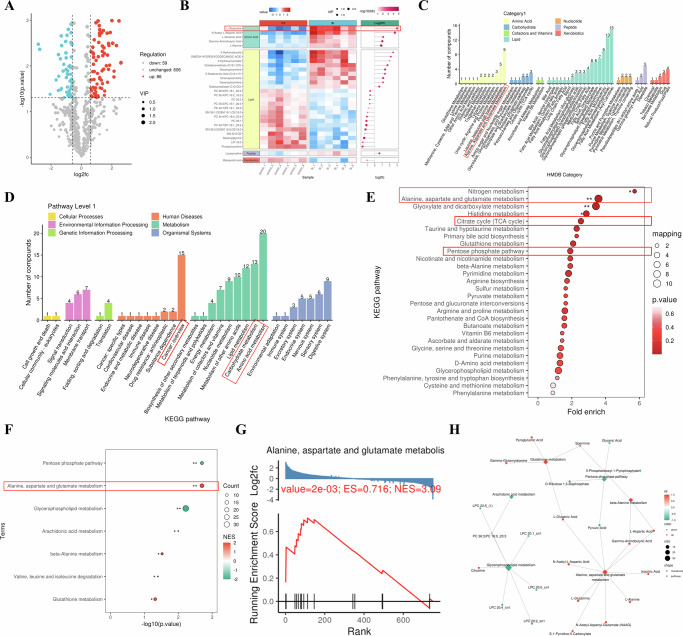
Nontarget metabolomics analysis of mouse tumor tissues. **A** Volcanic plot of differential metabolites. **B** Heat map of differential metabolites. **C** HMDB category bar chart of differential metabolites. **D** Bar chart of differential metabolites. **E** KEGG pathway enrichment analysis diagram. (**F**, **G**) MSEA enrichment analysis chart. **H** Metabolite-pathway grid diagram. *P < 0.05, **P < 0.01, ***P < 0.001, ****P < 0.0001.

The metabolomics analysis identified 135 differential metabolites. Among these, 86 metabolites were upregulated and 59 were downregulated in the sh-CXCR4 group (Fig. 3A). The heatmap of differential metabolites showed that amino acids and lipid metabolites were the main differential categories. Deep red represented a higher relative expression level, whereas dark blue represented a lower relative expression level. Among these, glutamine (L-glutamine) was the most crucial differential metabolite (Fig. 3B). The functional classification of differential metabolites indicated that amino acid metabolites were significantly enriched in the alanine, aspartate, and glutamate hub networks (Fig. 3C). As shown in Fig. 3D, the differential metabolites were mainly concentrated in amino acid metabolism, carbohydrate metabolism, and cancer. KEGG pathway enrichment analysis revealed significant enrichment in nitrogen metabolism, alanine, aspartate, and glutamate metabolism, the tricarboxylic acid (TCA) cycle, and pentose phosphate pathway (Fig. 3E). The MSEA results showed that the metabolites were significantly enriched in alanine, aspartate, and glutamate metabolism (Fig. 3F, G). The results of the pathway network analysis showed glutamine and glutamate metabolism as the central hub pathways (Fig. 3H). In summary, the metabolomics analyses collectively indicate that CXCR4 drives a metabolic reprogramming centered on the glutamine/glutamate axis. The central role of this pathway suggests that CXCR4 likely promotes the malignant progression of CRC by modulating this key metabolic hub.

### Influence of CXCR4 on the metabolic reprogramming of CRC cells through Akt/SMAD4

**Fig. 4:**
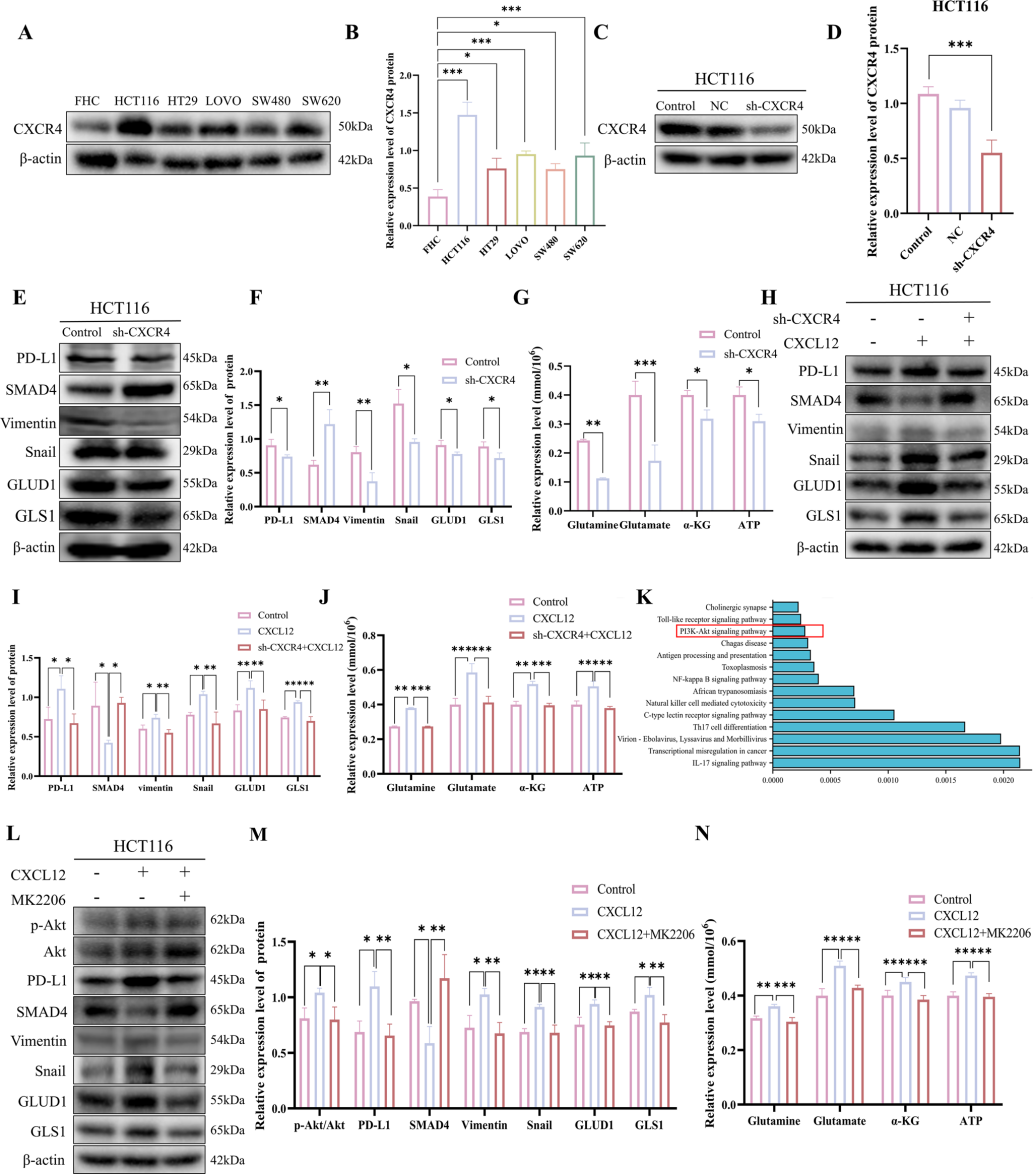
CXCR4 influenced the metabolic reprogramming of CRC cells through the PI3K-Akt pathway. **A**, **B** Western blot was used to detect CXCR4 expression in CRC cell lines. **C**, **D** Western blot was used to detect the knockdown efficiency of interfering CXCR4 lentivirus in HCT116 cells. **E**, **F** Western blot was used to detect the expression of PD-L1, SMAD4, vimentin, Snail, GLUD1, and GLS1 after CXCR4 knockdown. **G** Changes in glutamine, glutamate, α-KG, and ATP levels. **H**, **I** Western blot was used to detect the expression of PD-L1, SMAD4, vimentin, Snail, GLUD1, and GLS1 after CXCL12 stimulation and CXCR4 knockdown. **J** Detection of glutamine, glutamate, α-KG, and ATP levels. **K** KEGG pathway enrichment analysis diagram. **L**, **M** Western blot was used to detect the expression of p-Akt/Akt, PD-L1, SMAD4, vimentin, Snail, GLUD1, and GLS1 after CXCL12 stimulation and MK2206 treatment. **N** Changes in glutamine, glutamate, α-KG, and ATP levels after different treatments. *P < 0.05, **P < 0.01, ***P < 0.001, ****P < 0.0001.

We first verified the expression level of CXCR4 in the cell line. Western blot results showed that CXCR4 expression was upregulated in CRC cells (HCT116, HT29, LOVO, SW480, and SW620) compared with normal FHC cells. The expression was significantly upregulated in HCT116 cells (Fig. 4A, B). We constructed CXCR4-stable knockdown HCT116 cells and verified their knockdown efficiency using Western blot (Fig. 4C, D). To investigate the specific mechanism by which CXCR4 regulates glutamine metabolism, we conducted Western blot and metabolite detection. The expression of PD-L1, key enzymes of glutamine metabolism GLS1 and GLUD1, and epithelial-mesenchymal transition (EMT) marker proteins vimentin and Snail was downregulated, whereas the expression of SMAD4 was upregulated in the sh-CXCR4 group compared with the control group (Fig. 4E, F). Further metabolite detection revealed that CXCR4 knockdown led to a significant decrease in the concentrations of glutamine, glutamate, α-ketoglutarate (α-KG), and ATP within cells (Fig. 4G). It indicated that knocking down CXCR4 significantly inhibited glutamine metabolism and energy production. Western blot analysis indicated that CXCL12 stimulation significantly upregulated the expression of PD-L1, GLS1, GLUD1, vimentin, and Snail and downregulated the expression of SMAD4 compared with that in the control group; however, CXCR4 knockdown reversed this phenomenon (Fig. 4H, I). The levels of glutamine, glutamate, α-KG, and ATP in the cells increased in the CXCL12 stimulation group compared with the control group. Similarly, knocking down CXCR4 could reverse these changes (Fig. 4J). We conducted a KEGG enrichment analysis on CXCR4-related genes in tumor cells from our scRNA-seq data to explore how the CXCL12/CXCR4 axis influenced glutamine metabolism, revealing that this gene set was significantly enriched in the PI3K-Akt pathway (Fig. 4K). The Western blot results showed that the expression of p-Akt/Akt, PD-L1, key enzymes of glutamine metabolism (GLS1 and GLUD1), and EMT marker proteins (vimentin and Snail) were upregulated, whereas the expression of SMAD4 was downregulated in the CXCL12 stimulation group compared with the control group. However, the combination group with PI3K-Akt pathway inhibitor (MK2206) reversed these effects (Fig. 4L, M). The metabolite detection results consistently indicated that MK2206 treatment blocked the boosting effect of CXCL12 on glutamine, glutamate, α-KG, and ATP levels (Fig. 4N). The aforementioned experiments indicated that CXCR4 inhibited SMAD4 expression by activating the PI3K-Akt pathway, thereby influencing the metabolic reprogramming and EMT process of CRC cells.

### SMAD4 deletion drives glutamine metabolic reprogramming in CRC cells via the CXCL12/CXCR4 signaling axis

**Fig. 5:**
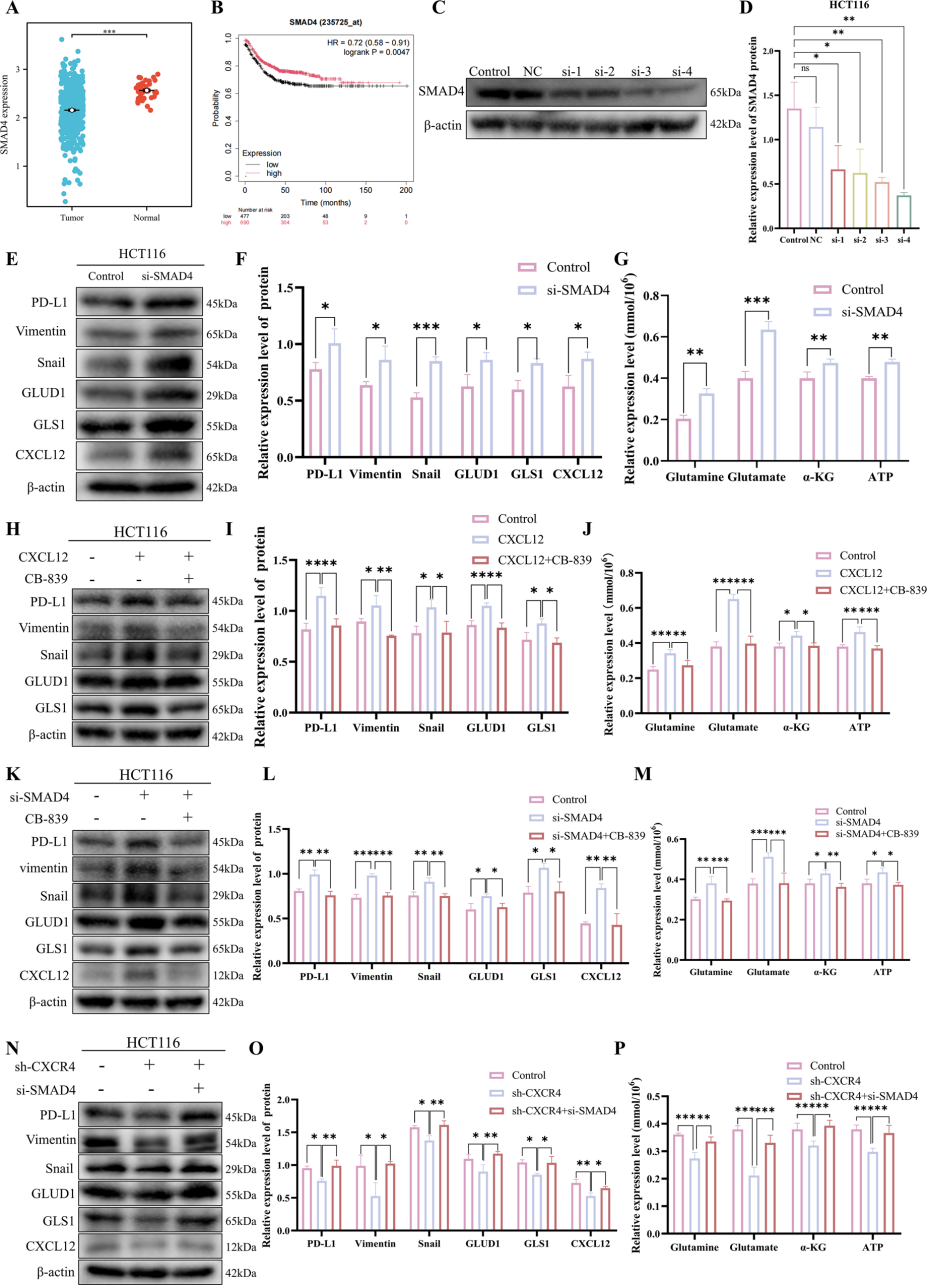
SMAD4 deletion drove glutamine metabolism dependence in CRC cells through CXCL12 upregulation. **A** Expression of SMAD4 in CRC. **B** Prognosis of CXCR4 was analyzed using the Kaplan-Meier plotter database. **C**, **D** Western blot analysis interfered with the knockdown efficiency of SMAD4. **E**, **F** Western blot was used to detect the expression of PD-L1, vimentin, Snail, GLUD1, GLS1, and CXCL12 after interference with SMAD4. **G** Changes in glutamine, glutamate, α-KG, and ATP levels. **H**, **I** Western blot was used to detect the expression of PD-L1, vimentin, Snail, GLUD1, and GLS1 after CXCL12 stimulation and CB-839 treatment. **J** Changes in glutamine, glutamate, α-KG, and ATP levels after different treatments. **K**, **L** Western blot was used to detect the expression of PD-L1, vimentin, Snail, GLUD1, GLS1, and CXCL12 after different treatments. **M** Detection of glutamine, glutamate, α-KG, and ATP levels. **N**, **O** Western blot was used to detect the expression of PD-L1, vimentin, Snail, GLUD1, GLS1, and CXCL12 after CXCR4 knockdown and interference with SMAD4. **P** Changes in glutamine, glutamate, α-KG, and ATP levels. *P < 0.05, **P < 0.01, ***P < 0.001, ****P < 0.0001.

SMAD4 deletion is present in approximately 30% of patients with CRC, and is closely related to enhanced tumor invasiveness, increased metastatic ability, a tendency to occur in specific sites, and a poor prognosis [19]. It affects tumor cell metabolism and induces immunosuppressive states by mediating changes in chemokine expression, thereby promoting the formation of an immunosuppressive microenvironment [20]. As shown in Fig. 5A, SMAD4 expression is low in CRC. The Kaplan-Meier plotter showed that the low expression of SMAD4 was associated with a reduced overall survival rate in patients with poor prognosis (Fig. 5B). We constructed multiple interference fragments targeting SMAD4 (si-SMAD4) and transfected them into CRC cells HCT116. The Western blot results showed that the interference effect of si-4 was the most significant (Fig. 5C, D). Therefore, we chose si-4 for subsequent experiments. The Western blot results indicated that si-SMAD4 could significantly promote the expression of PD-L1, key enzymes of glutamine metabolism GLS1 and GLUD1, EMT marker proteins vimentin and Snail, and CXCL12 compared with that in the control group (Fig. 5E, F). The levels of glutamine, glutamate, α-KG, and ATP increased in the si-SMAD4 group compared with the control group (Fig. 5G). We explored and verified the functional connection between CXCR4, SMAD4, and glutamine metabolism in colon cancer cells by CXCL12 stimulation and SMAD4 knockdown in combination with the glutaminase inhibitor CB-839. The Western blot results showed that the expression level of PD-L1, key enzymes of glutamine metabolism GLS1 and GLUD1, and EMT marker proteins vimentin and Snail were upregulated in the CXCL12 stimulation group compared with the control group. The combination group with CB-839 could reverse these effects (Fig. 5H, I). Similarly, the levels of glutamine, glutamate, α-KG, and ATP were increased in the CXCL12 stimulation group compared with the control group and CB-839 could reverse these phenomena (Fig. 5J). The results of Western blot and metabolite detection indicated that CB-839 effectively reversed the upregulation of PD-L1, GLS1, GLUD1, vimentin, Snail, and CXCL12 protein expression caused by SMAD4 knockdown (Fig. 5K, L) and the increase in glutamine, glutamate, α-KG, and ATP levels (Fig. 5M). These results suggested a key role of the glutamine metabolic pathway in the effects mediated by SMAD4 deletion. Next, we investigated the role of CXCR4, the receptor of CXCL12, in the SMAD4 deletion regulatory pathway. As shown in Fig. 5N, O, CXCR4 knockdown significantly downregulated the expression of PD-L1, GLS1, GLUD1, vimentin, Snail, and CXCL12 compared with the control group, whereas co-transfection with si-SMAD4 reversed these changes. The levels of glutamine, glutamate, α-KG, and ATP were decreased in the sh-CXCR4 group compared with the control group, and these phenomena were reversed in the si-SMAD4 group (Fig. 5P). In conclusion, the CXCL12/CXCR4 axis may drive glutamine metabolism reprogramming by inhibiting SMAD4 expression, thereby promoting PD-L1-mediated immunosuppression and ultimately accelerating CRC progression.

### CXCR4 promotes CRC metastasis by inducing macrophage M2 polarization and CD8^+^ T cell exhaustion

**Fig. 6:**
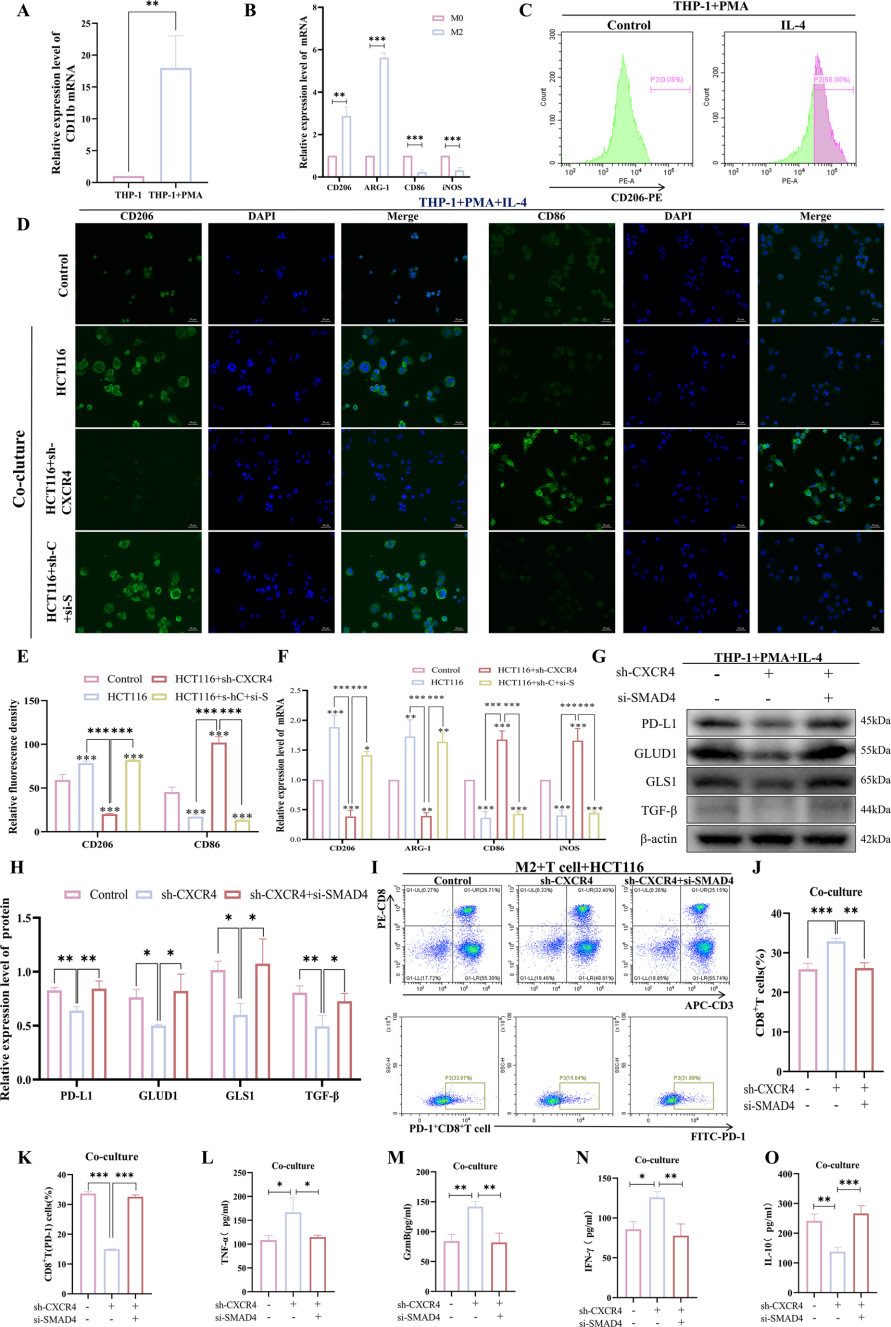
CXCR4 reshaped the macrophage phenotype in the TME and promoted CD8^+^ T cell exhaustion. **A** Expression of mRNA in THP-1 cells treated with PMA (100 ng/mL) was detected using qRT-PCR. **B** qRT-PCR was used to detect the mRNA expression in M0-type macrophages treated with IL-4 (20 ng/mL). **C** Flow cytometry was used to detect the proportion of M2-type macrophages. **D**, **E** Immunofluorescence was used to detect the expression of CD206 and CD86 in M2-type macrophages co-cultured with HCT116 cells under different treatments. **F** qRT-PCR was used to detect the expression of CD206, ARG-1, CD86, and iNOS in M2-type macrophages co-cultured with HCT116 cells under different treatments. **G**, **H** Western blot was used to detect the expression of PD-L1, GLS1, GLUD1, and TGF-β in M2-type macrophages co-cultured with HCT116 cells under different treatments. **I**–**K** Flow cytometry analysis of the proportion of CD8⁺ T cells and PD-1 expression level after co-culturing T cells with supernatants from different macrophage groups. **L**–**O** ELISA was used to detect the contents of TNF-α, GzmB, IFN-γ and IL-10 in the supernatant of T cells co-cultured with the supernatants from different macrophage culture groups. *P < 0.05, **P < 0.01, ***P < 0.001, ****P < 0.0001.

scRNA-seq revealed that CXCR4 promoted the formation of an immunosuppressive microenvironment by inducing M2 polarization of macrophages and CD8^+^ T cell exhaustion. This study aimed to elucidate the specific mechanisms through which CXCR4 drives malignant progression and immune suppression. We used a co-culture system of CRC cells and immune cells to explore whether CXCR4 regulated macrophage polarization and induced CD8^+^ T cell exhaustion. We induced macrophages to an immunosuppressive phenotype using PMA and an anti-inflammatory cytokine IL-4, and then co-cultured them with CRC cells. The qRT-PCR results showed that PMA treatment significantly increased the expression of CD11b (Fig. 6A), confirming the successful differentiation of THP-1 cells into M0-type macrophages. IL-4 treatment significantly upregulated the expression of CD206 and ARG-1 and downregulated the expression of CD86 and iNOS (Fig. 6B), indicating that IL-4 successfully induced the polarization of M0-type macrophages to M2-type macrophages. Also, flow cytometry confirmed that the proportion of M2-type macrophages in the IL-4 group was as high as 68% (Fig. 6C). We inoculated THP-1 cells into the lower chamber of Transwell and induced them to differentiate into M2-type macrophages after co-stimulation with PMA and IL-4 for 48 h. Then, HCT116 cells that had undergone different treatments (e.g., transfection with sh-CXCR4, si-SMAD4, etc.) were inoculated into the upper chamber for co-culture and subjected to different treatments. The lower-chamber macrophages were collected for immunofluorescence, qRT-PCR, and Western blot analysis. Immunofluorescence analysis revealed that, compared with the HCT116 group, the HCT116+sh-CXCR4 group exhibited decreased expression of the M2 marker CD206 but increased expression of the M1 marker CD86. Importantly, this phenotypic shift could be rescued by transfection with si-SMAD4 (HCT116+sh-C+si-S) (Fig. 6D, E). As shown in Fig. 6F, the expression of CD206 and ARG-1 was significantly reduced. In contrast, the expression of CD86 and iNOS increased in the HCT116 + sh-CXCR4 group compared with the HCT116 group. Transfection with si-SMAD4 could reverse these phenomena. Western blot analysis showed that the expression of PD-L1, GLS1, GLUD1, and TGF-β was significantly downregulated in macrophages within the CXCR4 knockdown co-culture group compared with the control group; the transfection with si-SMAD4 could reverse these changes (Fig. 6G, H). To further assess the impact on T cell function, we co-cultured T cells by mixing the supernatant of HCT116 cells with different treatments co-cultured with M2-type TAM with complete culture medium. Figure 6I–K shows an increase in the proportion of CD8⁺ T cells along with a decrease in PD-1 expression in the sh-CXCR4 group versus control, effects that were reversed by the si-SMAD4 combination. ELISA results showed that the secretion of TNF-α, GzmB, and IFN-γ significantly increased in the supernatant and the secretion of IL-10 significantly decreased in the sh-CXCR4 group compared with the control group. Transfection of si-SMAD4 could reverse these changes (Fig. 6L–O). These results indicated that CXCR4 in CRC shaped an immunosuppressive microenvironment by promoting M2 polarization of macrophages and inducing CD8⁺ T cell exhaustion, thereby driving tumor progression.

### CXCR4 reprograms glutamine metabolism in macrophages via the PI3K-Akt pathway to promote M2 polarization

**Fig. 7:**
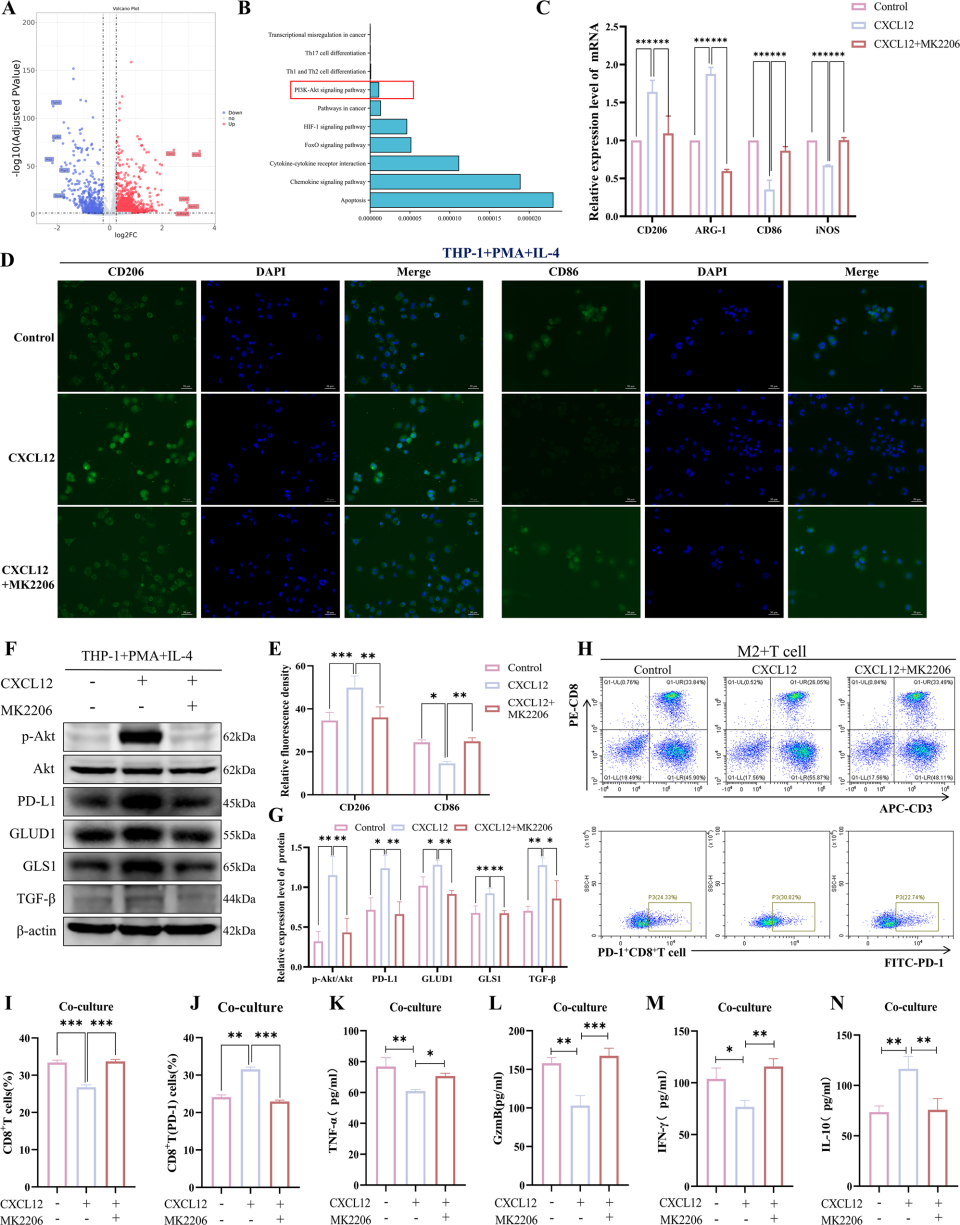
CXCR4 promoted M2 polarization in macrophages by reprogramming glutamine metabolism. **A** Volcano plot of differential genes in macrophages. **B** Enrichment analysis of the KEGG pathway for differential genes in macrophages. **C** qRT-PCR was used to detect the mRNA expression in M2-type macrophages treated differently. **D**, **E** Immunofluorescence was used to detect the expression of CD206 and CD86 in M2-type macrophages treated differently. **F**, **G** Western blot was used to detect the expression of p-Akt/Akt, PD-L1, GLS1, GLUD1, and TGF-β in M2-type macrophages treated differently. **H**–**J** Flow cytometric analysis of the proportion of CD8⁺ T cells and PD-1 expression in T cells co-cultured with differentially treated macrophages. **K**–**N** ELISA was used to detect the levels of TNF-α, GzmB, IFN-γ, and IL-10 in the supernatants of T cells and macrophages under different treatments. *P < 0.05, **P < 0.01, ***P < 0.001, ****P < 0.0001.

We conducted KEGG enrichment analysis on differentially expressed genes in macrophages based on scRNA-seq to clarify the mechanism by which CXCR4 influenced M2 polarization in macrophages. The results showed significant enrichment of the PI3K-Akt pathway (Fig. 7A, B). The qRT-PCR results showed CXCL12 stimulation significantly upregulated the expression of CD206 and ARG-1 and downregulated the expression of CD86 and iNOS compared with that in the control group. MK2206 treatment could reverse these changes (Fig. 7C). Immunofluorescence analysis revealed that CXCL12 stimulation increased CD206 and decreased CD86 expression compared to control, effects that were reversed upon co-treatment with MK2206 (Fig. 7D, E). These results confirmed that CXCR4 drove the polarization of macrophages to M2 type by activating the PI3K-Akt pathway. Mechanistically, Western blot analysis revealed that CXCL12 stimulation significantly upregulated the expression of p-Akt/Akt, the immune checkpoint molecule PD-L1, key enzymes of glutamine metabolism GLS1 and GLUD1, and TGF-β. These effects were also reversed by MK2206 (Fig. 7F, G). Further functional experiments demonstrated that conditioned medium from CXCL12-stimulated macrophages reduced the proportion of co-cultured CD8⁺ T cells and increased the expression of the exhaustion marker PD-1 (Fig. 7H–J). It also reduced the secretion of TNF-α, GzmB, and IFN-γ while increasing IL-10 secretion (Fig. 7K–N). MK2206 treatment reversed these immunosuppressive effects of the macrophage conditioned medium on T cells. These experiments showed that CXCR4 mediated the reprogramming of glutamine metabolism in macrophages by activating the PI3K-Akt signaling pathway and driving their polarization toward the M2 type, thereby inhibiting the function of CD8⁺ T cells, jointly shaping the immunosuppressive TME.

### Enhancement of the therapeutic effect of α-PD-1 immunotherapy by targeted inhibition of CXCR4 expression

**Fig. 8:**
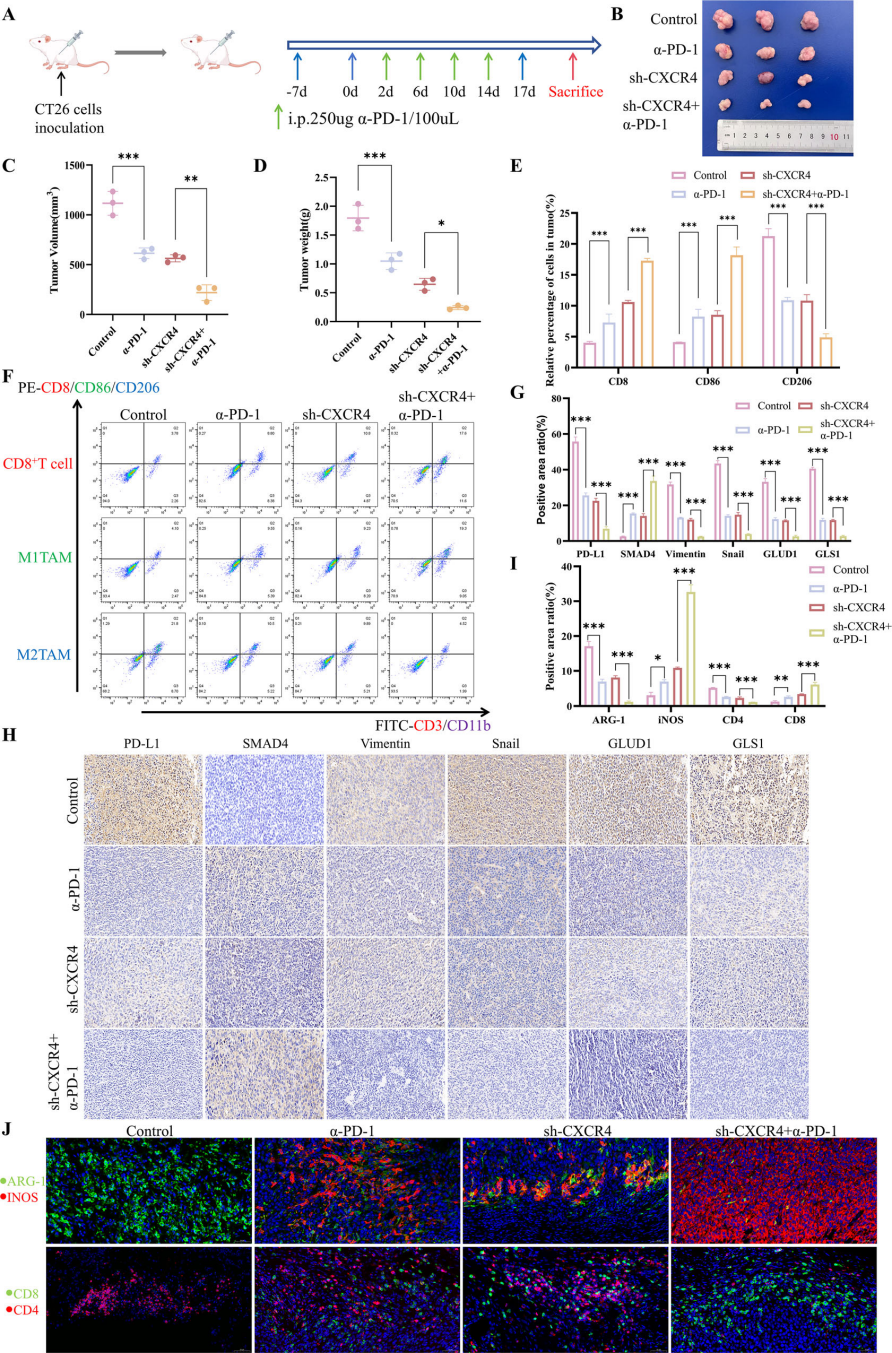
Targeted inhibition of CXCR4 synergistically enhanced the antitumor efficacy of α-PD-1 immunotherapy in the CT26 mouse CRC model. **A** Flowchart of α-PD-1 for in vivo treatment of xenograft tumors. **B** Images of the dissected tumors in each group. **C** Volume of the dissected tumors in each group. **D** Weight of the dissected tumors in each group. **E**, **F** Flow cytometry was used to detect the proportions of CD8^+^ T cells, M1 TAMs, and M2 TAMs in each group of tumors. **G**, **H** Staining results of PD-L1, GLS1, GLUD1, vimentin, Snail, and SMAD4 in tumor sections under different treatments (scale = 50 μm). **I**, **J** Immunofluorescence was used to detect the expression in M1 (iNOS), M2 (ARG-1) cells, CD8^+^ T cells (CD8), and CD4^+^ T cells (CD4) in tumors (scale = 50 μm). *P < 0.05, **P < 0.01, ***P < 0.001, ****P < 0.0001.

**Fig. 9:**
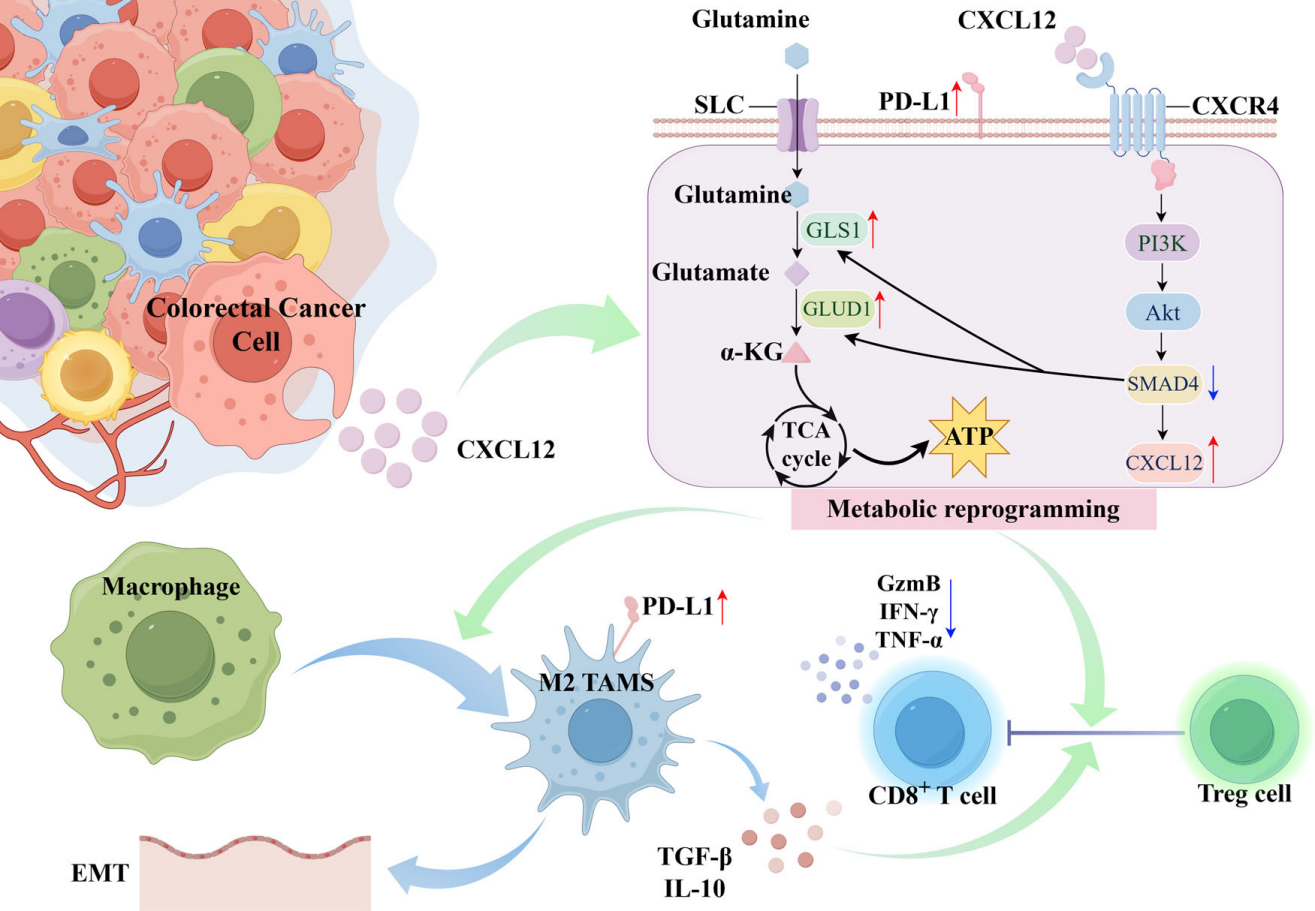
Schematic model of CXCR4-driven immune escape via the metabolic-immune axis in CRC.

M2 TAMs shape the immunosuppressive microenvironment through direct or indirect interactions with various cells in the TME, leading to immune evasion and immunotherapy tolerance [21]. Given that CXCR4 can promote the infiltration of M2-type TAMs in tumors and induce CD8⁺ T cell exhaustion, we employed a CXCR4 blockade strategy to evaluate whether targeting CXCR4 could reverse such immunosuppressive effects and enhance the efficacy of α-PD-1 therapy, aiming to improve the effectiveness of immunotherapy. As shown in Fig. 8A, we established a tumor-bearing model of CT26 CRC in mice and intraperitoneally injected PBS or α-PD-1 every 3 days, recording tumor volume and body weight changes. When the tumor reached the predetermined size, the mouse was euthanized and the tumor tissue was dissected and photographed (Fig. 8B). As shown in Fig. 8C, D, the tumor growth was significantly inhibited in the sh-CXCR4+α-PD-1 group. We prepared single-cell suspensions from tumor tissues. The flow cytometry results showed a significant increase in the infiltration of CD8^+^ T cells and M1 TAMs and a decrease in the infiltration of M2 TAMs (Fig. 8E, F). The immunohistochemical results showed that the expression of PD-L1, key enzymes of glutamine metabolism GLS1 and GLUD1, and EMT marker proteins vimentin and Snail was downregulated and that of SMAD4 was upregulated in the sh-CXCR4+α-PD-1 group (Fig. 8G, H), which was consistent with the findings in vitro. The immunofluorescence results showed that sh-CXCR4+α-PD-1 treatment reduced the expression of M2 TAM marker (Arg-1) but enhanced the expression of M1-type marker (iNOS) and CD8⁺ T cell infiltration (Fig. 8I, J). In conclusion, these data indicated that the targeted inhibition of CXCR4 enhanced the response of CRC to immunotherapy by reshaping the tumor immune microenvironment, promoting the activation and infiltration of CD8⁺ T cells, and reducing the proportion of M2-type TAMs, thereby providing a clinical basis for the development of related combination treatment strategies (Fig. 9).

## Discussion

The TME has a significant impact on the malignant progression, treatment response, and clinical prognosis of CRC [22]. Cancer cells have evolved several strategies to evade the immune system, such as reducing the expression of tumor-associated antigens, enhancing the production of inhibitory molecules, and creating an environment suppressing immune responses [23]. Although ICIs have shown significant efficacy in a variety of malignancies [24]. their therapeutic benefits remain limited for most patients with pMMR/MSS tumors [25]. The CXCL12/CXCR4 signaling axis promotes the proliferation, metastasis, and angiogenesis of tumor cells and the formation of an immunosuppressive TME [11]. For instance, TGF-β1 upregulates SOX18 expression in a Smad2/3-dependent manner, driving the recruitment of TAMs and Tregs mediated by the CXCL12-CXCR4 axis, creating an immune tolerance microenvironment, and thereby promoting the immune escape and metastasis of HCC [26]. Our Single-cell transcriptomics analysis in this study revealed the mechanism by which CXCR4 promoted the infiltration of M2-type TAMs and induced CD8⁺ T cell exhaustion, creating an immunosuppressive microenvironment and thereby driving the malignant progression of CRC. This discovery establishes CXCR4 as a promising therapeutic target and provides a key mechanistic rationale for the “CXCR4 inhibitor plus α-PD-1” combination therapy. This strategy aims to remodel the TME by targeting CXCR4, which is expected to overcome immunotherapy resistance in pMMR/MSS CRC, thereby opening a new translational avenue to expand the potential beneficiary population.

CXCR4 is a widely expressed GPCR. Its abnormal expression promotes tumor proliferation, chemotherapy resistance, and distant metastasis by constitutively activating signaling pathways such as MAPK, PI3K/Akt, and PLC [27]. Meanwhile, the high expression of CXCL12 secreted by stromal cells in the TME induces PD-L1 overexpression in tumor cells and promotes the infiltration of immunosuppressive cells, collectively shaping the immunosuppressive microenvironment [28]. This study found that CXCR4 inhibits the expression of SMAD4 through the PI3K-Akt signaling pathway. SMAD4 is a key transcription factor in the TGF-β signaling pathway [29]. and its functional loss is closely associated with immune escape. For example, in gastric cancer (GC), SMAD4 deficiency leads to increased secretion of CXCL1. This, in turn, impairs the dendritic cell/T cell axis and promotes the accumulation of granulocytic myeloid-derived suppressor cells (G-MDSCs), thereby inducing immune escape and conferring resistance to ICB monotherapy [30]. Furthermore, downregulation of SMAD4 is significantly associated with elevated expression of CXCL12 [31]. CXCL12 can induce M2 polarization in macrophages and upregulate PD-L1 expression. Meanwhile, M2-type macrophages secrete immunosuppressive factors such as TGF-β and epidermal growth factor (EGF), which synergistically promote tumor progression and immune escape [32]. Notably, deficiency of SMAD4 also drives cellular metabolic reprogramming [33]. This study further revealed that CXCR4 regulates SMAD4 expression through the PI3K-Akt pathway. The absence of SMAD4 leads to dysregulated expression of key metabolic enzymes GLS1 and GLUD1 in metabolic pathways and alters the levels of glutamine, glutamate, α-KG, and ATP in cells, thereby driving tumor metabolic reprogramming. The deficiency of glutamine in the TME can inhibit the proliferation of CD8⁺ T cells and the production of cytokines, while promoting the expansion of Tregs [34]. In addition, SMAD4 promotes distant metastasis of tumor cells by regulating the expression of genes related to EMT [20]. In summary, this study reveals that CXCR4, by inhibiting SMAD4, functions as a central hub that coordinately regulates immunosuppression, metabolic reprogramming, and tumor metastasis, thereby driving malignant progression. Based on this pivotal role, targeting CXCR4 holds promise for simultaneously remodeling the immune and metabolic microenvironment, providing a novel rationale for combination therapies that achieve dual targeting of both metabolism and immunity.

Glutamine plays a key role in tumor progression and invasiveness [35]. The scRNA-seq and metabolomic analysis revealed that CXCR4 drove the formation of an immunosuppressive microenvironment by regulating glutamine metabolism reprogramming in the TME. Glutamine enters cells through solute carrier (SLC)-type transporters and is catabolized into glutamate and ammonia by glutaminase. Glutamate is converted into α-KG under the action of glutamate dehydrogenase, which supports the TCA cycle and provides the biosynthetic precursor required for the growth and proliferation of cancer cells [13]. We found that CXCR4 knockdown significantly downregulated the expression of GLS1, GLUD1, and PD-L1 and reduced the levels of glutamine, glutamate, α-KG, and ATP. Enhanced glutamine metabolism in the TME drives M2 polarization in macrophages by upregulating GLS1 expression and mediates trastuzumab resistance in HER2-positive GC [36]. The CXCL12/CXCR4 axis activates the PI3K-Akt pathway, upregulates the expression of GLS1, GLUD1, PD-L1, and TGF-β in macrophages, and induces M2 polarization. Furthermore, the supernatant co-culture experiments further demonstrated that M2 macrophages stimulated by CXCL12 promoted the exhaustion of CD8⁺ T cells, inhibited the secretion of TNF-α, GzmB, and IFN-γ, and increased the secretion of IL-10, thereby collectively driving the formation of the immunosuppressive microenvironment.

GLS1 is a key enzyme in glutamine metabolism. Its upregulation is often accompanied by increased glutamine uptake and dependence in various cancers, and high expression levels are significantly associated with poor prognosis [37]. The GLS1 inhibitor CB-839 is currently undergoing Phase I/II clinical trials in multiple cancers [38]. One study has shown that the combined treatment of CB-839 and nivolumab can benefit patients who are ineffective or have progressed to ICI treatment [39]. CB-839 effectively enhanced the antitumor activity of T-cell-mediated immunotherapy in melanoma models by increasing the number of circulating Pmel-1 T cells and promoting their antigen activation [40]. We found that CB-839 effectively inhibited the upregulation of GLS1, GLUD1, and PD-L1 expression caused by CXCL12 stimulation or SMAD4 knockdown, and also inhibited the increase in glutamine, glutamate, α-KG, and ATP levels. Furthermore, CB-839 significantly inhibited CXCL12 secretion and EMT in CRC cells. Collectively, these results indicate that targeting glutamine metabolism downstream of CXCR4 effectively reverses associated immunosuppression, providing direct experimental support for the proposed combination therapy.

Blocking the recruitment of immunosuppressive cells for sensitizing ICI treatment is a promising combined strategy [26]. The IL-6/JAK/STAT3 pathway promotes the recruitment of MDSCs and drives immune escape. Blocking IL-6 can inhibit the recruitment of MDSCs and thereby enhance the efficacy of ICIs [41]. Leukemia inhibitory factor (LIF) induced immunosuppressive TME enriched with M2 TAMs. The combination of anti-LIF, anti-PD-L1, and local stereotactic radiotherapy (SBRT) demonstrated potential as a neoadjuvant treatment strategy [42]. Among these targets, CXCR4 stands out for its pivotal role in recruiting immunosuppressive cells. Antagonists such as Plerixafor and Balixafortide can potentiate the efficacy of ICIs by blocking the recruitment of Tregs and M2-type TAMs [43, 44]. This study demonstrated that CXCR4 knockdown inhibited tumor growth, EMT, and glutamine metabolism both in vivo and in vitro, impeded macrophage M2 polarization, and promoted CD8⁺ T cell infiltration into tumors. In vivo, CXCR4 targeting significantly enhanced the efficacy of α-PD-1 therapy; the combination effectively suppressed CRC progression and metastasis.

In summary, this study demonstrates that CXCR4 downregulates SMAD4 via the PI3K-Akt axis, thereby relieving SMAD4-mediated suppression of CXCL12 and PD-L1. This cascade ultimately promotes M2 TAMs infiltration, suppresses CD8⁺ T cell cytotoxicity, and inhibits M1 TAM polarization to foster an immunosuppressive microenvironment. Within this milieu, the infiltrating M2 TAMs produce TGF-β, which further upregulates CXCR4 expression, establishing a positive feedback loop. Moreover, CXCR4 drives glutamine metabolic reprogramming in both tumor and immune cells, providing metabolic support for M2 polarization and immune evasion and thus synergistically reinforcing immunosuppression. Consequently, combining CXCR4 targeting with α-PD-1 therapy effectively remodels the immune microenvironment, inhibits tumor progression and metastasis, and provides a rationale for novel combination strategies in CRC.

## Conclusions

This study found that CXCR4-mediated glutamine metabolism reprogramming by activating the PI3K/Akt/SMAD4 pathway. This axis drives M2-type TAMs polarization and CD8⁺ T cell exhaustion, collectively shape the immunosuppressive microenvironment and promoted ICI resistance and tumor progression. The targeted inhibition of CXCR4 suppressed the polarization of M2-type TAMs and increased the infiltration of CD8⁺ T cells, thereby reversing immunosuppression and enhancing the efficacy of α-PD-1. We integrated scRNA-seq with metabolomics and clarified the vital role of CXCR4 in the immune metabolism in CRC, thus providing a theoretical basis for treating CRC with CXCR4 inhibitors combined with PD-1/PD-L1 blockade.

## Supplementary information

Supplementary Text Description

Supplementary Figure 1

Supplementary Figure 2

Supplementary Figure 3

Supplementary Figure 4

Supplementary Figure 5

Supplementary Western Blot

## Data Availability

The raw sequencing data supporting the findings of this study have been deposited in the Genome Sequence Archive (GSA) under accession code CRA038349. All other data are available in the main text or the supplementary materials.
