# Supplementary material for: Multi-omics reveals CXCR4 drives immune escape in colorectal cancer via metabolic reprogramming and immune microenvironment remodeling
Source: Cell Death Dis. 2026 May 4;17(1):591. doi: 10.1038/s41419-026-08795-x (PMC13287491; doi:10.1038/s41419-026-08795-x)
Supplement: Supplementary file 1 — Supplementary Text Description [file 41419_2026_8795_MOESM1_ESM.docx]

1. ****Targeting CXCR4 Combined with Anti-PD-1 Synergistically Reshapes the Immune Microenvironment and Inhibits Immune Escape in Colorectal Cancer****

**In a triple co-culture system consisting of HCT116 cells, macrophages, and T cells, Western blot analysis revealed that, compared with monotherapies, combination therapy significantly downregulated PD-L1, EMT markers (Snail, Vimentin), glutamine metabolism enzymes (GLS1, GLUD1), and the chemokine CXCL12, while upregulating SMAD4 (Fig. 1A and 1B). Immunofluorescence staining showed that combination therapy significantly increased CD86 expression and decreased CD206 expression in macrophages, indicating polarization toward the anti-tumor M1 phenotype (Fig. 1C and 1D). Flow cytometry results demonstrated that combination therapy increased the proportion of CD8⁺ T cells and reduced PD-1 expression on CD8⁺ T cells (Fig. 1E**-**1G). ELISA results showed that combination therapy upregulated the secretion of GzmB, IFN-γ, and TNF-α, while downregulating IL-10 secretion (Fig. 1H**-**1K).**

Supplementary Figure 1. Targeting CXCR4 Combined with Anti-PD-1 Synergistically Reshapes the Immune Microenvironment and Inhibits Immune Escape in Colorectal Cancer. (A and B) Western blot was used to detect the expression of PD-L1, SMAD4, vimentin, Snail, GLUD1, and GLS1 under different treatment conditions. (C and D) Immunofluorescence was used to detect the expression of CD206 and CD86 in M2-type macrophages co-cultured with HCT116 cells under different treatments. (E-G) Flow cytometry analysis of the proportion of CD8⁺ T cells and PD-1 expression level after co-culturing T cells with supernatants from different macrophage groups. (H-K) ELISA was used to detect the contents of TNF-α, GzmB, IFN-γ and IL-10 in the supernatant of T cells co-cultured with the supernatants from different macrophage culture groups. ^*^*P* < 0.05, ^**^*P* < 0.01, ^***^*P* < 0.001, ^****^*P* < 0.0001.

1. **Targeting CXCR4 Sensitizes PD-1 Immunotherapy for Colorectal Cancer**

To evaluate the in vivo antitumor efficacy of α-PD-1 and AMD3100 as monotherapies or in combination, a subcutaneous CT26 tumor mouse model was established and treated accordingly (Fig. 2A). The results showed that, compared with α-PD-1 or AMD3100 monotherapy groups, the combination therapy group significantly reduced tumor volume and weight (Fig. 2B-2D). Further analysis of immune cell infiltration in the tumor microenvironment by immunofluorescence staining revealed that, compared with the control group, monotherapies moderately reduced the percentage of ARG-1-positive area (a marker of M2 macrophages) and increased the percentage of CD8-positive area (a marker of cytotoxic T cells); the combination therapy further significantly downregulated ARG-1 expression and upregulated CD8 expression (Fig. 2E and 2F).

Supplementary Figure 2. Targeting CXCR4 Sensitizes PD-1 Immunotherapy for Colorectal Cancer. (A) Flowchart of α-PD-1 and AMD3100 for in vivo treatment of xenograft tumors. (B) Images of the dissected tumors in each group. (C) Weight of the dissected tumors in each group. (D) Volume of the dissected tumors in each group. (E and F) Immunofluorescence was used to detect the expression in M2 (ARG-1) cells and CD8^+^ T cells (CD8) in tumors. ^*^*P* < 0.05, ^**^*P* < 0.01, ^***^*P* < 0.001, ^****^*P* < 0.0001.

1. **High CXCR4 Expression Suppresses CD8⁺ T Cell Infiltration in Tumor Tissue**

To further evaluate the clinical relevance of the above findings, paired tumor tissues and adjacent non‑tumor tissues from 18 patients with colorectal cancer were analyzed. Immunofluorescence staining revealed that CXCR4 expression was significantly elevated in tumor tissues compared with adjacent non‑tumor tissues, and a clear spatial correlation was observed between regions of high CXCR4 expression and reduced CD8⁺ T cell infiltration (Supplementary Fig. 3).

1. **High CXCR4 Expression Is Associated with Resistance to Anti-PD-1 Therapy**

(1) GEO database analysis

Bioinformatics analysis of a colorectal cancer cohort receiving anti-PD-1 therapy from the GEO database (GSE213331) revealed that CXCR4 mRNA expression levels in tumor tissues were significantly higher in non-responders compared with responders (Supplementary Fig. 4).

(2) Clinical sample validation

To validate the above findings at the protein level, immunohistochemical staining of CXCR4 and PD-L1 was performed on tumor samples from colorectal cancer patients (n=3) treated with PD-1 inhibitors at our center. The results showed that, compared with responders, non-responders exhibited a trend toward increased CXCR4 protein expression, accompanied by a corresponding increase in PD-L1 expression (Supplementary Fig. 5).
