# Supplementary figures and images for: Multi-omics reveals CXCR4 drives immune escape in colorectal cancer via metabolic reprogramming and immune microenvironment remodeling
Source: Cell Death Dis. 2026 May 4;17(1):591. doi: 10.1038/s41419-026-08795-x (PMC13287491; doi:10.1038/s41419-026-08795-x)

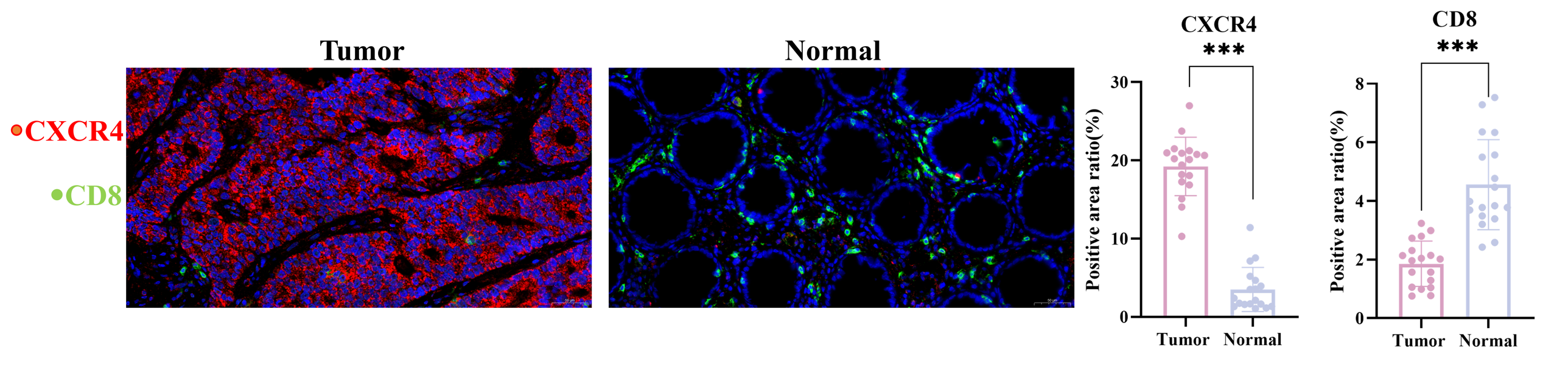

Supplement: Supplementary file 4 — Supplementary Figure 3 [file 41419_2026_8795_MOESM4_ESM.tif]

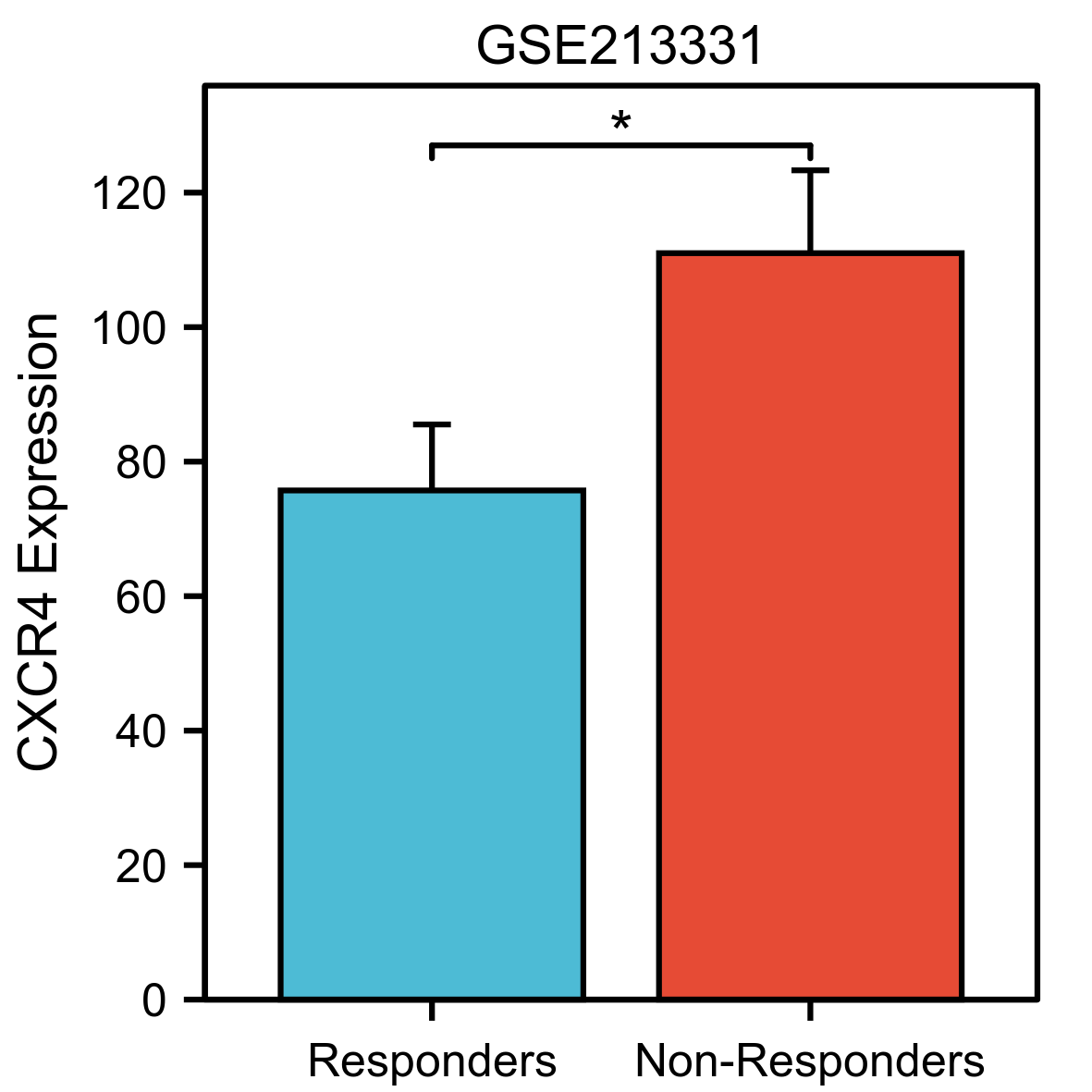

Supplement: Supplementary file 5 — Supplementary Figure 4 [file 41419_2026_8795_MOESM5_ESM.tif]
