# Supplementary material for: Multi-omics reveals CXCR4 drives immune escape in colorectal cancer via metabolic reprogramming and immune microenvironment remodeling
Source: Cell Death Dis. 2026 May 4;17(1):591. doi: 10.1038/s41419-026-08795-x (PMC13287491; doi:10.1038/s41419-026-08795-x)

Fig.1E

CXCR4

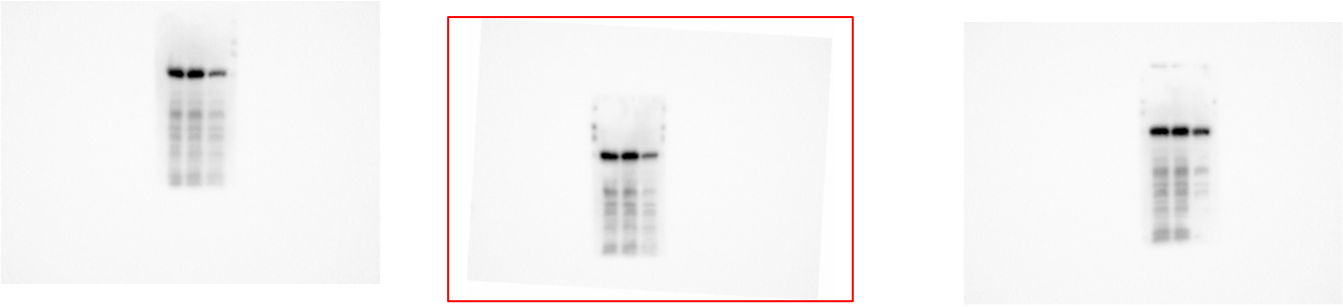

β-actin

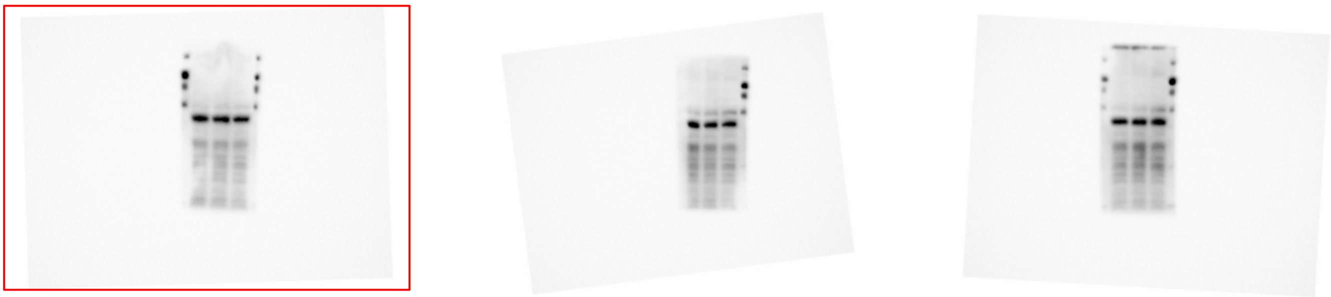

Fig.4A

CXCR4

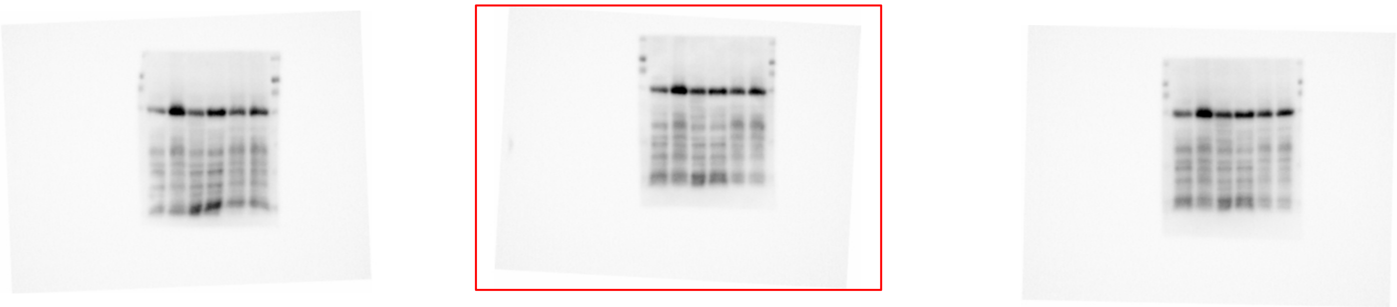

β-actin

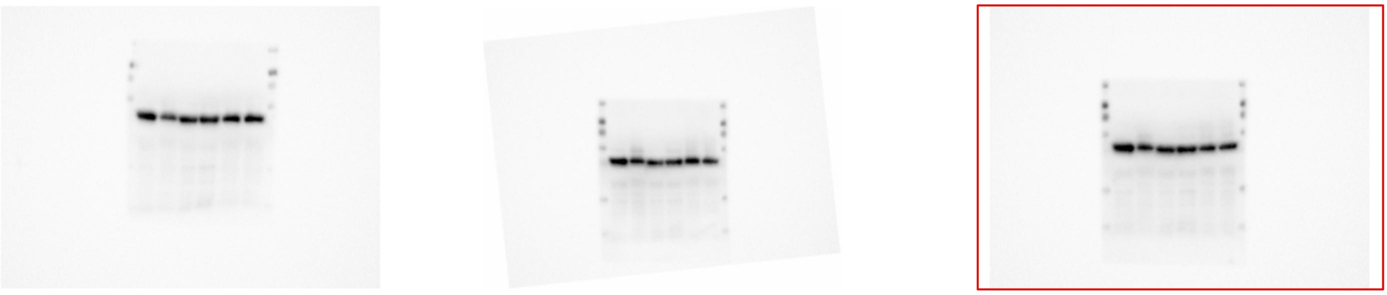

Fig.4C

CXCR4

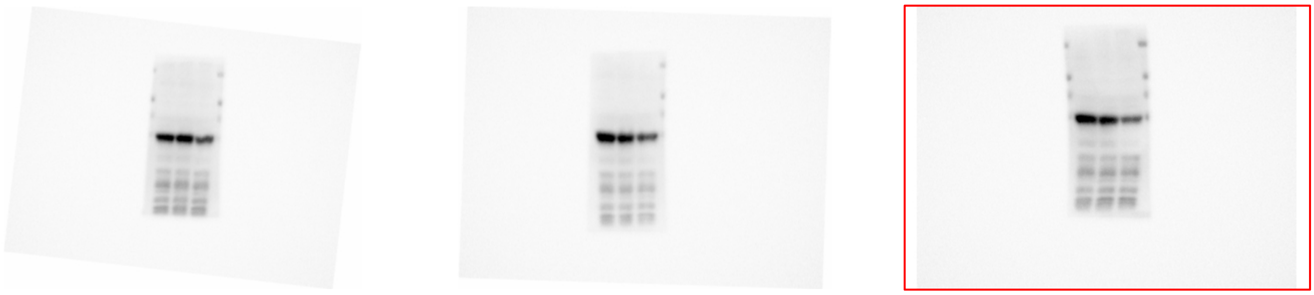

β-actin

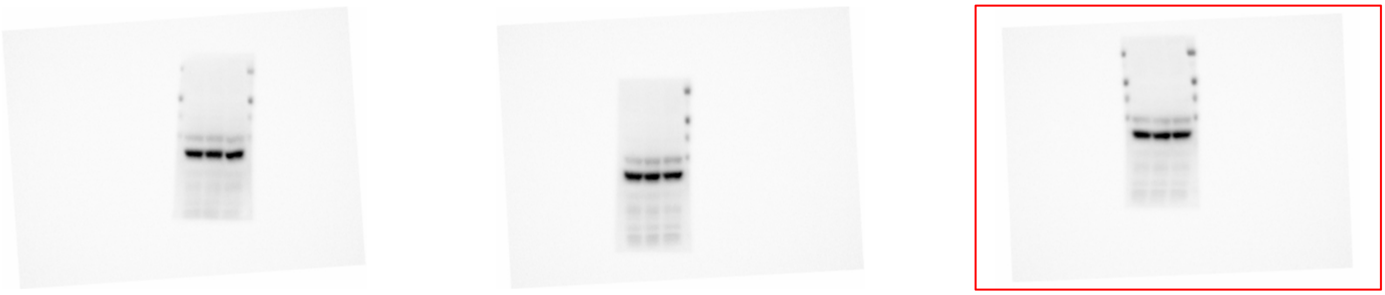

Fig.4E

PD-L1

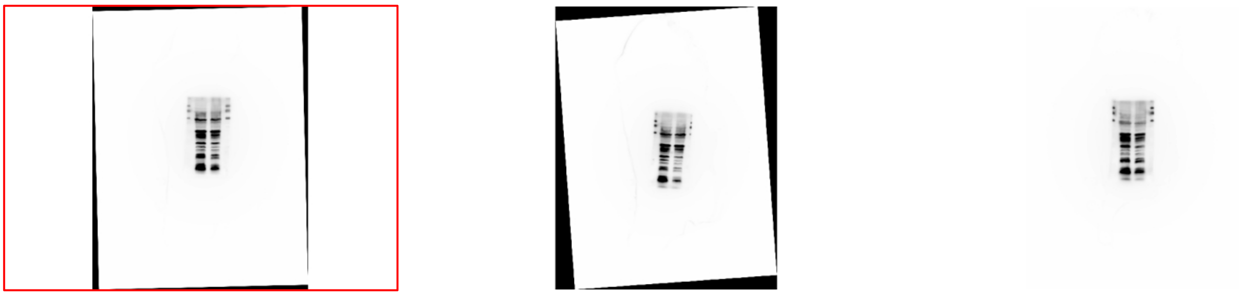

β-actin

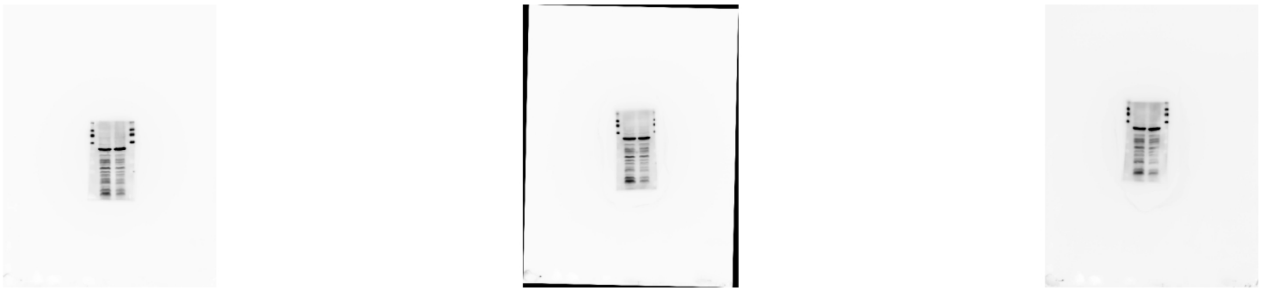

SMAD4

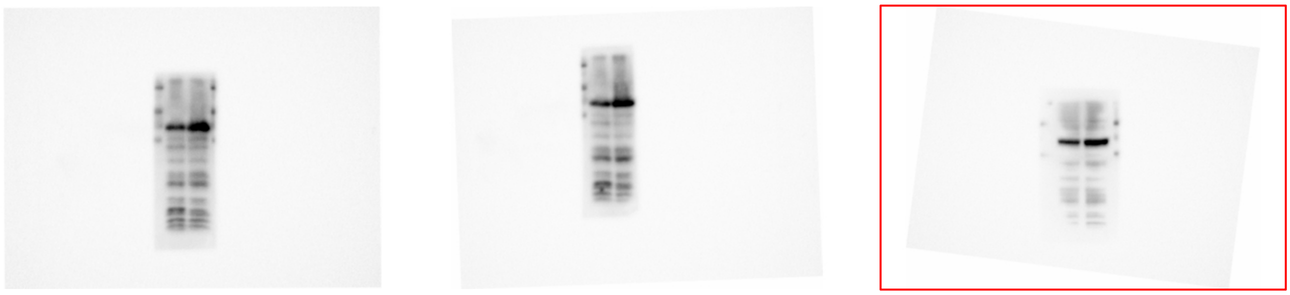

β-actin

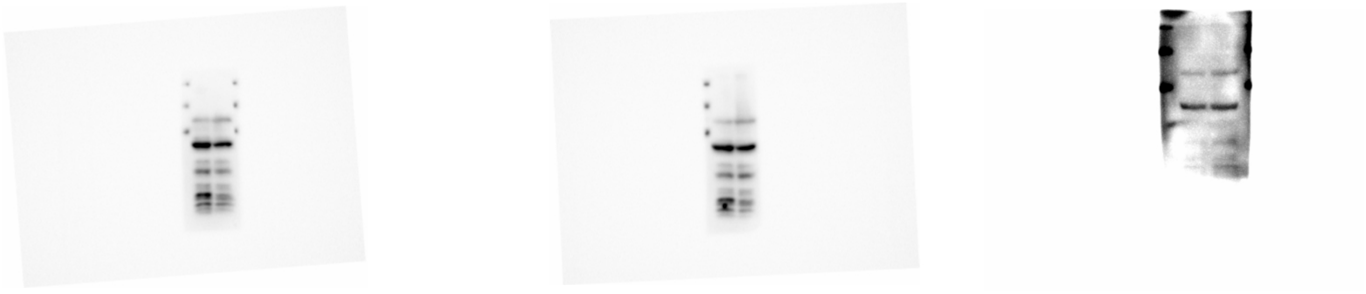

Vimentin

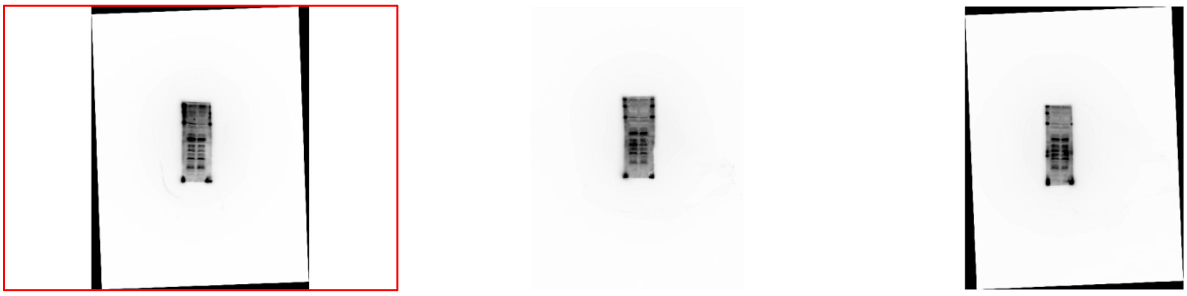

β-actin

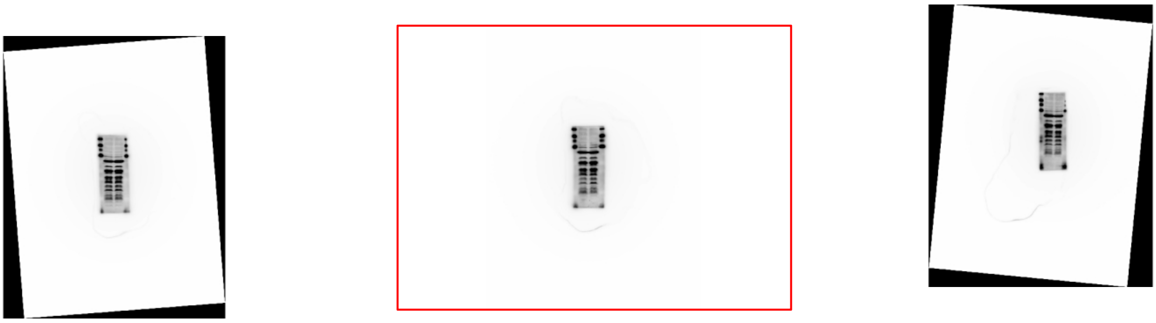

Snail

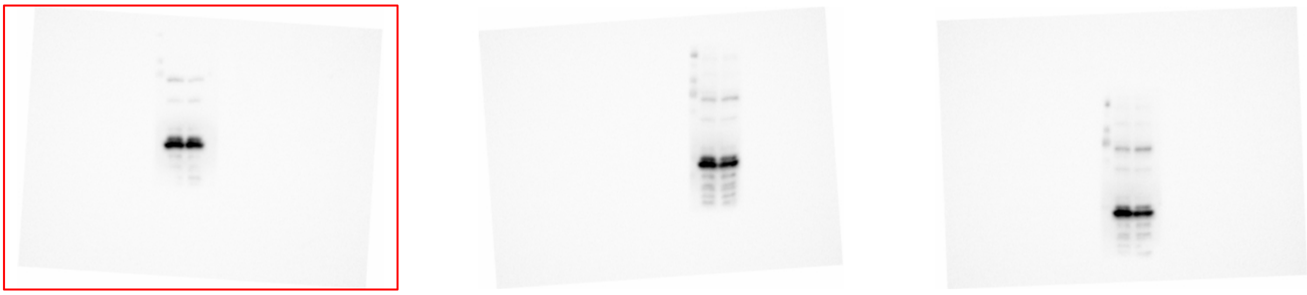

β-actin

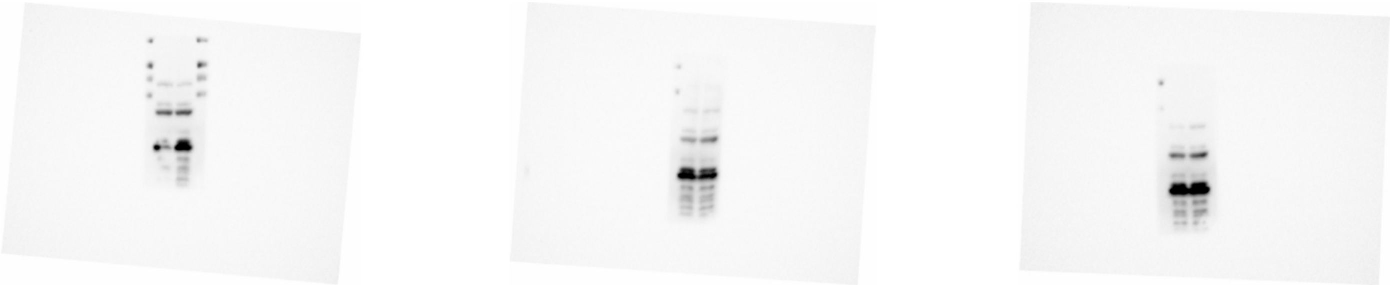

GLS1

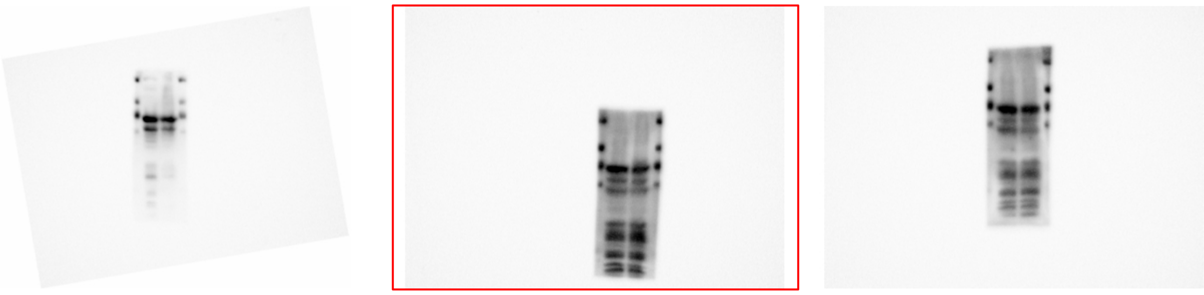

β-actin

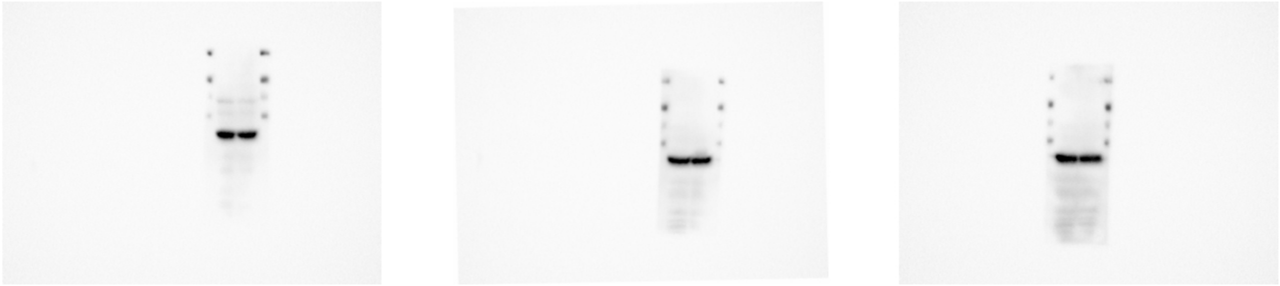

GLUD1

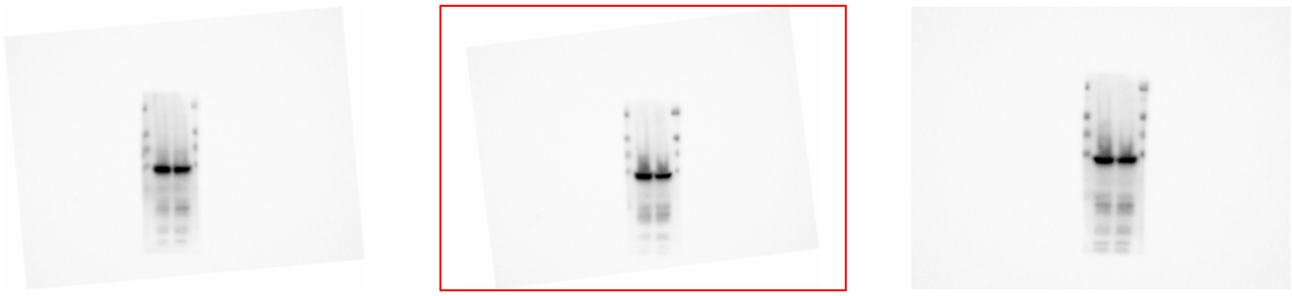

β-actin

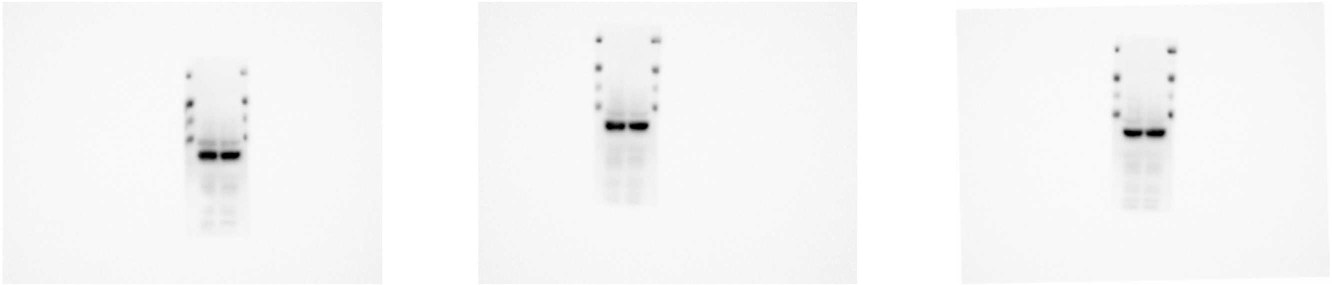

Fig.4H

PD-L1

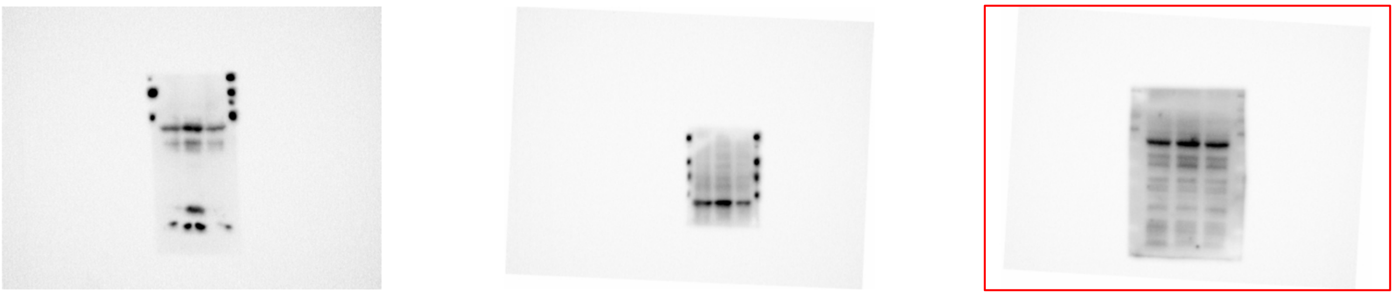

β-actin

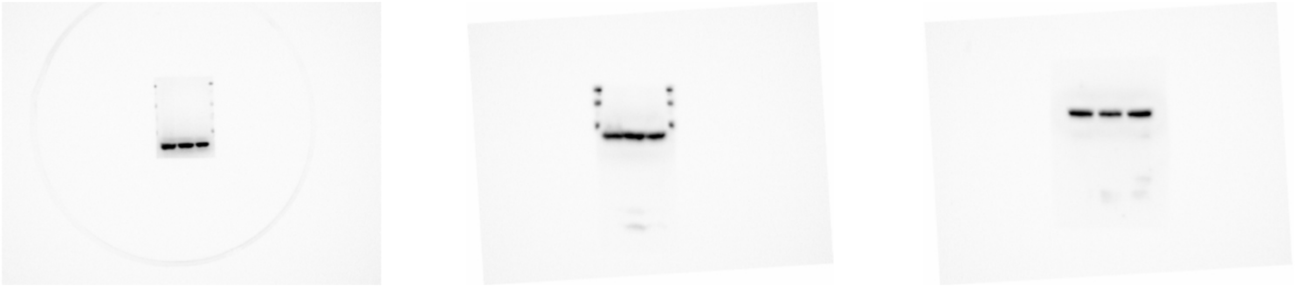

SMAD4

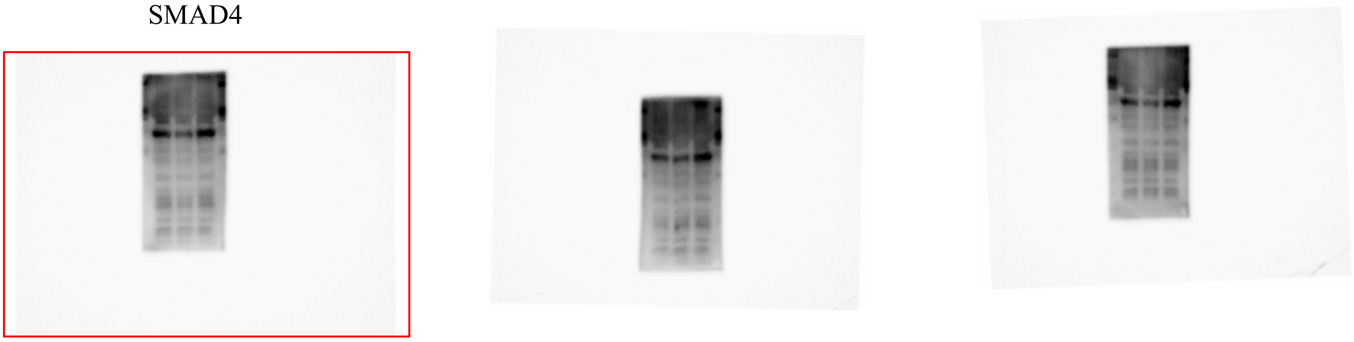

β-actin

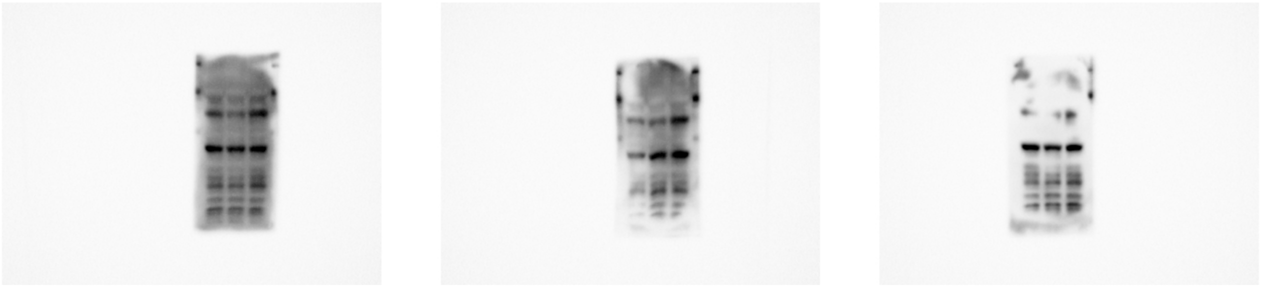

Vimentin

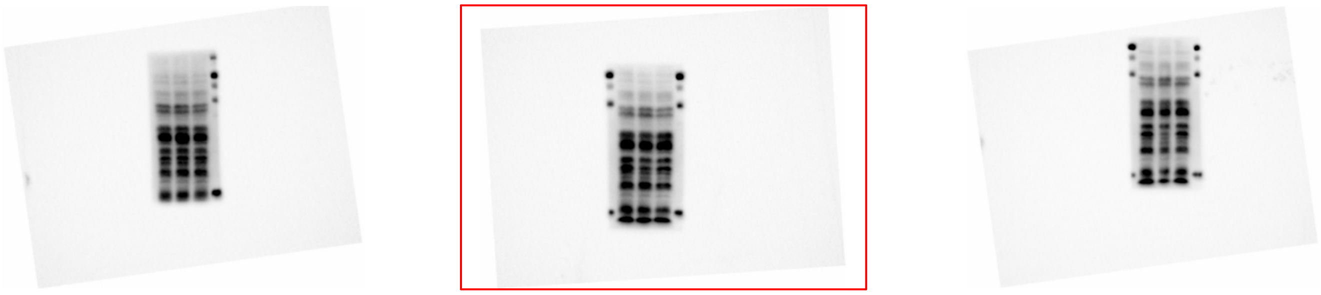

β-actin

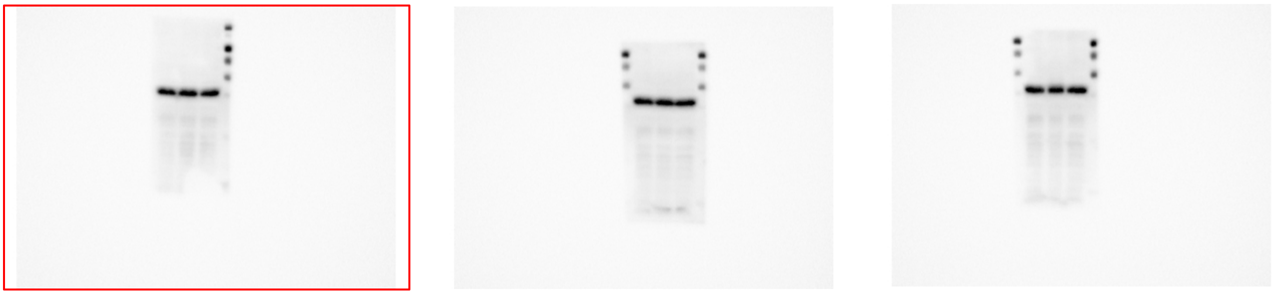

Snail

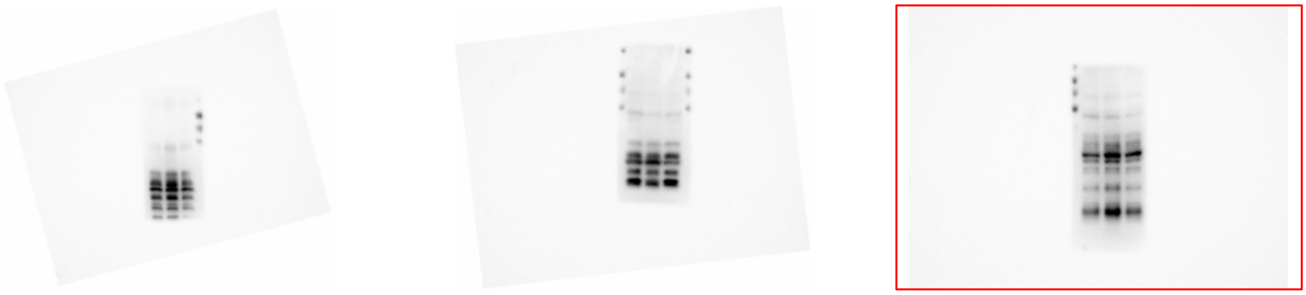

β-actin

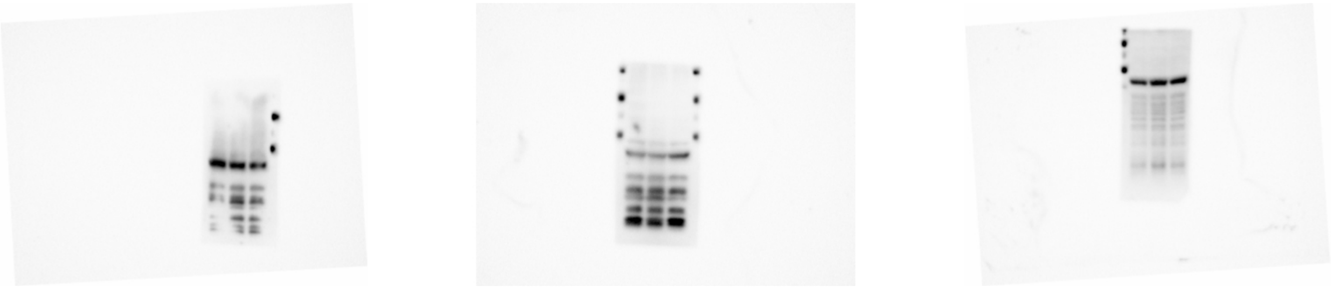

GLUD1

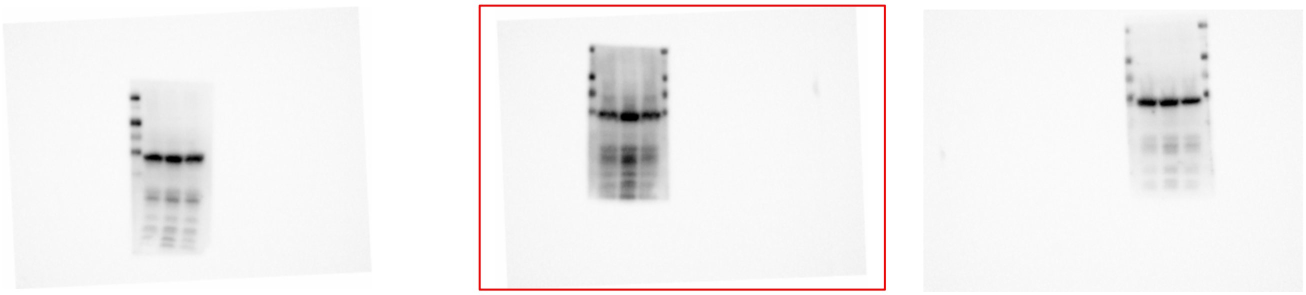

β-actin

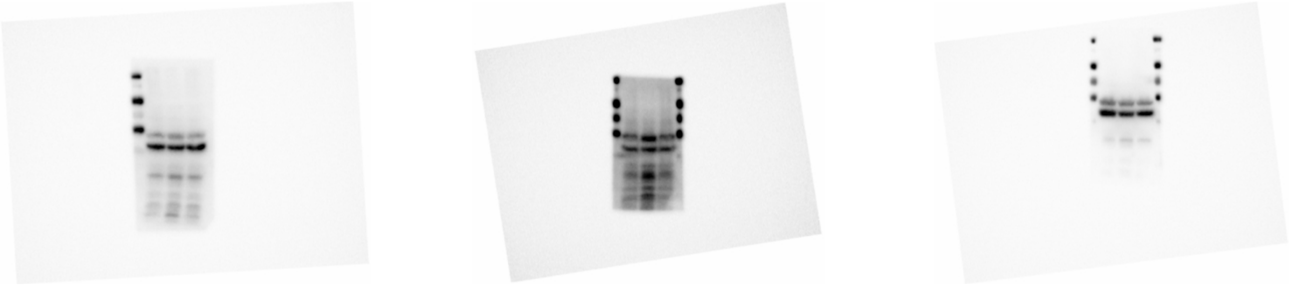

GLS1

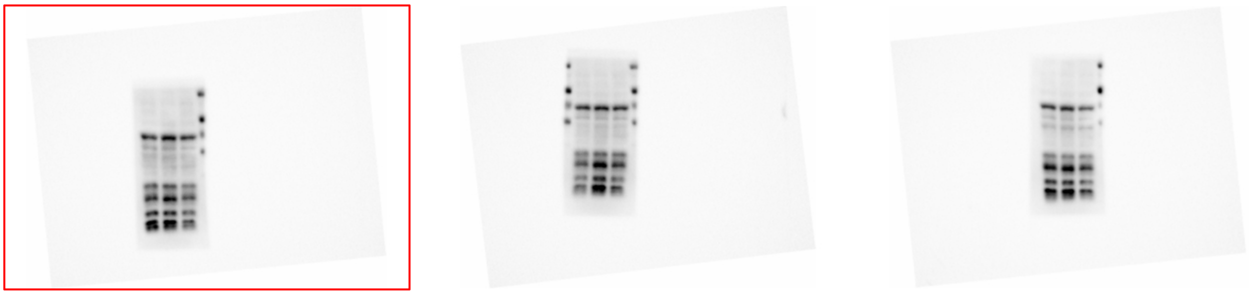

β-actin

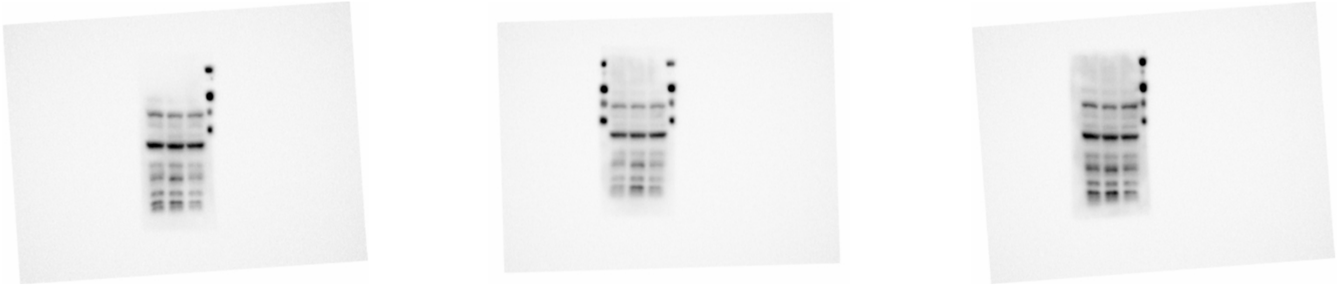

Fig.4L

P-AKT

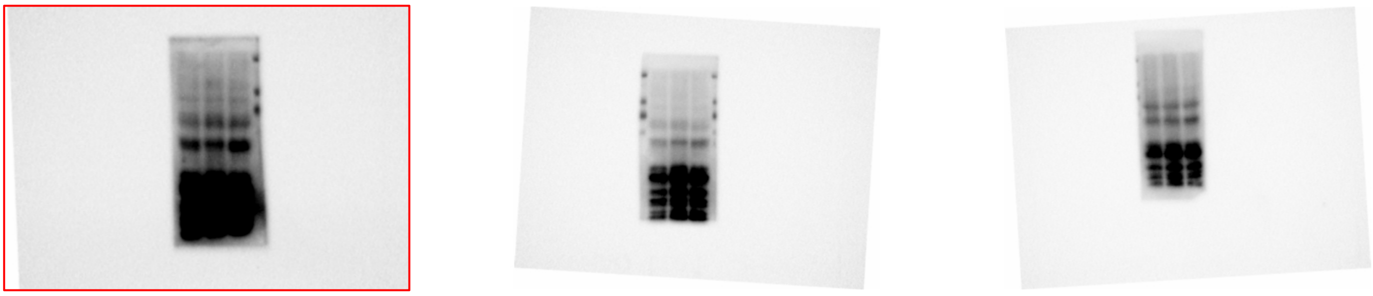

AKT

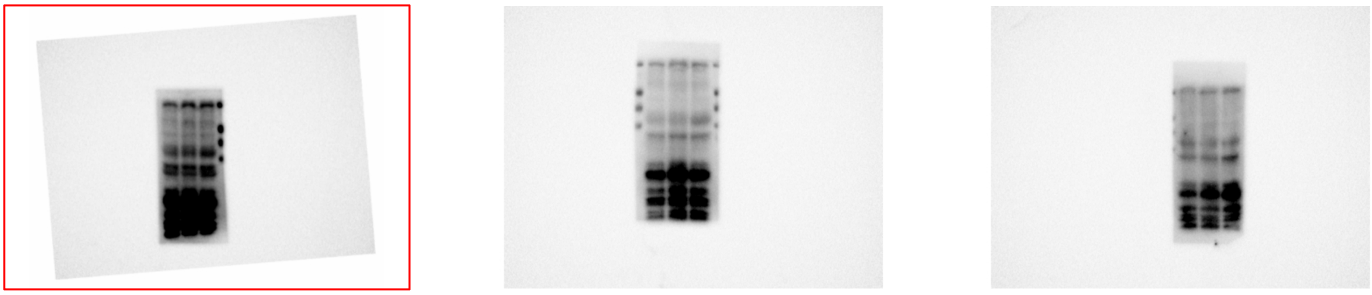

β-actin

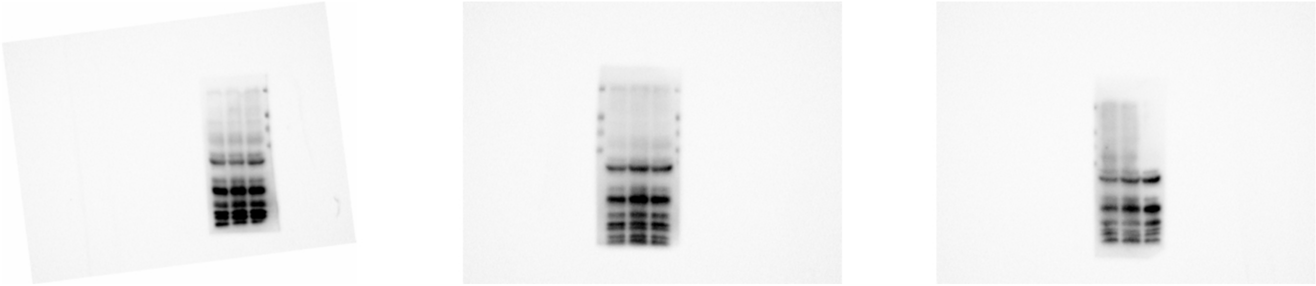

PD-L1

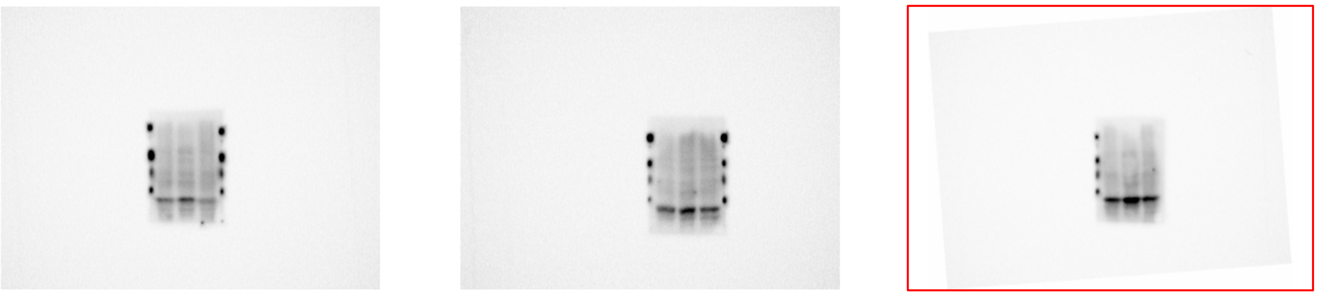

β-actin

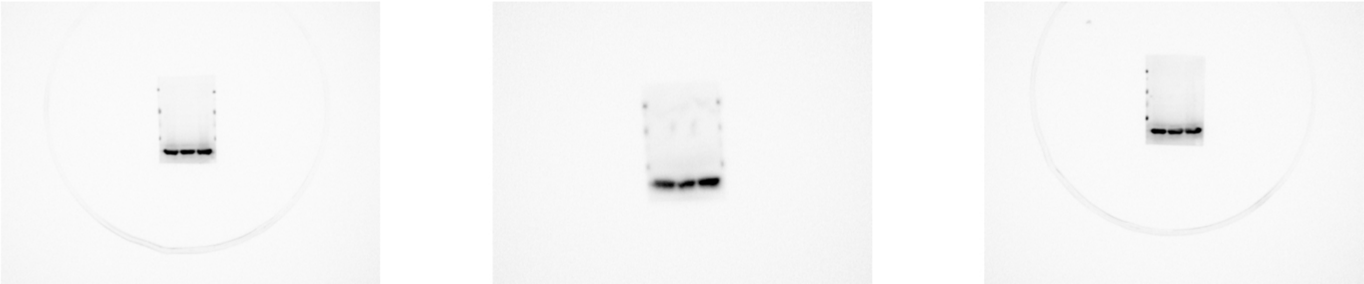

SMAD4

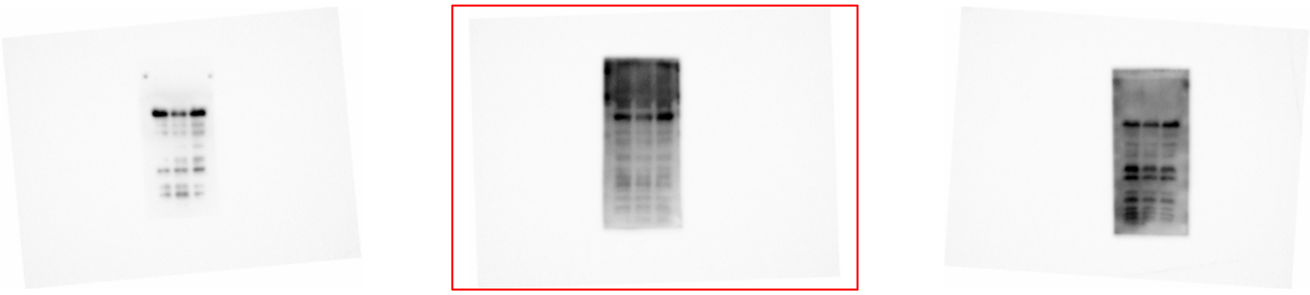

β-actin

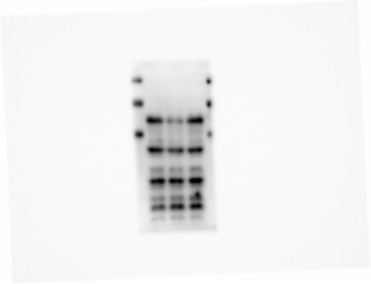
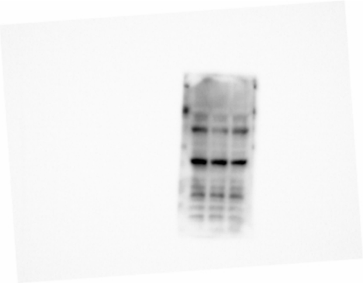
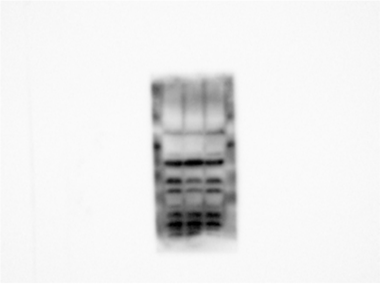

Vimentin

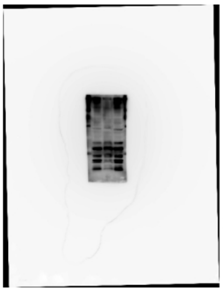
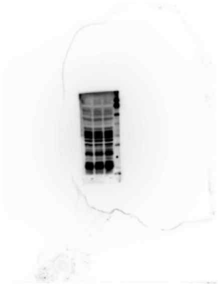
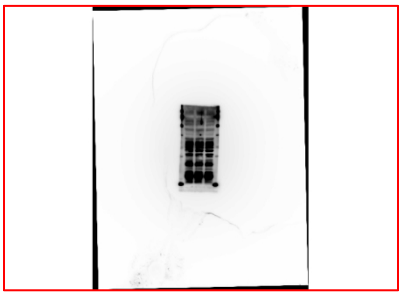

β-actin

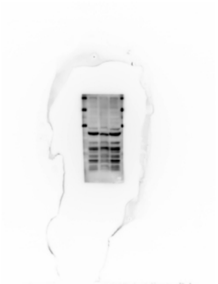
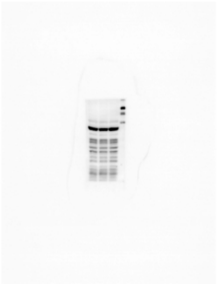
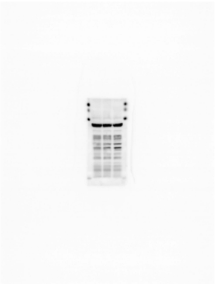

Snail

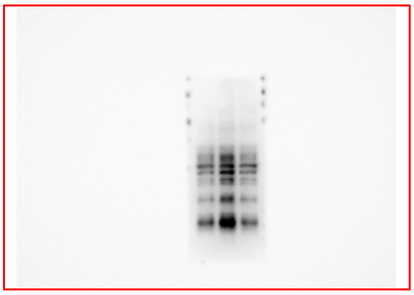
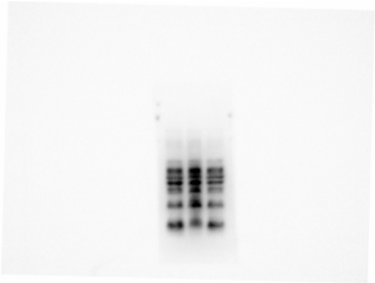
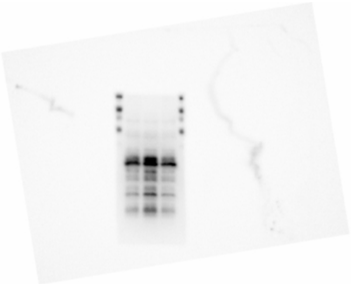

β-actin

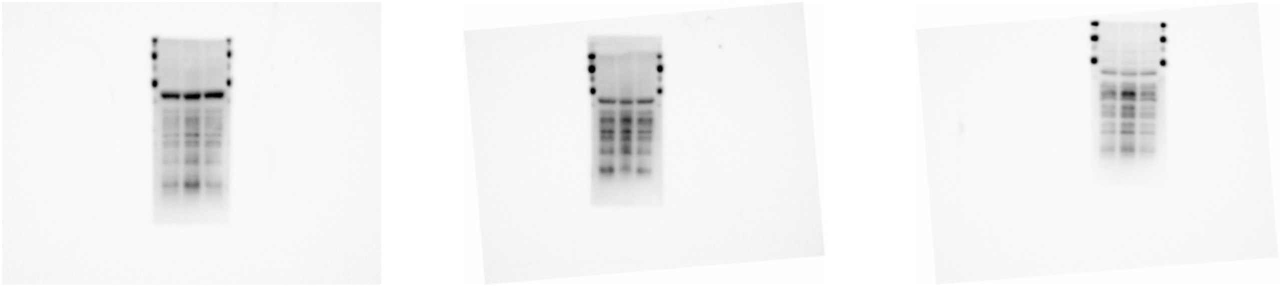

GLUD1

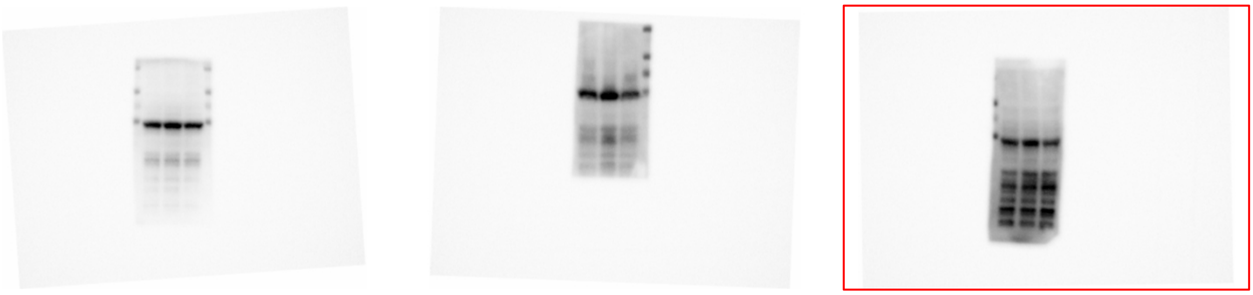

β-actin

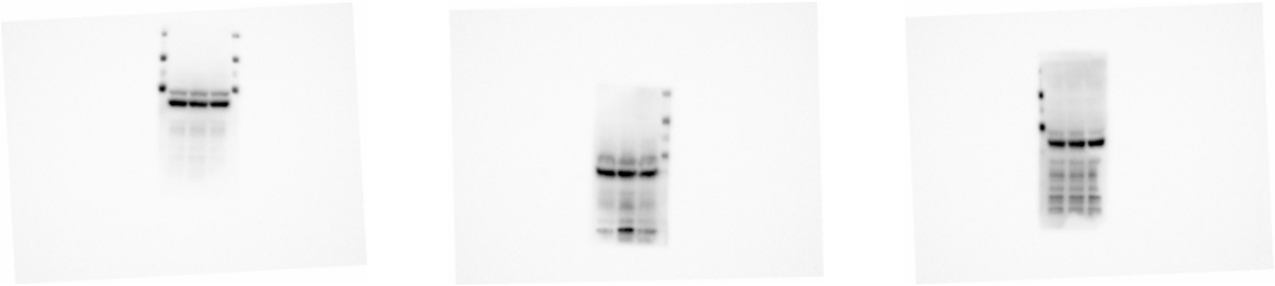

GLS1

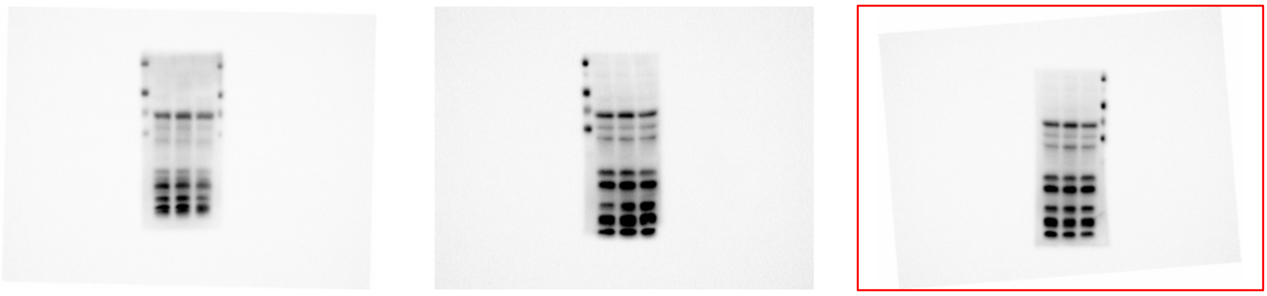

β-actin

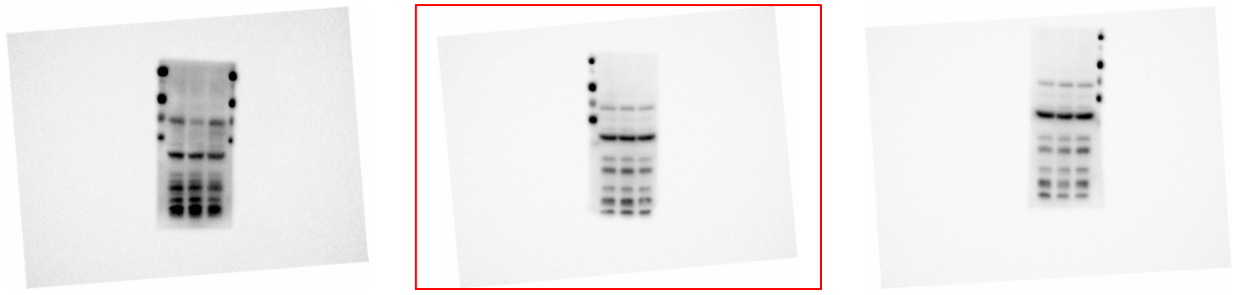

Fig.5C

SMAD4

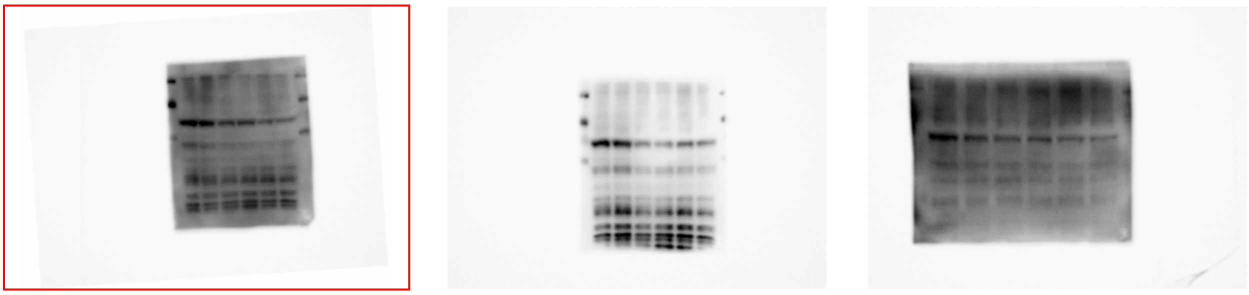

β-actin

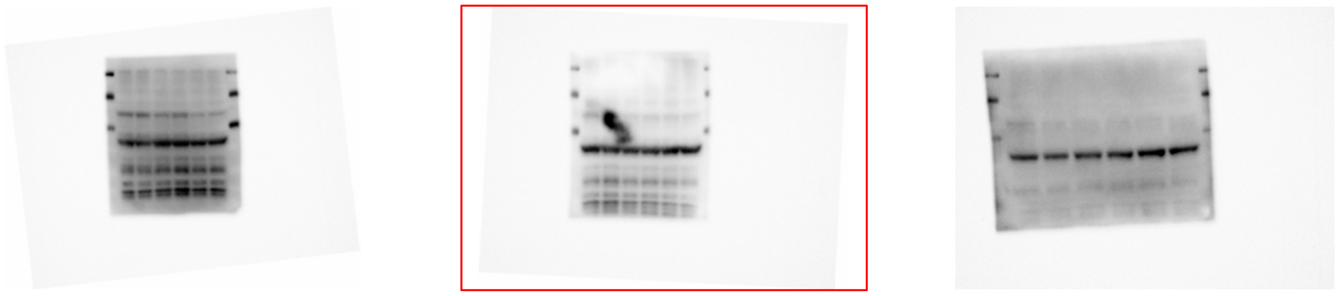

Fig.5E

PD-L1

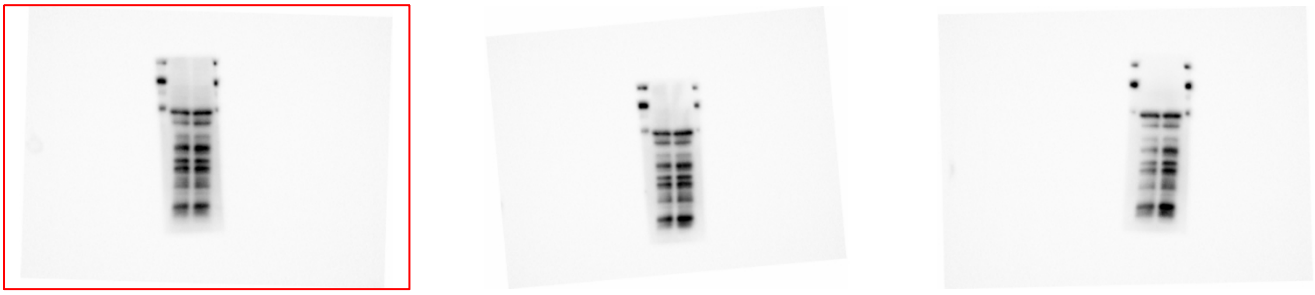

β-actin

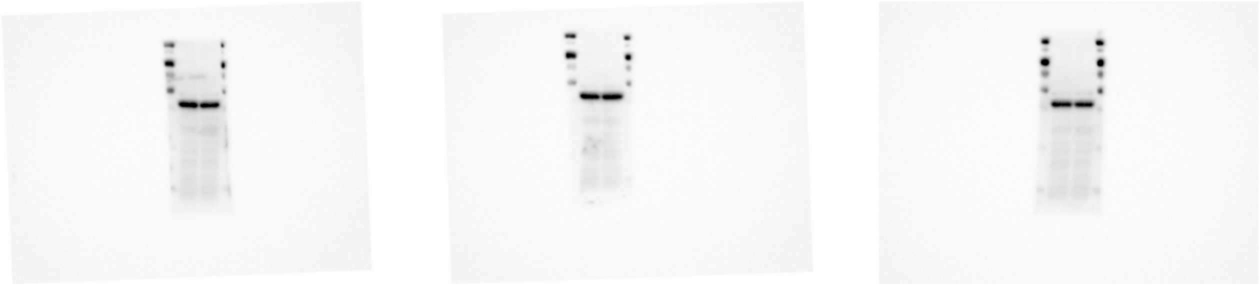

Vimentin

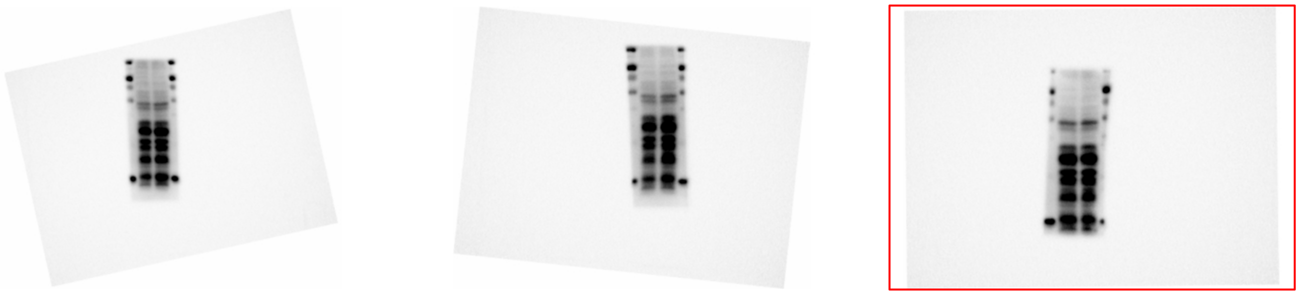

β-actin

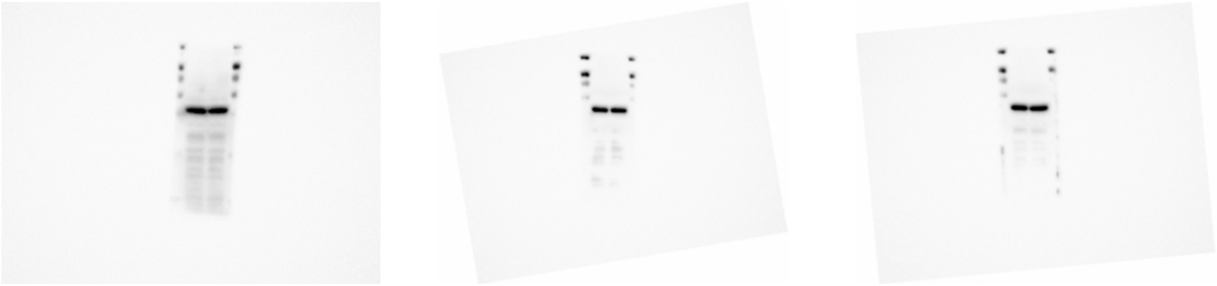

Snail

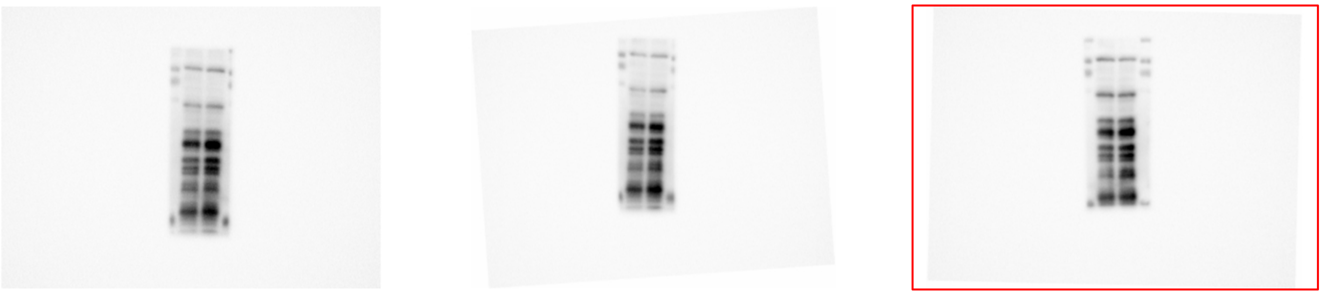

β-actin

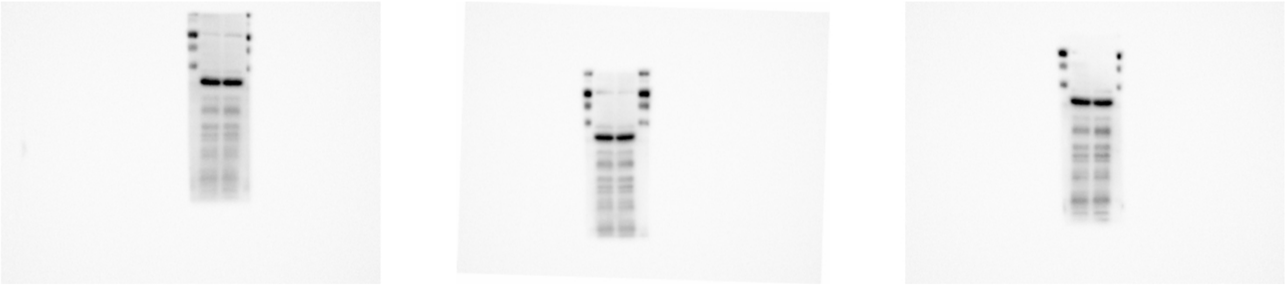

GLUD1

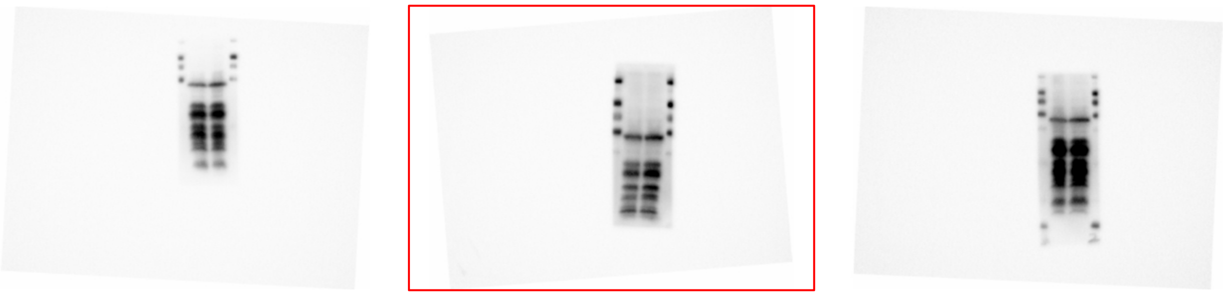

β-actin

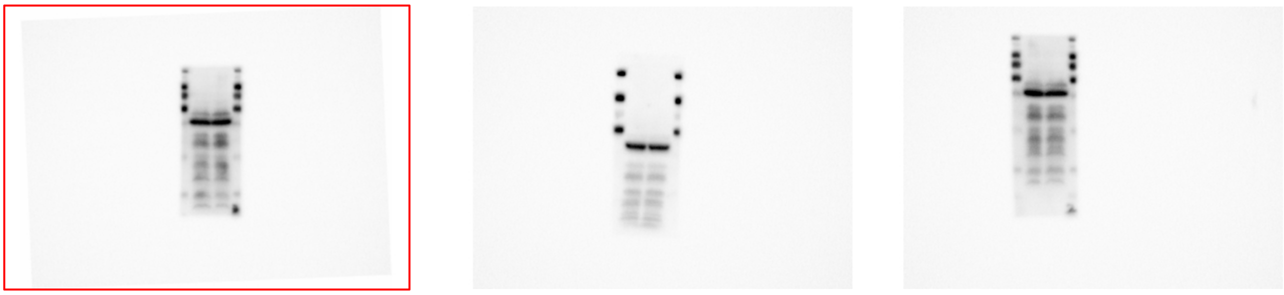

GLS1

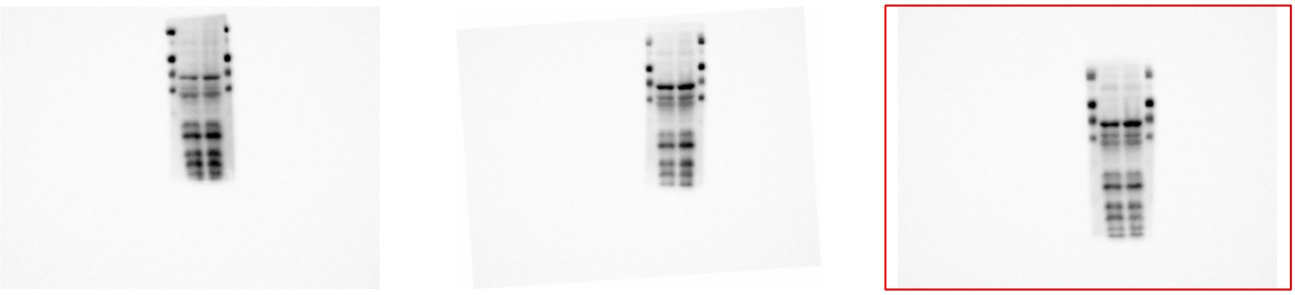

β-actin

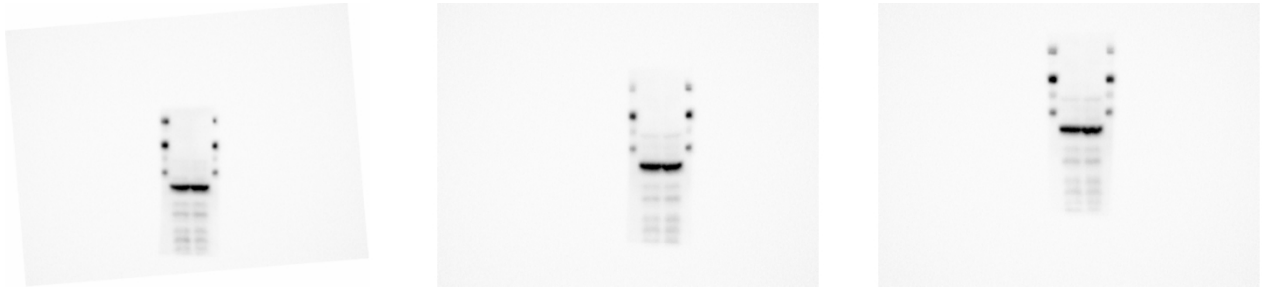

CXCL12

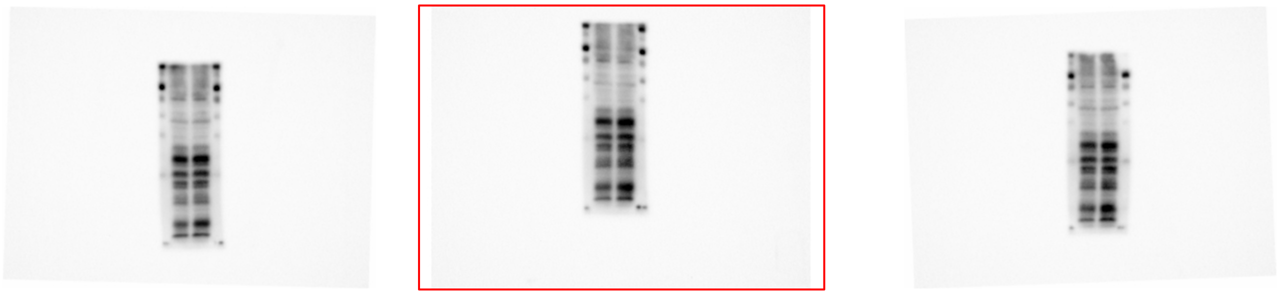

β-actin

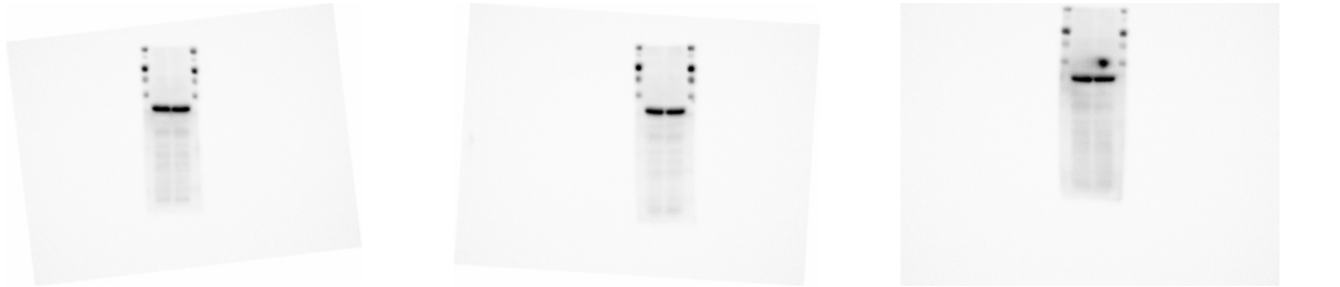

Fig.5H

PD-L1

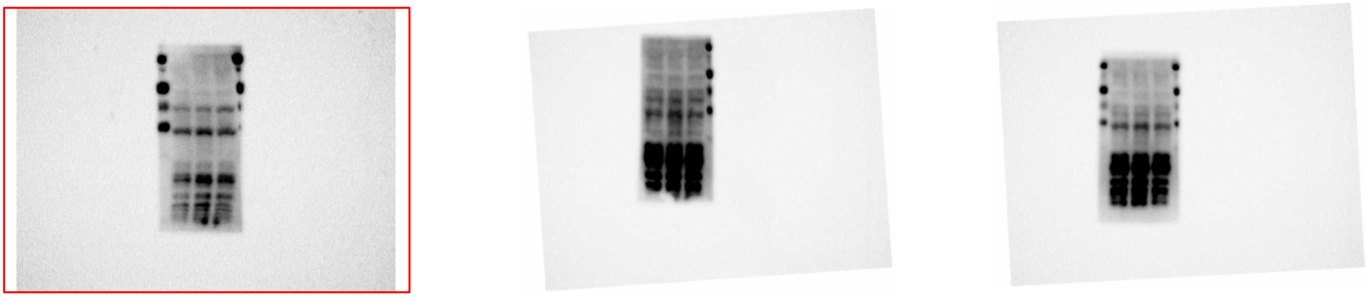

β-actin

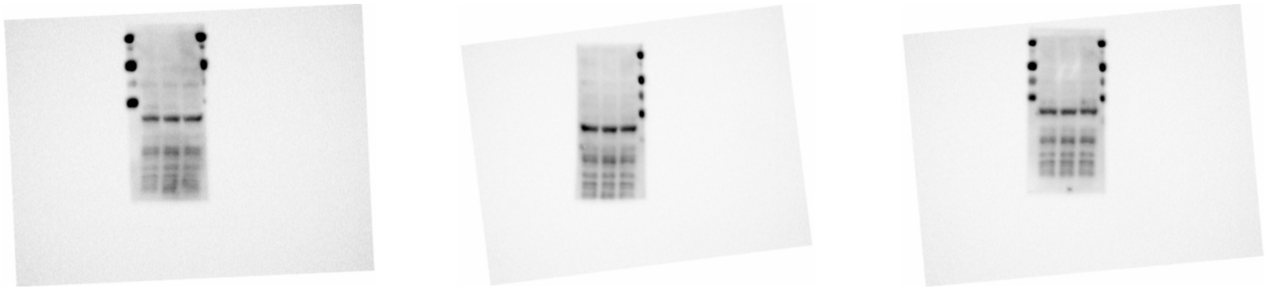

Vimentin

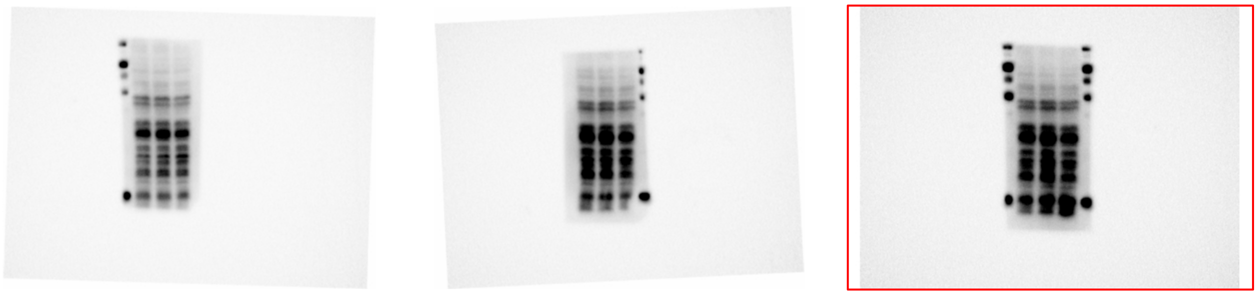

β-actin

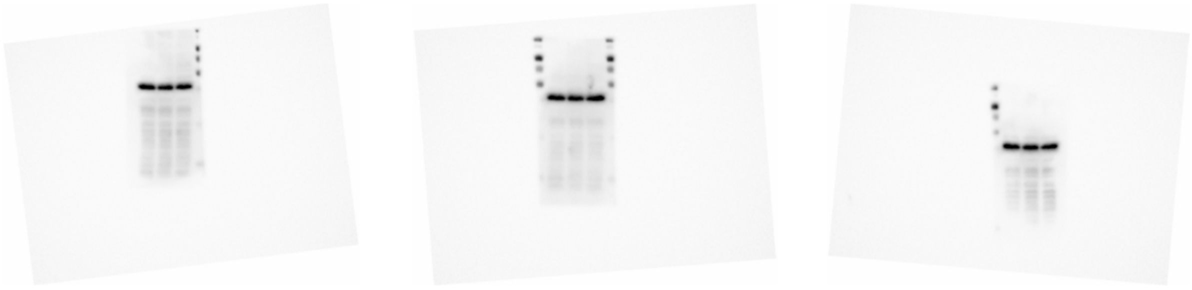

Snail

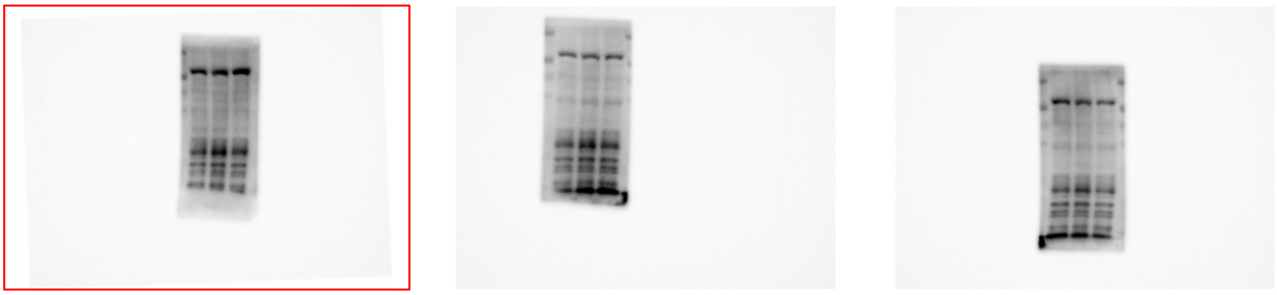

β-actin

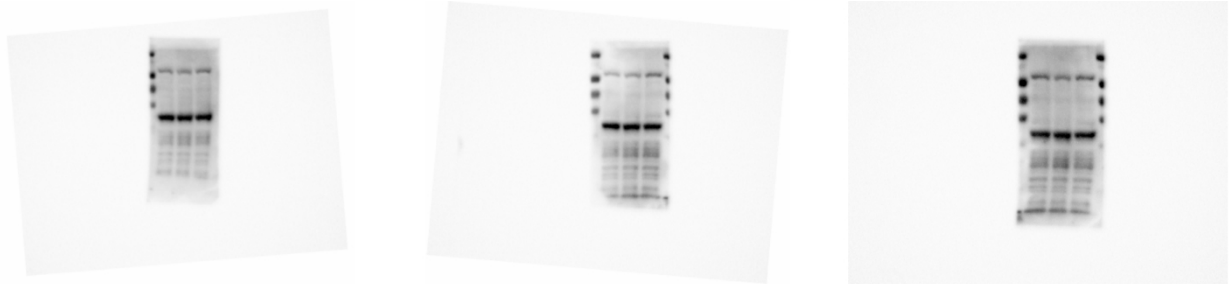

GLUD1

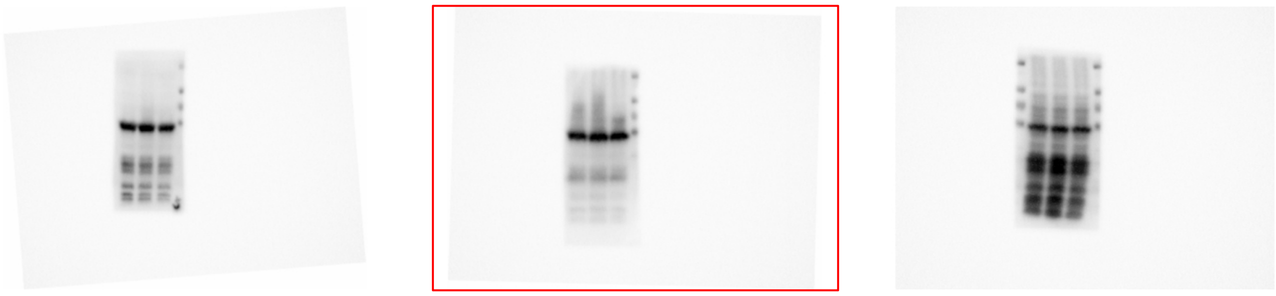

β-actin

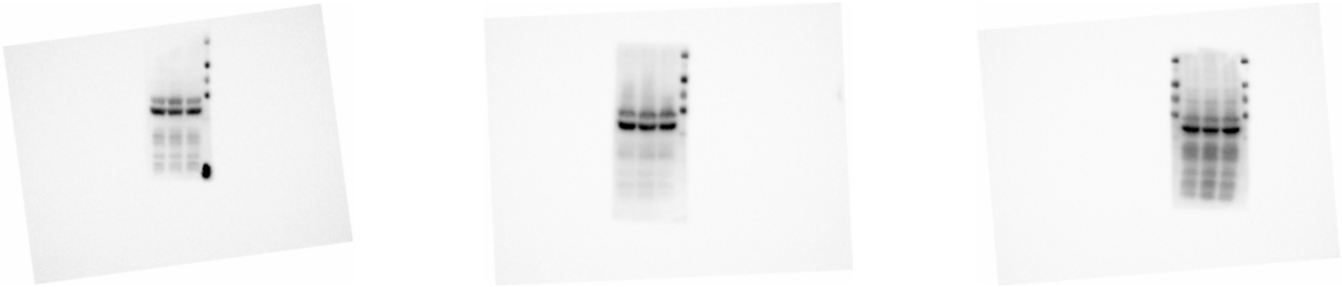

GLS1

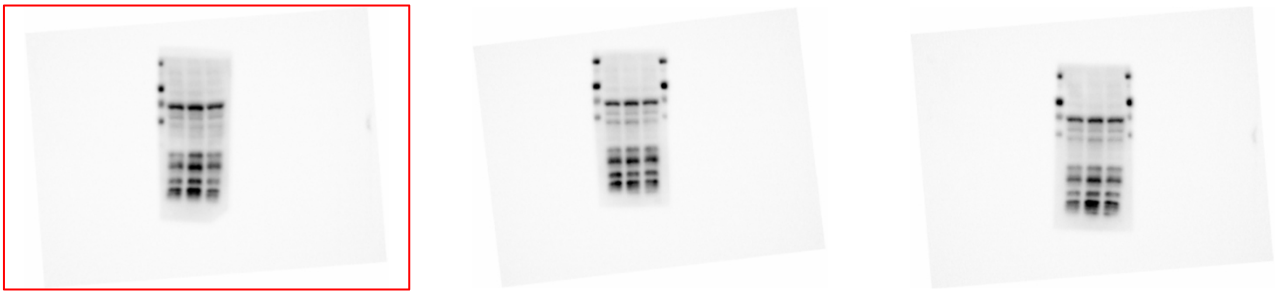

β-actin

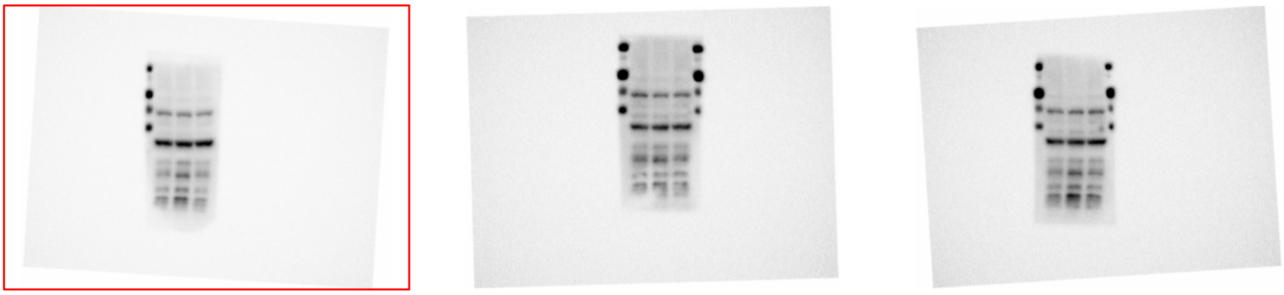

Fig.5K

PD-L1

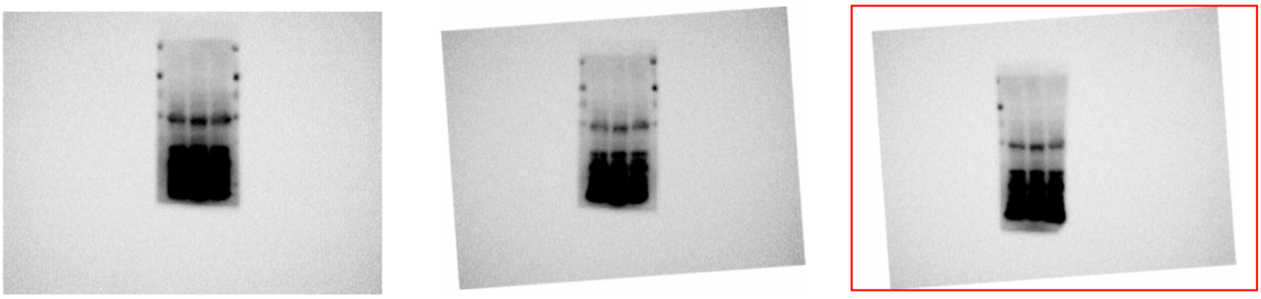

β-actin

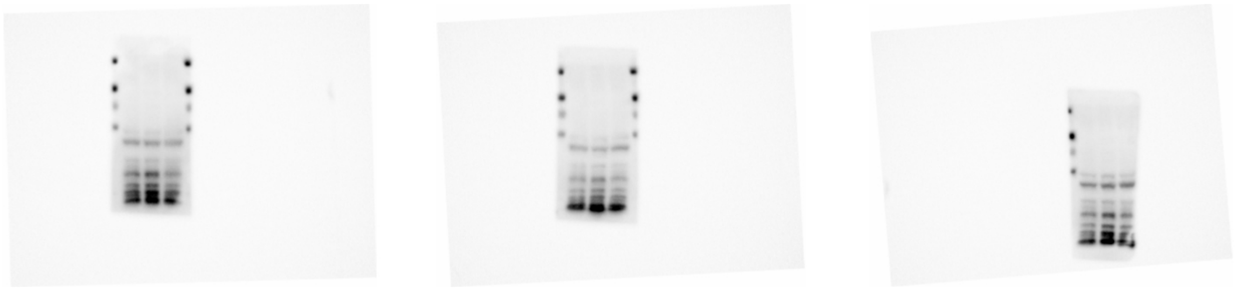

Vimentin

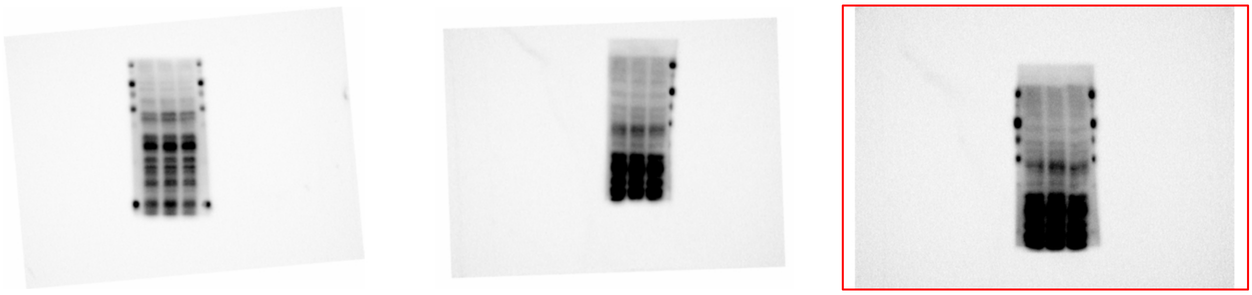

β-actin

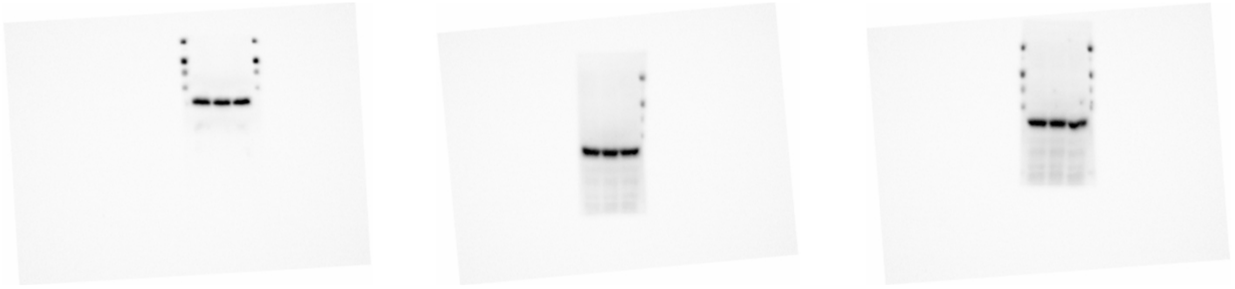

Snail

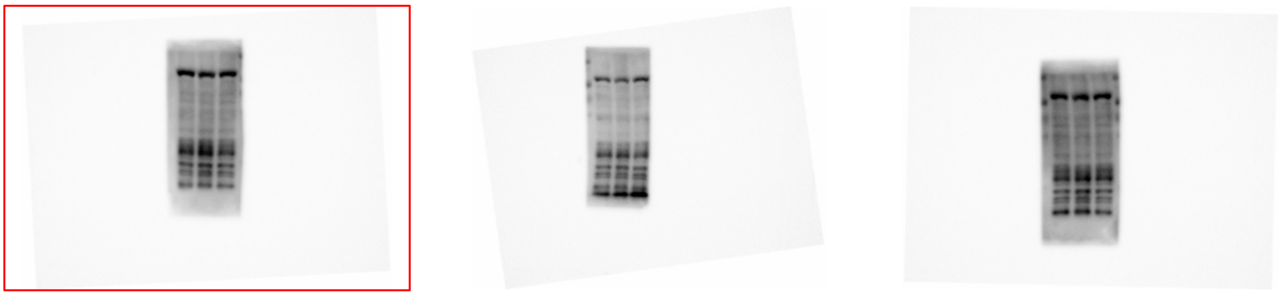

β-actin

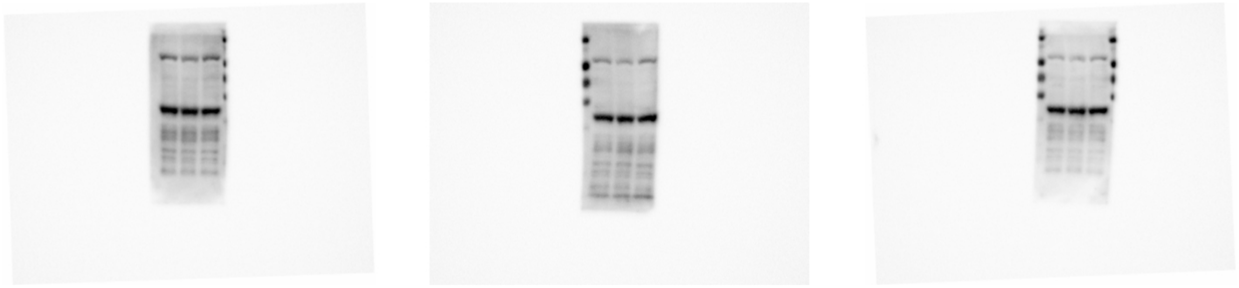

GLUD1

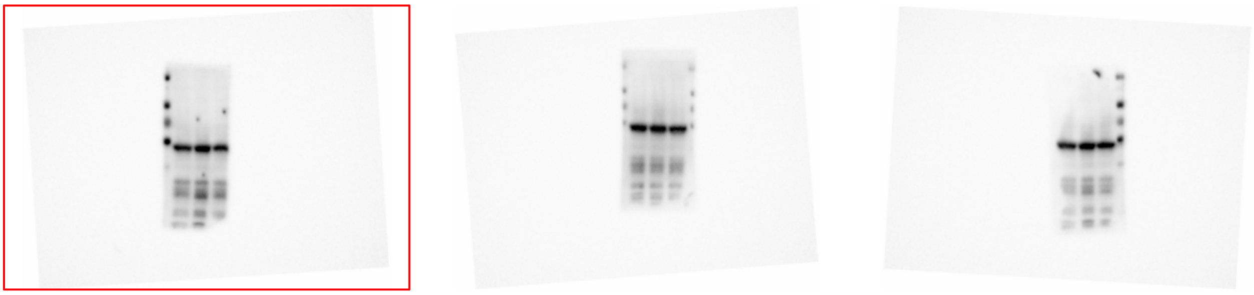

β-actin

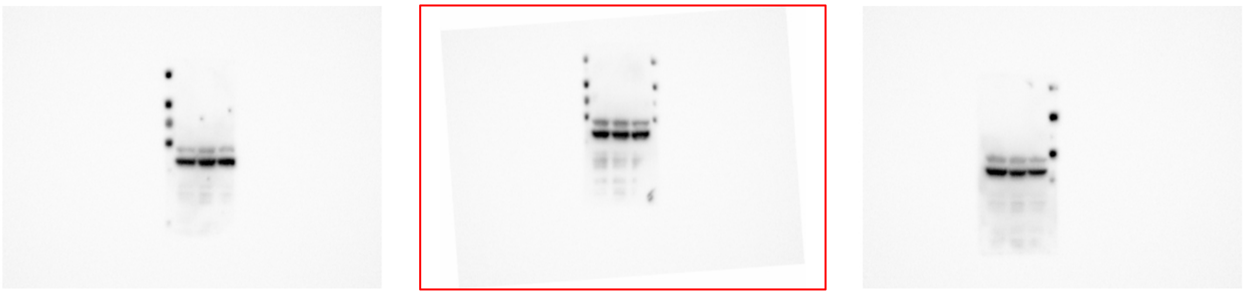

GLS1

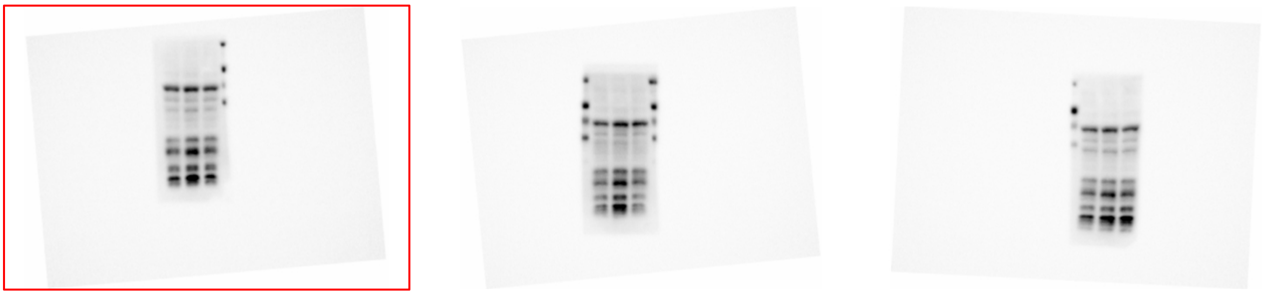

β-actin

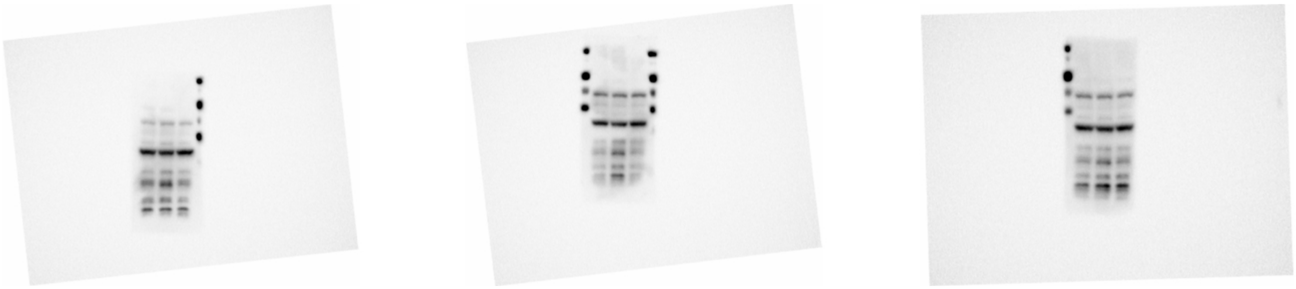

CXCL12

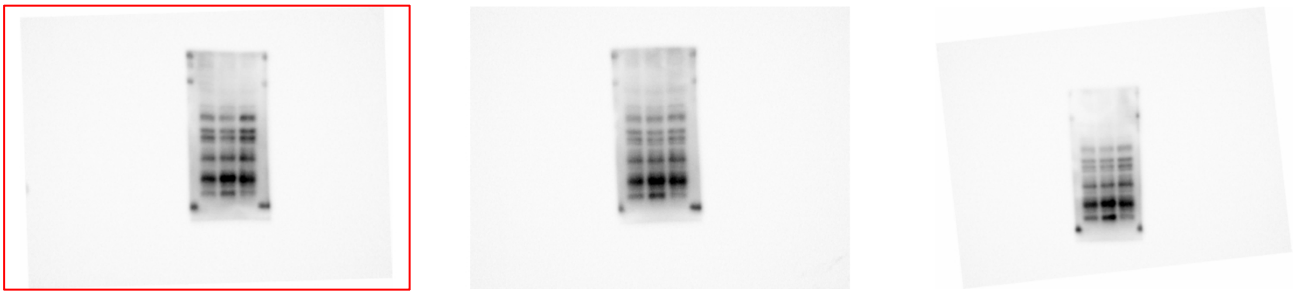

β-actin

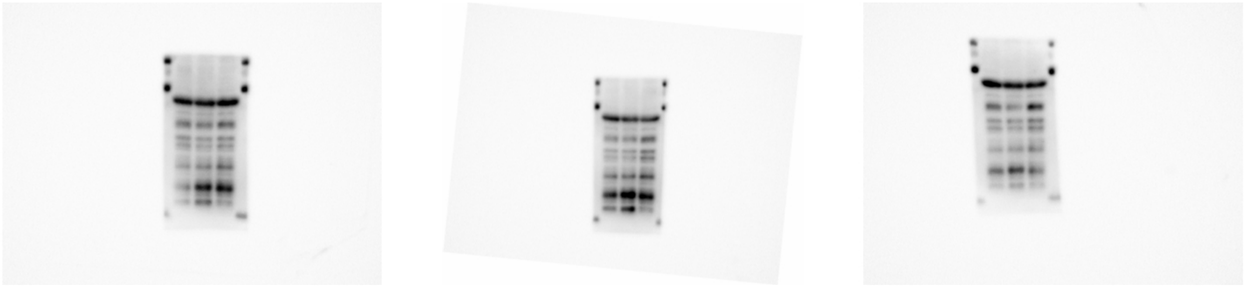

Fig.5N

PD-L1

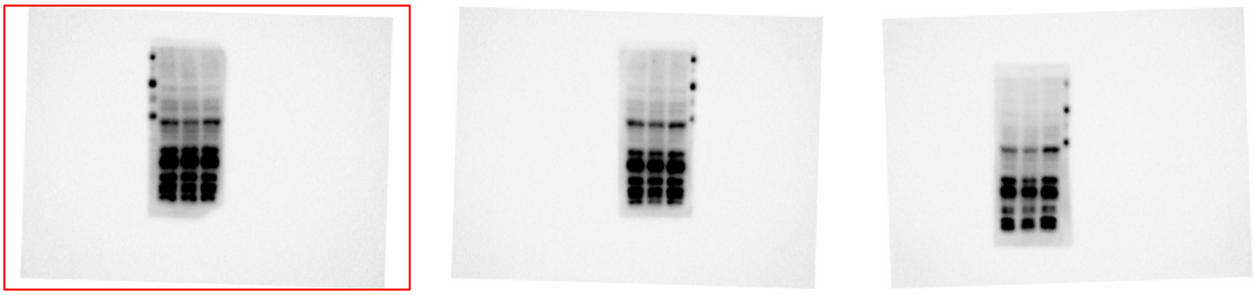

β-actin

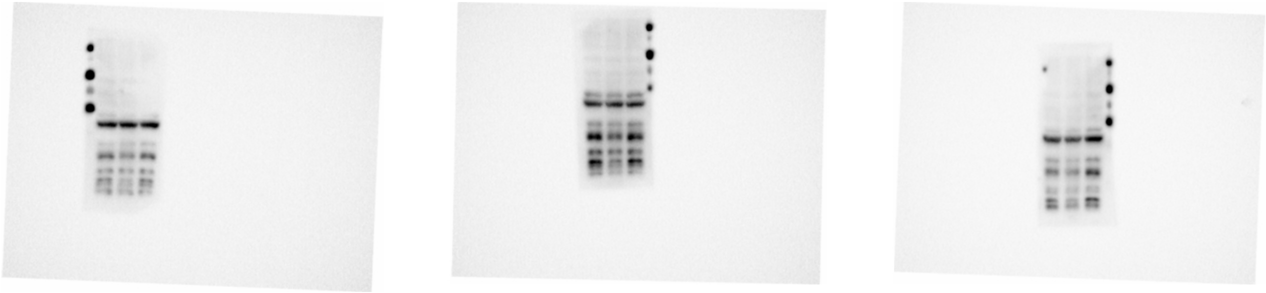

Vimentin

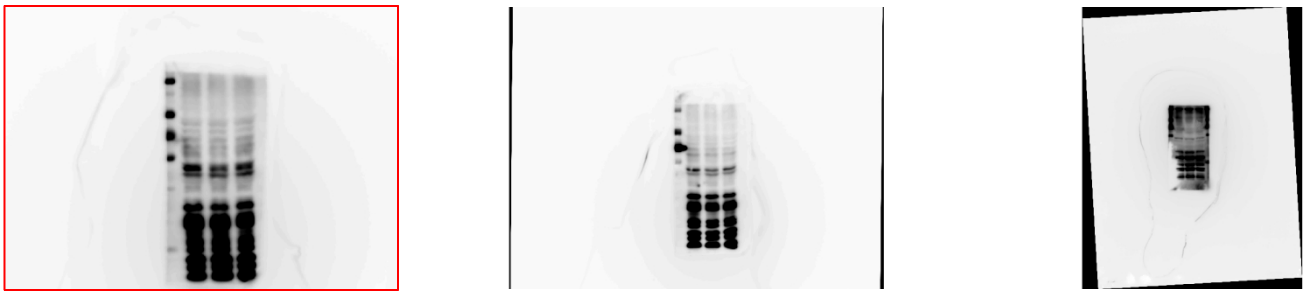

β-actin

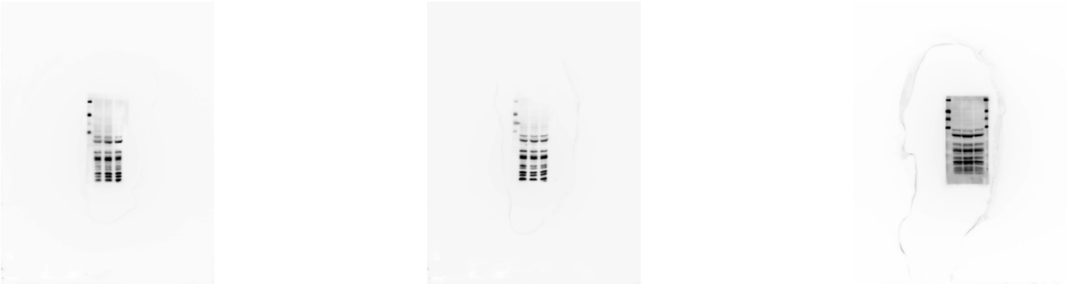

Snail

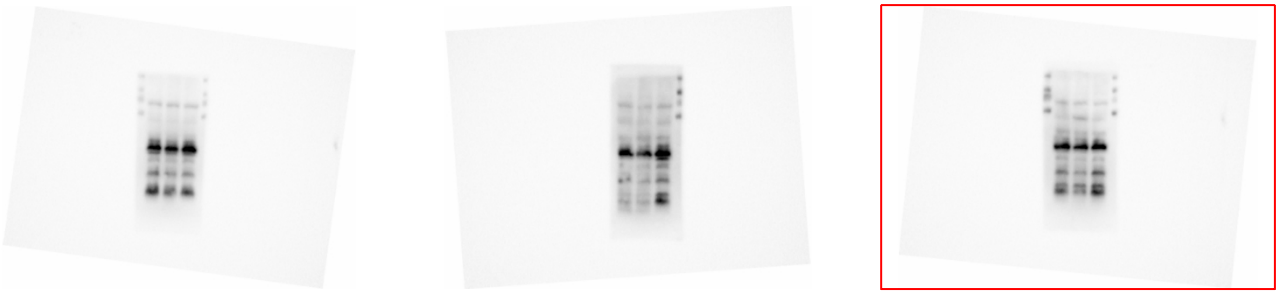

β-actin

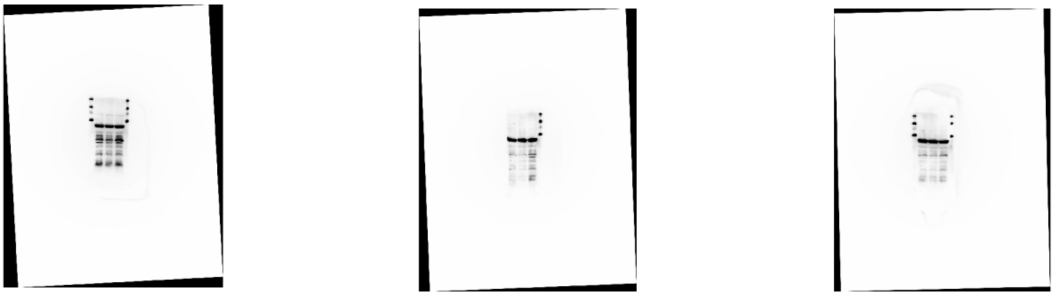

GLUD1

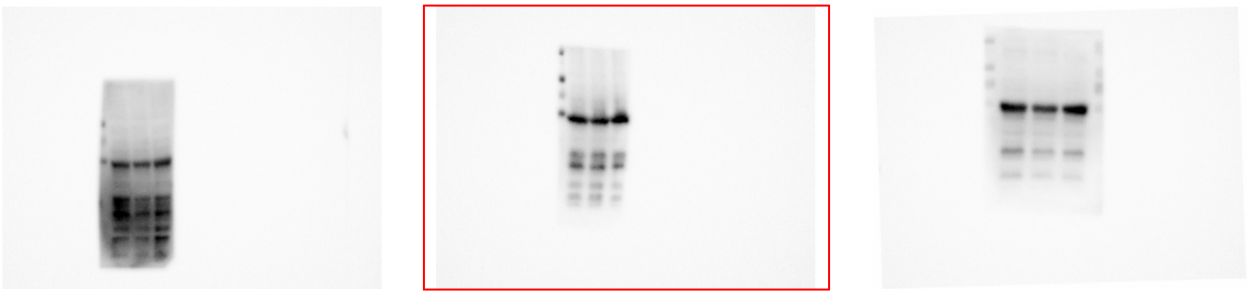

β-actin

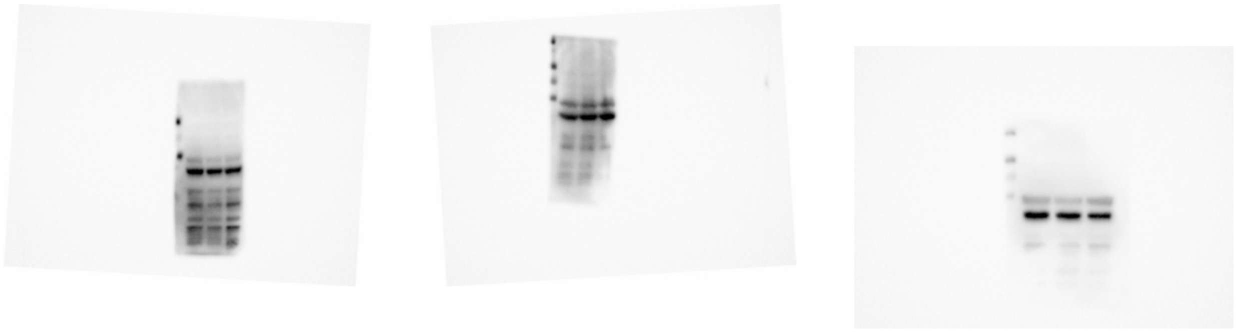

GLS1

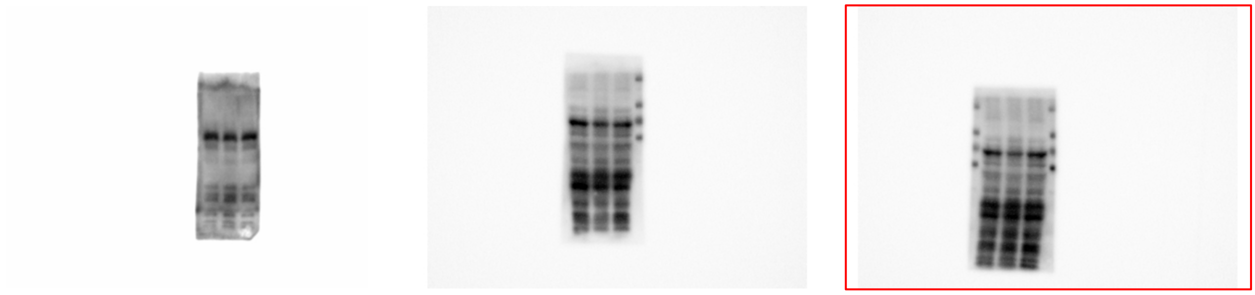

β-actin

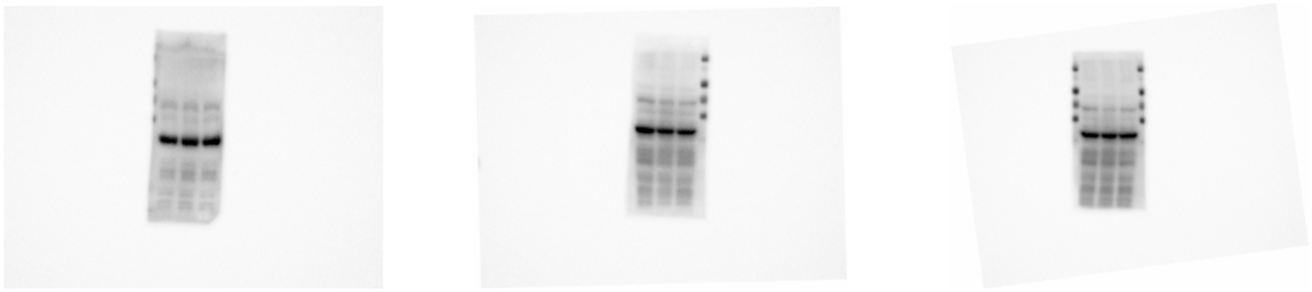

CXCL12

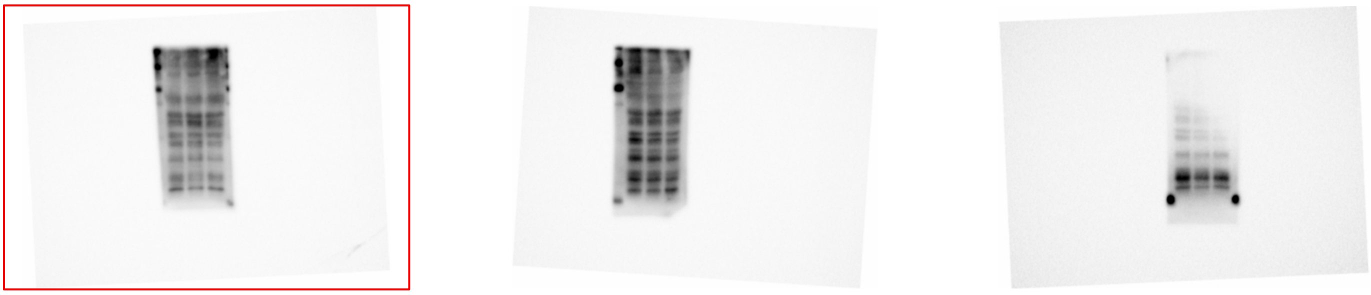

β-actin

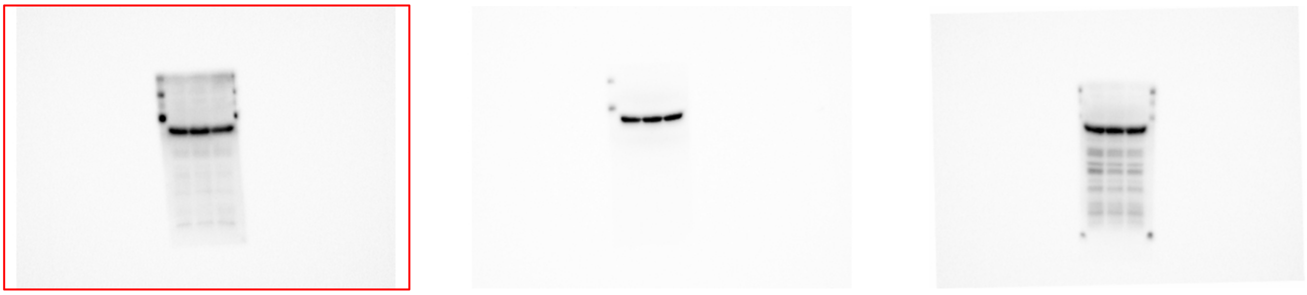

Fig.6F

PD-L1

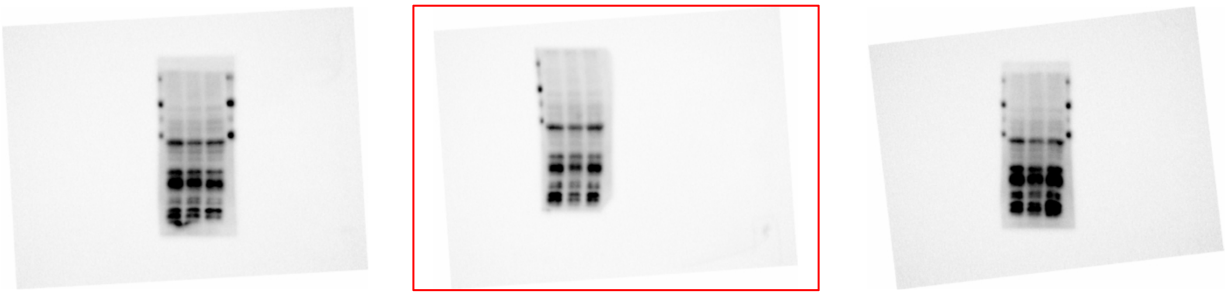

β-actin

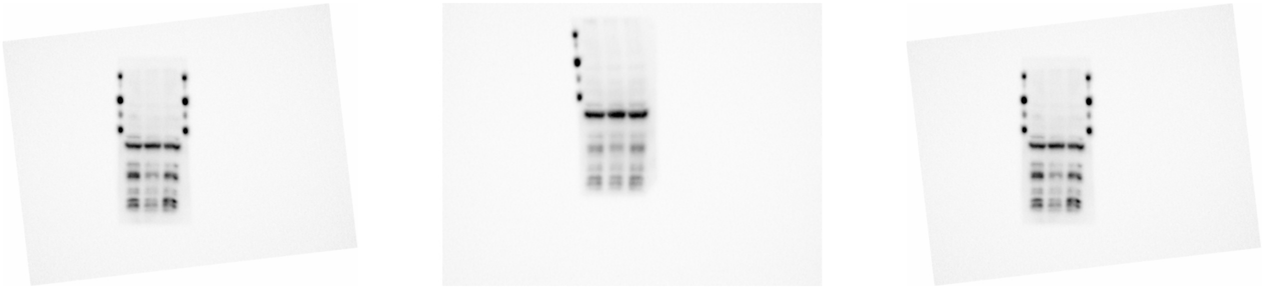

GLUD1

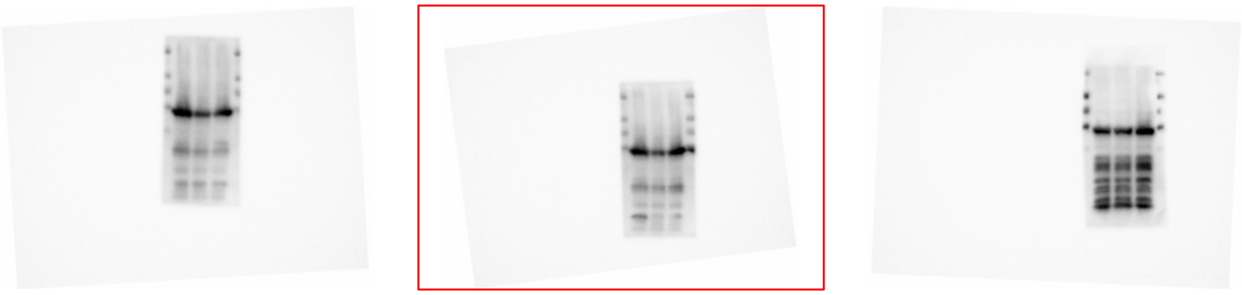

β-actin

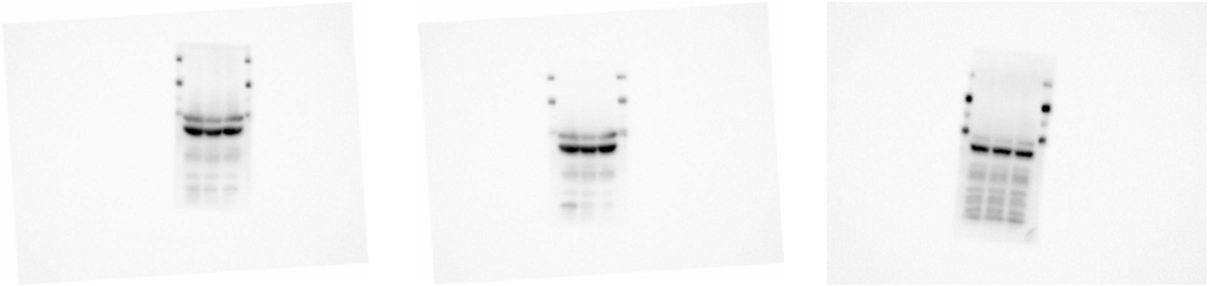

GLS1

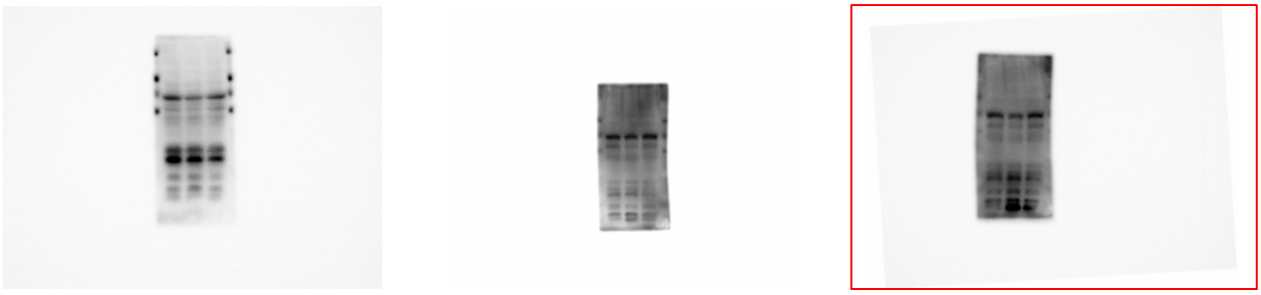

β-actin

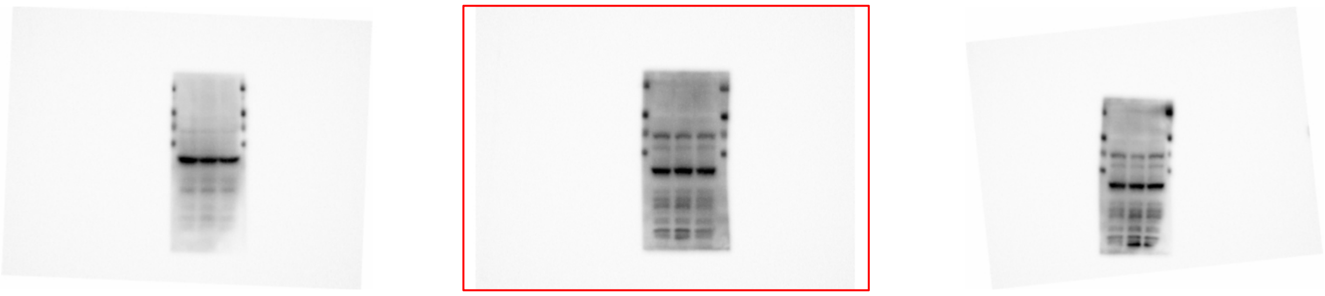

TGF-β

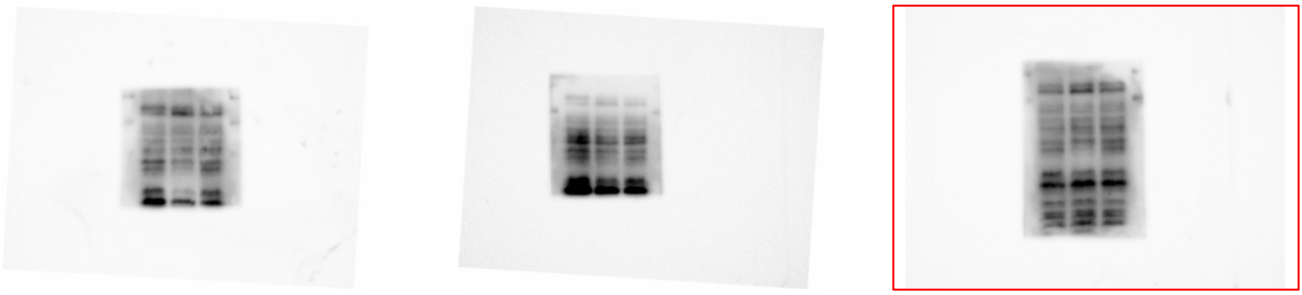

β-actin

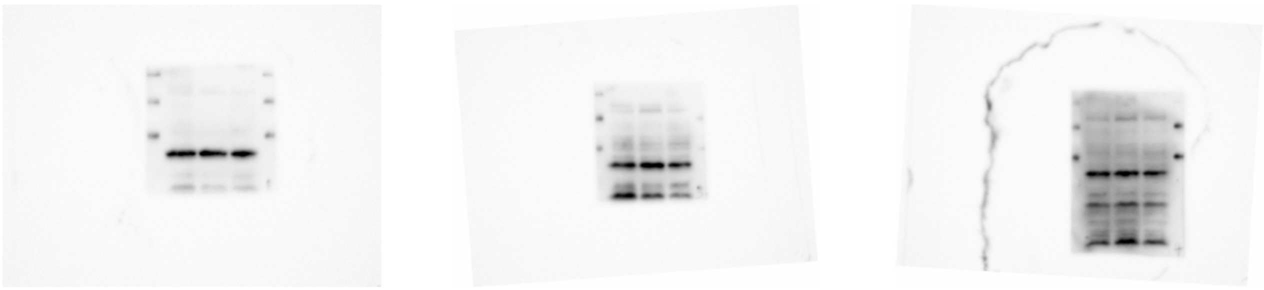

Fig.7E

p-AKT

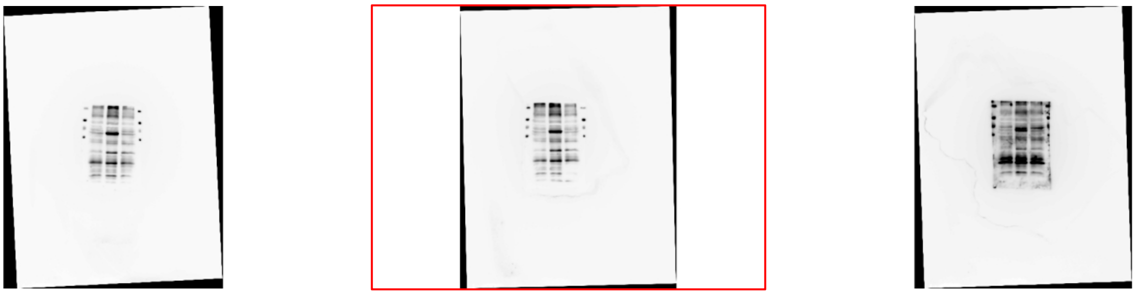

AKT

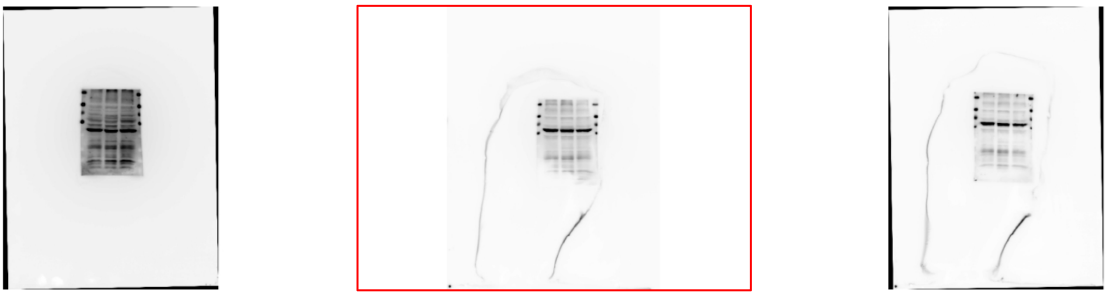

β-actin

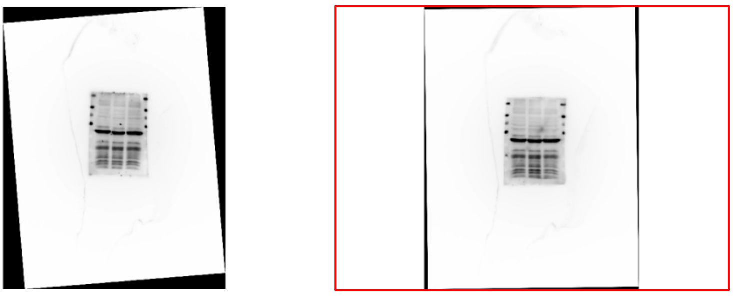

PD-L1

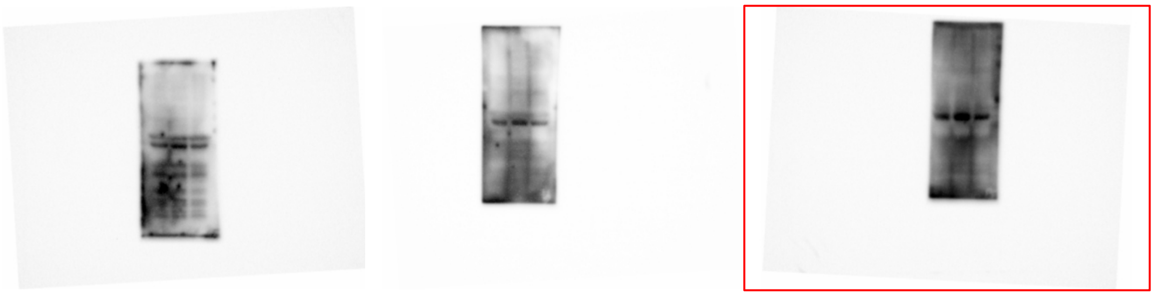

β-actin

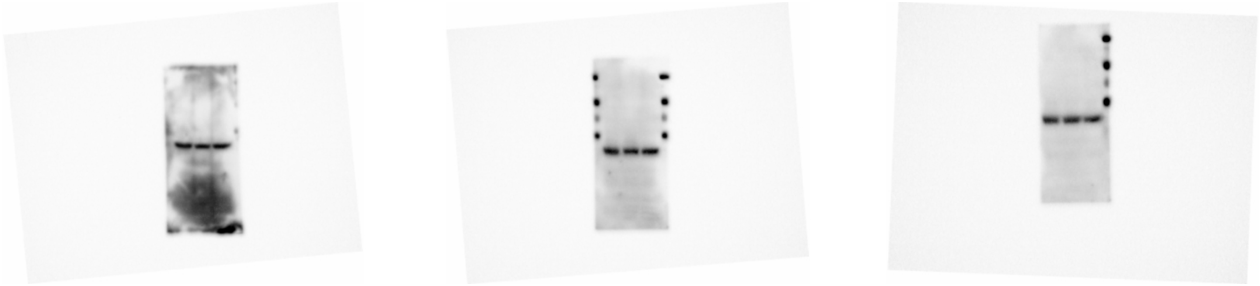

GLUD1

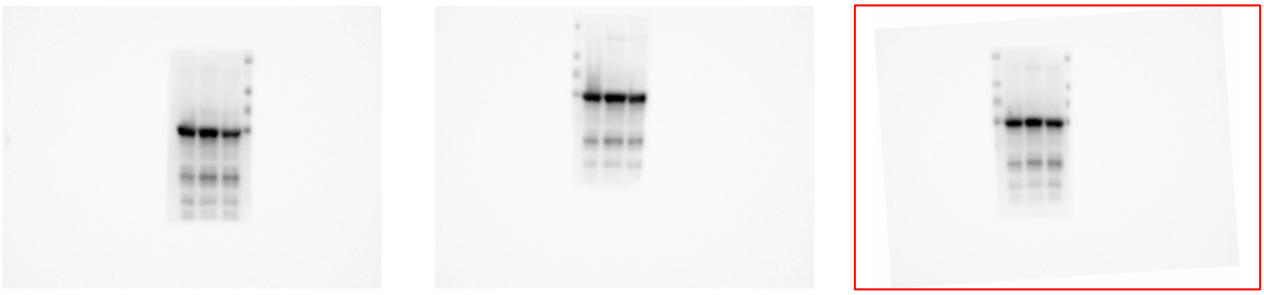

β-actin

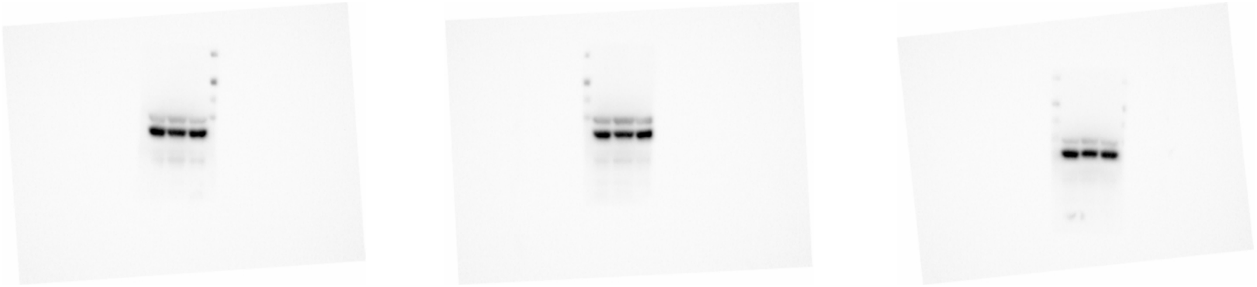

GLS1

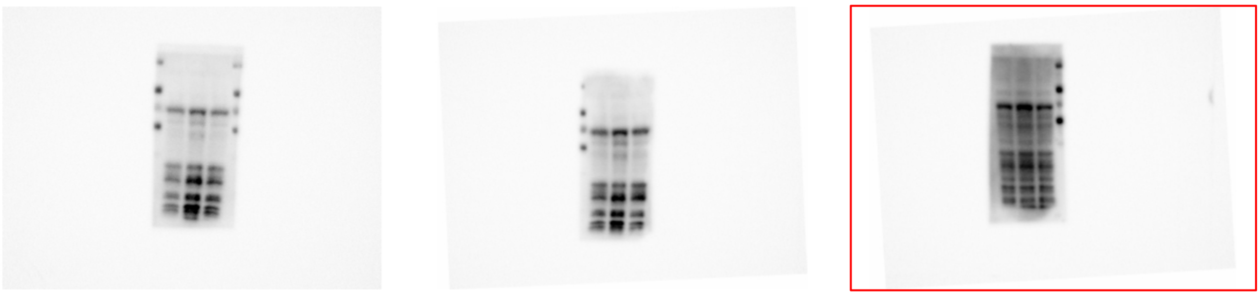

β-actin

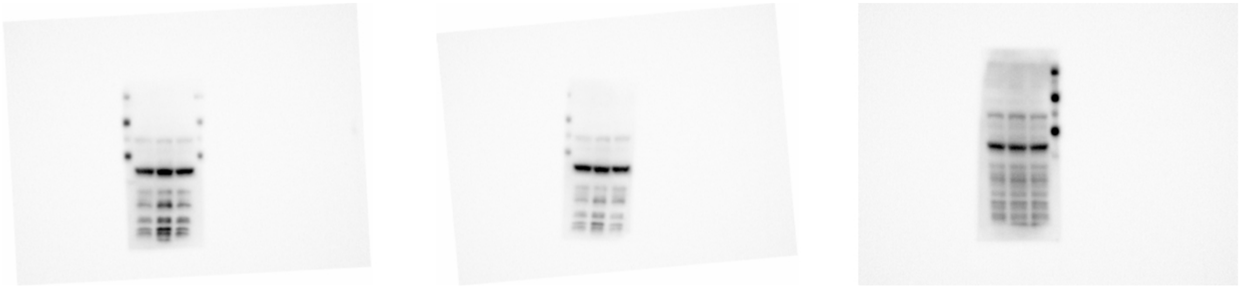

TGF-β

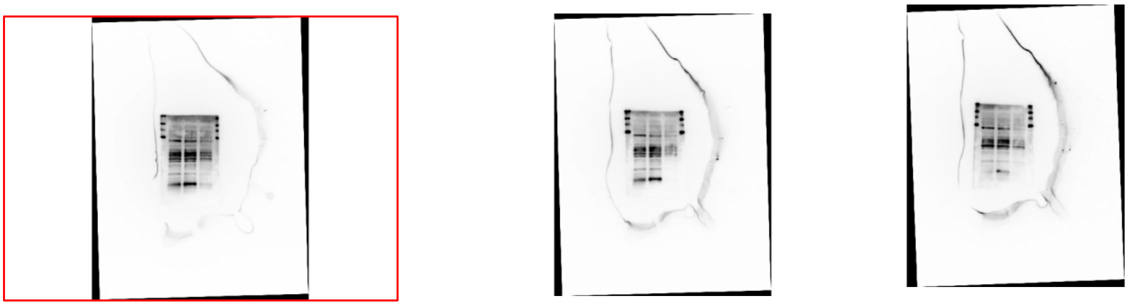

β-actin

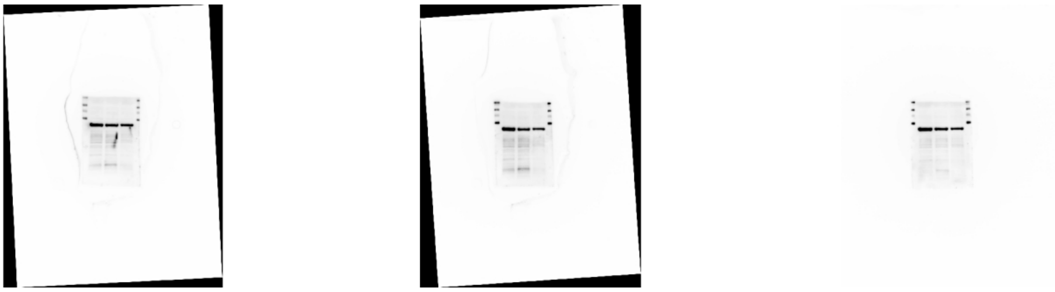

Supplementary Fig. A

PD-L1:

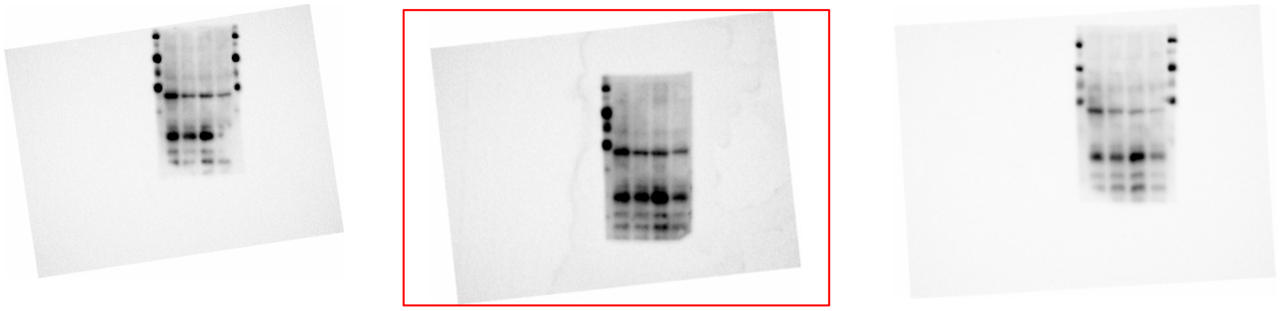

β-actin

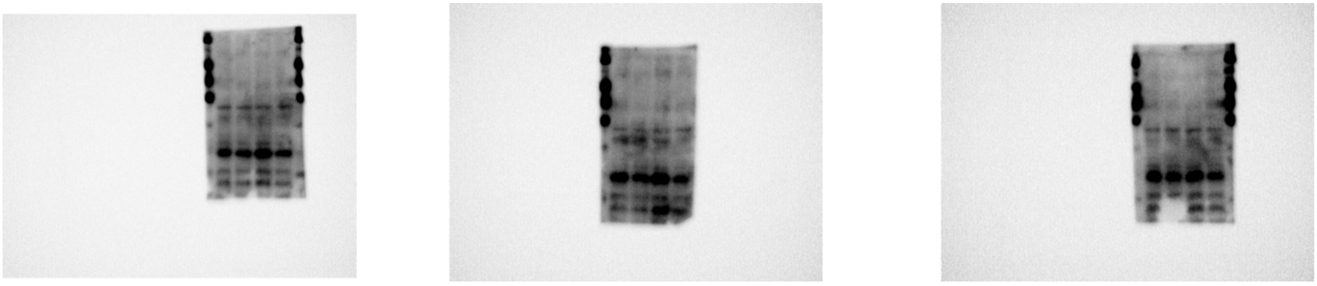

SMAD4:

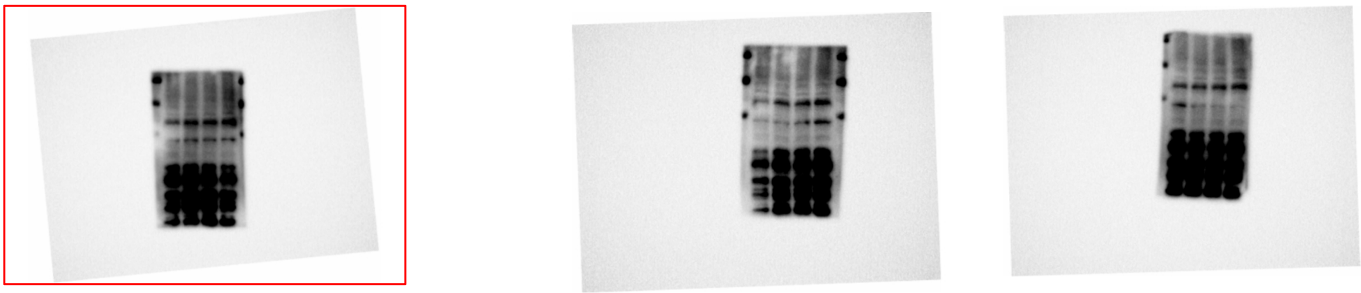

β-actin

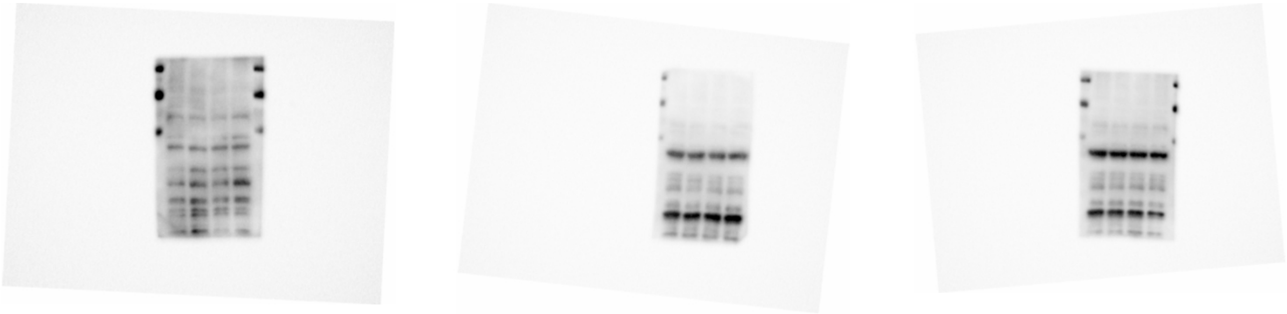

Vimentin:

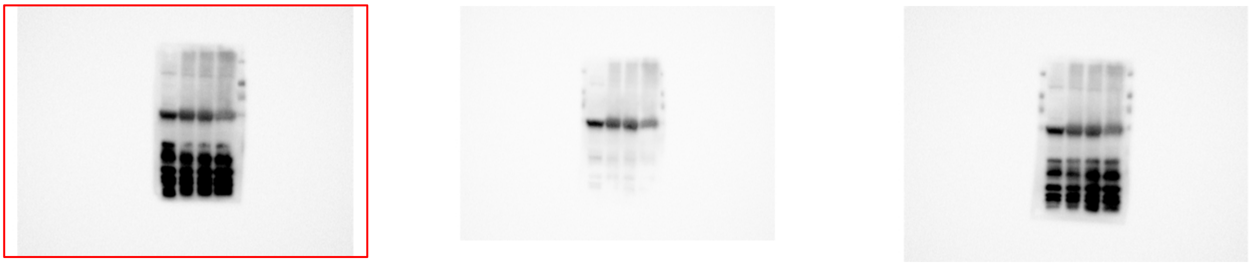

β-actin

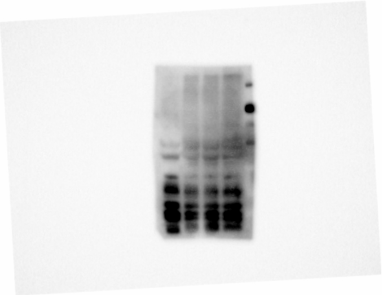
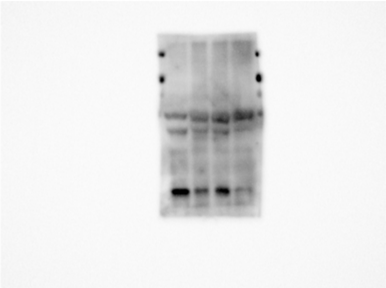
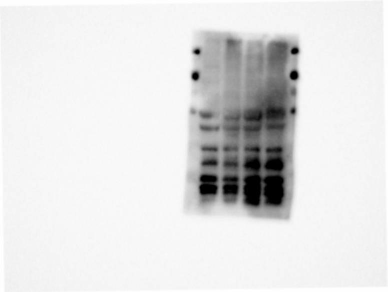

Snail

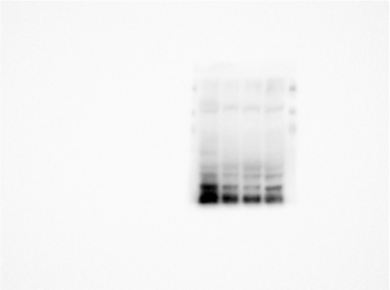
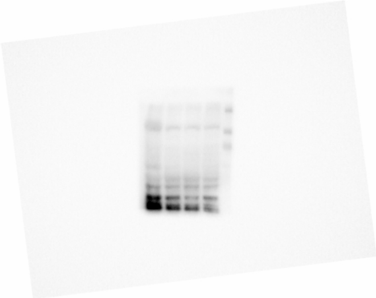
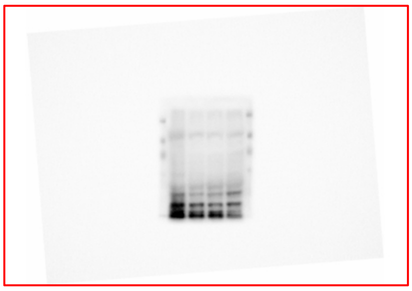

β-actin

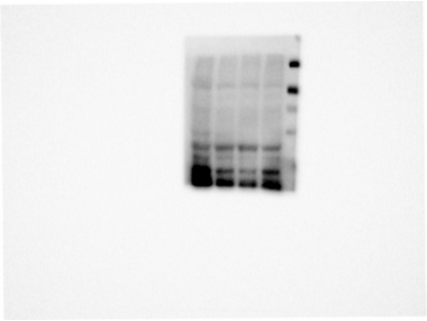
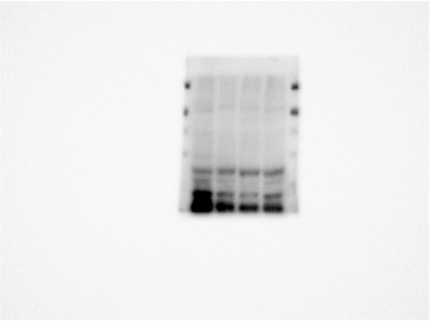
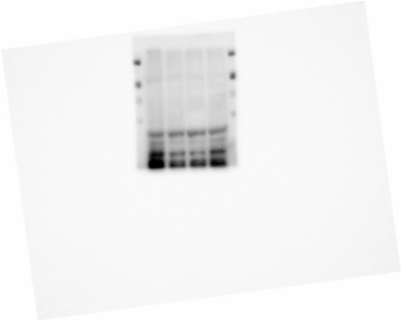

GLS1

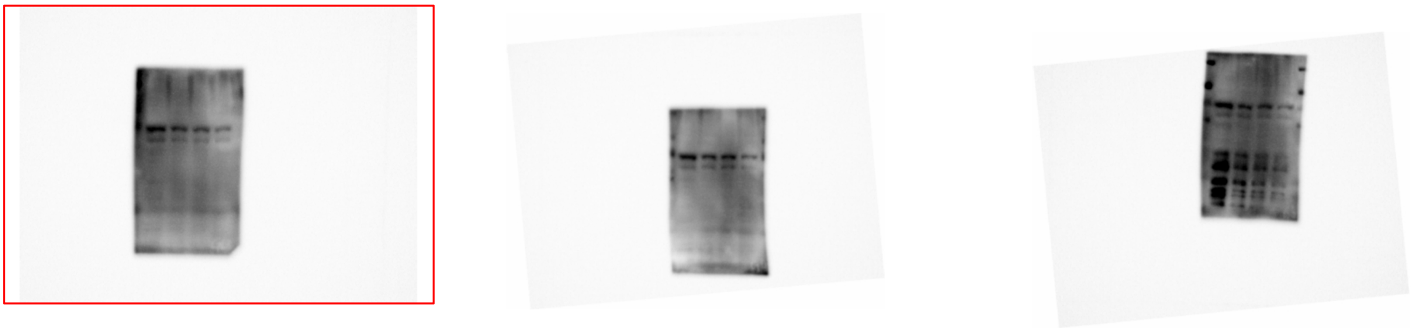

β-actin

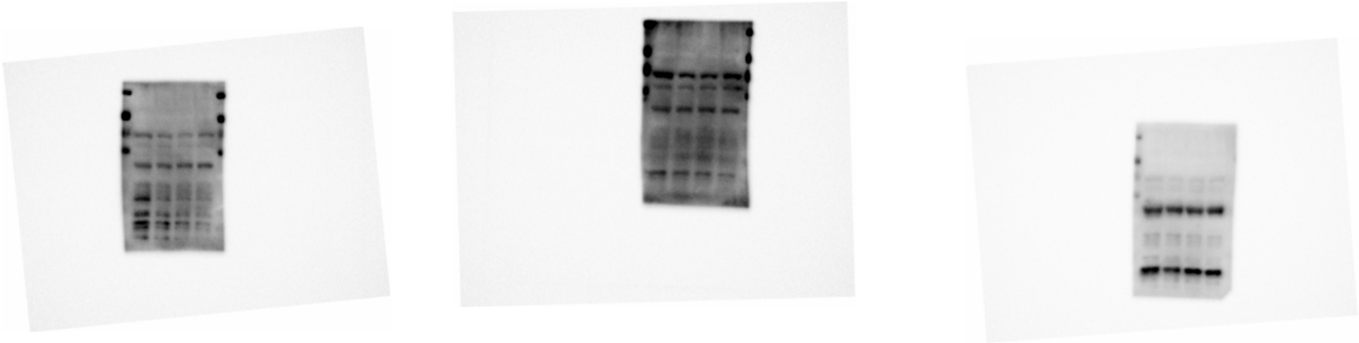

GLUD1

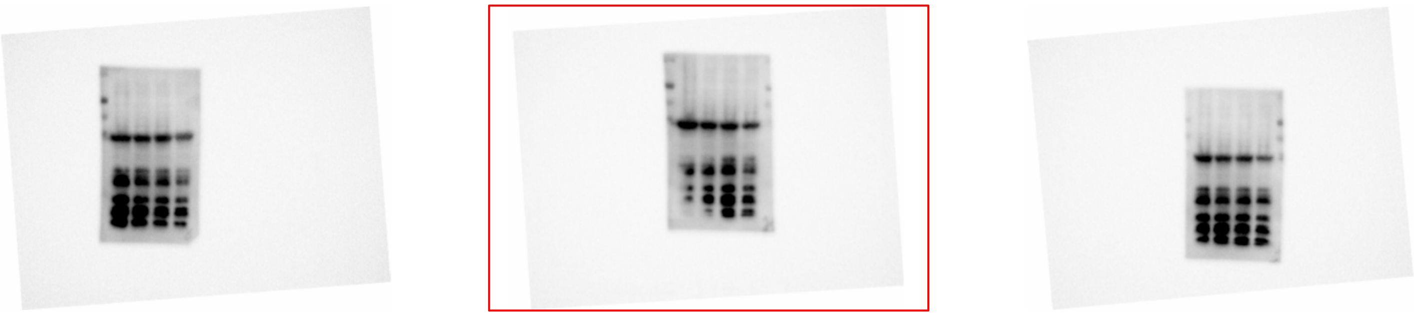

β-actin

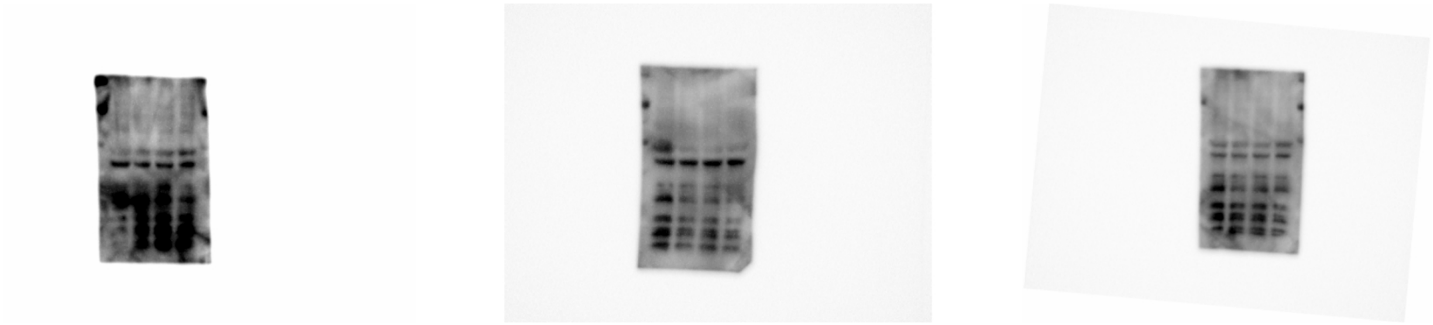

CXCL12

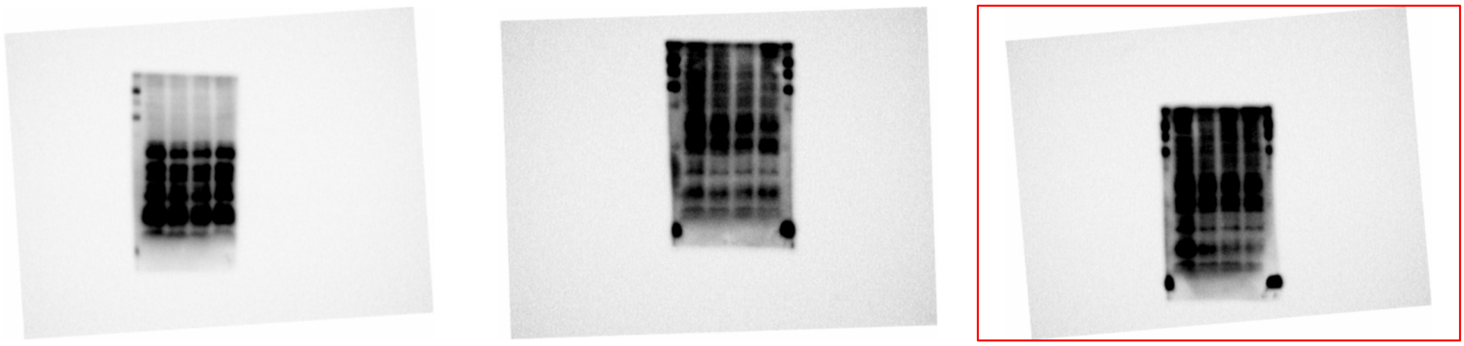

β-actin

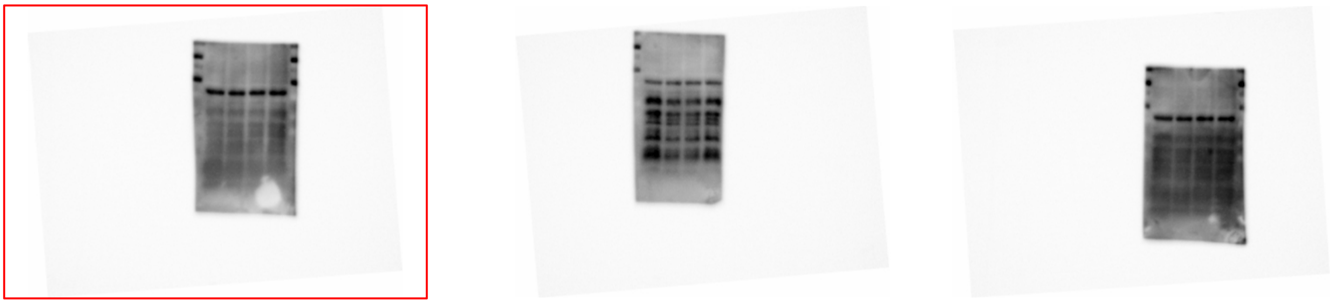

Supplement: Supplementary file 7 — Supplementary Western Blot [file 41419_2026_8795_MOESM7_ESM.pdf]
